# "The dead shall be raised": Multidisciplinary analysis of human skeletons reveals complexity in 19th century immigrant socioeconomic history and identity in New Haven, Connecticut

**DOI:** 10.1371/journal.pone.0219279

**Published:** 2019-09-09

**Authors:** Gary P. Aronsen, Lars Fehren-Schmitz, John Krigbaum, George D. Kamenov, Gerald J. Conlogue, Christina Warinner, Andrew T. Ozga, Krithivasan Sankaranarayanan, Anthony Griego, Daniel W. DeLuca, Howard T. Eckels, Romuald K. Byczkiewicz, Tania Grgurich, Natalie A. Pelletier, Sarah A. Brownlee, Ana Marichal, Kylie Williamson, Yukiko Tonoike, Nicholas F. Bellantoni

**Affiliations:** 1 Department of Anthropology, Yale University, New Haven, Connecticut, United States of America; 2 Department of Historical Anthropology and Human Ecology, Johann-Friedrich-Blumenbach Institute for Zoology and Anthropology, Georg-August-University Göttingen, Göttingen, Germany; 3 Department of Anthropology, University of Florida, Gainesville, Florida, United States of America; 4 Department of Geological Sciences, University of Florida, Gainesville, Florida, United States of America; 5 Department of Diagnostic Imaging, Quinnipiac University, Hamden, Connecticut, United States of America; 6 Bioanthropology Research Institute, Quinnipiac University, Hamden, Connecticut, United States of America; 7 Department of Anthropology, University of Oklahoma, Norman, Oklahoma, United States of America; 8 Department of Microbiology and Plant Biology, University of Oklahoma, Norman, Oklahoma, United States of America; 9 Independent Scholar, New Haven, Connecticut, United States of America; 10 Department of History, Central Connecticut State University, New Britain, Connecticut, United States of America; 11 Connecticut State Museum of Natural History, University of Connecticut, Storrs, Connecticut, United States of America; Seoul National University College of Medicine, REPUBLIC OF KOREA

## Abstract

In July 2011, renovations to Yale-New Haven Hospital inadvertently exposed the cemetery of Christ Church, New Haven, Connecticut’s first Catholic cemetery. While this cemetery was active between 1833 and 1851, both the church and its cemetery disappeared from public records, making the discovery serendipitous. Four relatively well-preserved adult skeletons were recovered with few artifacts. All four individuals show indicators of manual labor, health and disease stressors, and dental health issues. Two show indicators of trauma, with the possibility of judicial hanging in one individual. Musculoskeletal markings are consistent with physical stress, and two individuals have arthritic indicators of repetitive movement/specialized activities. Radiographic analyses show osteopenia, healed trauma, and other pathologies in several individuals. Dental calculus analysis did not identify any tuberculosis indicators, despite osteological markers. Isotopic analyses of teeth indicate that all four were likely recent immigrants to the Northeastern United States. Nuclear and mitochondrial DNA were recovered from three individuals, and these analyses identified ancestry, hair/eye color, and relatedness. Genetic and isotopic results upended our initial ancestry assessment based on burial context alone. These individuals provide biocultural evidence of New Haven’s Industrial Revolution and the plasticity of ethnic and religious identity in the immigrant experience. Their recovery and the multifaceted analyses described here illuminate a previously undescribed part of the city’s rich history. The collective expertise of biological, geochemical, archaeological, and historical researchers interprets socioeconomic and cultural identity better than any one could alone. Our combined efforts changed our initial assumptions of a poor urban Catholic cemetery’s membership, and provide a template for future discoveries and analyses.

## Introduction

The assessment of human skeletal remains requires the integration of biology, behavior, ecology and sociocultural anthropology [1–5]. While the human skeleton is subject to ultimate micro- and macroevolutionary forces, proximate factors such as environmental and cultural variables leave biomarkers on the durable yet plastic teeth and bone tissues [6–8].

The markers of human existence, from developmental interruptions and sexual dimorphism through senescence and death are recorded in dental perikymata, musculoskeletal origins and insertions and arthroses [9–12]. Chronic metabolic stressors, from infectious disease through physical exertion may also be preserved [2, 13–16]. Finally, traumatic injury with or without healing remains a primary focus for forensic and/or bioarchaeological research [17–19]. Cross-disciplinary efforts have generated increasingly nuanced techniques—digital radiography, dental calculus residue, genetic and isotopic analyses combine to provide rich details on health, ancestry, diet and geographic origin beyond macromorphoscopic bone review [20–26]. Such analyses are used to analyze and interpret cold cases [27–29] as well as skeletal remains with no provenience [30, 31].

Cultural ecology and socioeconomic status influence health, stress and disease [32]. Structural violence (defined as indirect negative consequences resulting from repression, racism and/or exclusion) results from social stratification and disparity [33–35] and is manifested via downstream health and fitness effects across individuals and generations [36–38]. While skeletal tissue is plastic, it retains characters reflecting the decedent’s social role and responses to structural violence, repetitive labor, and/or health/hygiene support [15–17, 39–43]. Epidemiological evidence from bones and teeth provides data on the etiology, expression, and mortality/survivorship of infectious diseases [2, 44–46].

Osteologists working with prehistoric populations associate biomarkers with predicted/hypothesized social systems [47, 48]. While the historical record provides data on social stratification, occupational stress and/or racial and religious discrimination [49, 50], incomplete or biased records hamper bioarchaeological conclusions [51–54]. Skeletal biomarkers indicating structural violence may be clearly inscribed on bone and teeth [55–57] and provide another line of evidence for historians, whether from agricultural [58–60] or industrial [42, 61, 62] populations. As a result, historians increasingly interact with other scientific disciplines to evaluate violence, disparity, population migration and cultural tradition diffusion [24, 63–67].

These multidisciplinary evaluations of biological, geochemical, and archival data generate more robust conclusions and nuanced narratives than any single approach. Here, we report the recovery and assessment of human skeletal remains from a forgotten 19^th^ century urban cemetery, hereafter referred to as the Yale-New Haven Four (YNH4). The biological, geochemical, and historical lines of evidence indicate that these individuals represent some of the earliest non-Irish Catholics within the city of New Haven, and their respective life histories associate with their ethnicity, health, disease and stress markers, cultural ecology, judicial action, and burial context.

## Methods

### Discovery, excavation and predictions

In the summer of 2011, the Yale-New Haven Hospital Emergency Room was undergoing renovation. On Friday, 09 July a hydraulic excavator dug a trench parallel to York Street. That weekend, rains eroded the exposed sediment. On Monday, 11 July the excavator operator noticed a human bone jutting from the trench cut. New Haven Police were contacted (Case #11–38996), and following review by the Office of the Connecticut Medical Examiner (ME # 11–09794) the remains were identified as originating from a historic/archaeological context. Connecticut State Archaeologist NFB then took over excavations. At the same time, retired New Haven Police Department and Yale New Haven Hospital Security Officer AG learned of the discovery, and was aware of the site’s history. AG and local historian HE provided a brief review as NFB initiated excavation with CT Office of State Archeology volunteers (including a team of high school students). GPA was contacted for technical assistance. As excavation progressed, NFB and GPA contacted the Pastor of St. Mary’s Church in New Haven. The Archdiocese of Hartford provided approval for excavation and multidisciplinary analyses, followed by repatriation and reburial. Yale New Haven Hospital authorities requested that excavation be limited to the exposed elements versus expanding our efforts.

**Fig 1:**
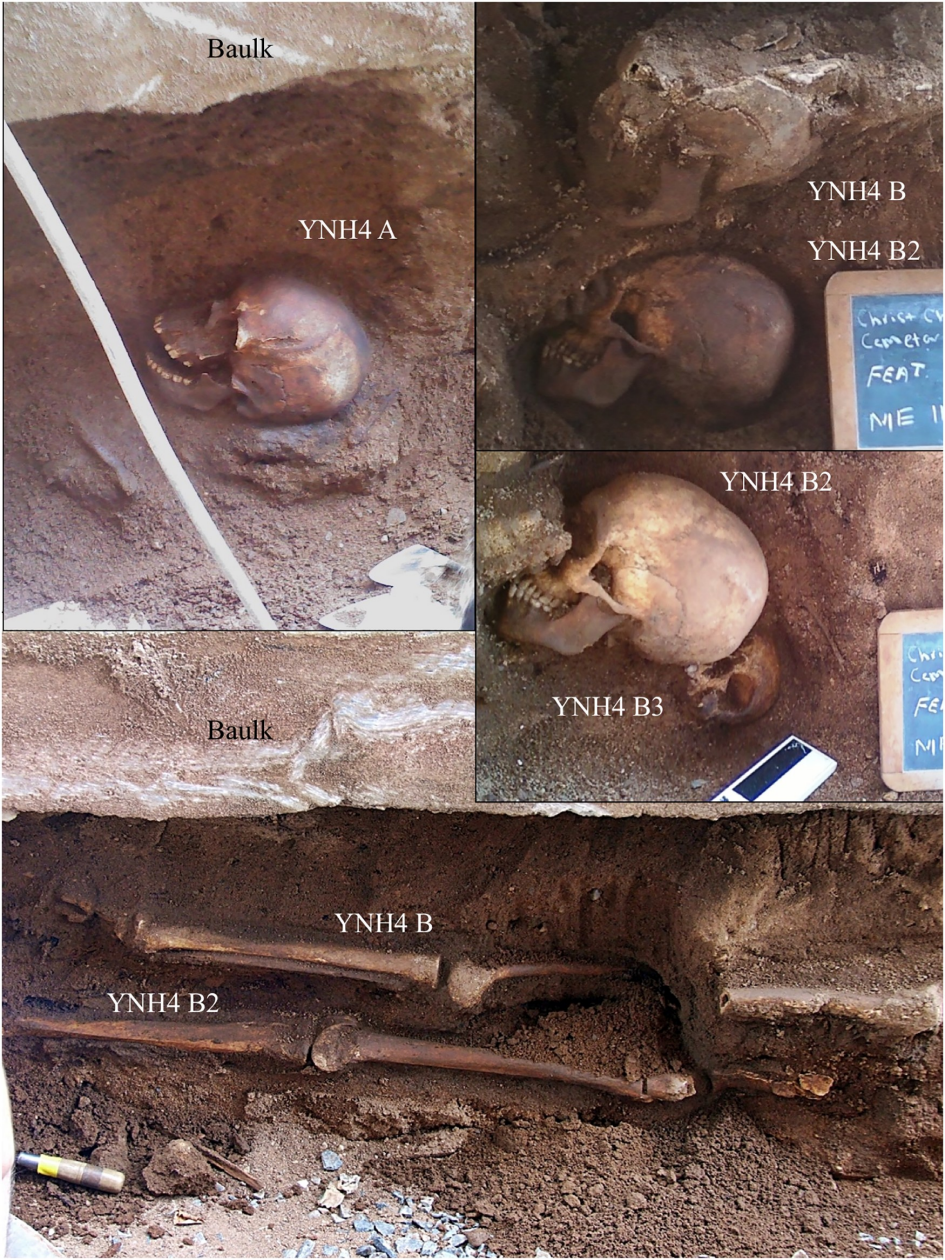
Clockwise from top left: YNH4 individual A in situ; B and B2 crania in situ; B2 and B3 crania in situ, B and B2 postcrania in situ. Note concrete baulk directly above burials.

The trench provided access to the human remains, but they were overlain by a concrete footing (~16cm thick, ~2.8m long x ~23cm wide). This required horizontal/sagittal excavation underneath the overlying baulk. As the first individual (designated YNH4 Individual A) was excavated, three additional individuals (YNH4 Individuals B, B2 and B3 respectively) were found immediately south of this burial, all vertically stacked (Fig 1). Each individual was excavated as a separate unit, to limit commingling or element swapping. Excavation proceeded via standard practices, using metal and/or bamboo tools for bone removal and backdirt screening using 1/8^th^ inch hardware mesh. Skeletal elements and artifacts were photographed *in situ* and then placed in clean backdirt and transported to the Yale Biological Anthropology Laboratories.

As the remains were recovered from a mid-19^th^ century Catholic Church, we hypothesized that the individuals were of Irish or Irish-American origin. Given the socioeconomic context of that time period, we predicted that they would show signs of musculoskeletal stress due to hard physical labor. Living in cities leads to higher risk of infectious disease exposure, and we predicted that infectious diseases (i.e., tuberculosis [*Mycobacterium tuberculosis*]) would be present in these individuals. We predicted that isotopic analyses would suggest geographic association within Irish and/or Connecticut regions, and that dietary markers would indicate access to readily available and inexpensive resources such as maize (a C_4_ plant) and/or potatoes (a C_3_ plant) [68].

### Analytical methods

#### Artifact methods

All samples were carefully washed with distilled water/methanol and dried, but not coated prior to analysis. Select samples were mounted using carbon tape. Samples were analyzed using a Zeiss EVO LS-15 scanning electron microscope equipped with an iXRF EDS-2000 X-ray energy dispersive spectrometer. Imaging was performed using voltage set at 5 kV; WD 20–25 mm. Elemental analysis voltage was set at 15 kV; WD 10.5 mm. Artifacts were compared with known comparative samples [69, 70].

#### Osteology and radiology methods

Skeletal elements were brushed clean, and select elements/bones were reconstructed using Acryloid B72. Remains were not washed or chemically cleaned due to their fragility. All elements were recorded and assessed for sex/age indicators following published standards [71] using Osteoware 2.4.037 [72]. Measurements of cranial and postcranial elements were performed with digital spreading and sliding Mitutoyo calipers. Multiple researchers measured the remains and recorded nonmetric characters [73] and pathologies, with no significant interobserver error. Ancestry estimation was performed using craniometric data via Fordisc 3.196 [74], comparing the YNH4 sample against populations of known sex and ancestry (19^th^ and 20^th^ Century European-Americans and 19^th^ C. Austrians, Hungarians, and Norwegians). We recorded all instances of health and disease indicators, trauma markers, and any other bony abnormalities following established methods [13, 14, 18, 75–78]. Each set of remains were radiographed following published standards [79] in the antero-posterior and supero-inferior planes using a Toshiba Kalare radiographic/fluoroscopic unit with a Konica Regius Model 110 Computed Radiography image reader. Elements were scanned using a Toshiba Aquilion X64 Computerized Tomography device. CT scans were taken at 0.5mm resolution with 100kV/450mA settings. Radiographs were evaluated and compared independently by Quinnipiac University radiography experts, and the results reviewed with the osteology team. Bone and tooth samples were excised from each individual for genetic and isotopic testing.

#### Genetic methods

Twelve bone and teeth samples from four individuals were shipped to the ancient DNA (aDNA) facilities at the Department of Anthropology, University of Göttingen, Germany (GoA) where they were stored at -20° C. All pre-amplification DNA procedures were carried out in dedicated clean lab ancient DNA facilities at GoA. All laboratory tools used to process the samples were either sterile and/or disposable, or decontaminated with full strength bleach (6%) and exposed to UV light for 1 hour before use. Pieces of the bone samples or tooth roots where cut using a drill with diamond tipped saw blade. Subsequently, all samples were immersed in full strength bleach for 2 minutes and then rinsed with ddH_2_0 and 70% Ethanol. Each side of the samples was then exposed to UV light for 10 min, and left to dry. Following the decontamination procedure, the samples were pulverized using a ball mill (Retsch 400, Germany). The samples were extracted following published protocols [80], utilizing 0.1 g of bone / tooth root powder for each sample. At least two extraction blank controls (EBCs) were used in every batch of extractions.

Both, mitochondrial and nuclear genetic data in this study was obtained via PCR-based experiments and analyzed employing capillary electrophoresis at GoA. To assess the authenticity of PCR- based results, we performed at least four independent amplifications from two independent DNA extracts each for every genetic marker, resulting in a minimum of 8 amplification results. We used a majority call to determine a consensus for each allele. Amplification success for each PCR reaction was checked using 2.5% Agarose gels. The EBCs were included in each amplification, as well as no template controls (NTCs) to monitor for contaminations.

To determine the mitochondrial haplotypes of the individuals, we analyzed a 388-bp fragment of the mitochondrial hypervariable region I [nucleotide position (np)16,021– np16,408 (rCRS)] using four overlapping primer pairs [81]. The amplicons were subsequently analyzed by direct sequencing of the heavy and light strands and analyzed as described elsewhere [81]. Each part of the HVR1 was amplified at least two times from each two DNA extracts. The consensus HVR1 sequence for each individual was then used to determine the mitochondrial haplotype, employing the HaploGrep 2 haplotype prediction tool [82], based on PhyloTree v17 [83].

We further amplified fourteen autosomal microsatellites (D13S317, D21S11, D18S51, TH01, D5S818, FGA, D9S1120, VWA, D16S539, D7S820, D3S1358, D2S1338, D19S433, D8S1179) and the sex specific locus Amelogenin for each the samples using two multiplex PCR reactions, to evaluate the direct genetic relationship of the individuals, as well as their genetic ancestry. PCR conditions, primer sequences and other analytic details are described elsewhere [80, 84]. We determined the minimal Y-chromosomal haplotype for the male individual B3 employing a multiplex PCR amplifying eight Y-chromosomal STRs (DYS 392, DYS 391, DYS 19, DYS 389I, DYS 390, DYS 389II, DYS 393, DYS 385) [85]. The STR allele results were then compared to the YHRD database (YHRD.org; release 54) to determine the geographic ancestry of the haplotype [86]. While not strictly associated with ancestry, we also determined hair and eye color of the individuals sampled using the HIrisPlex system following published amplification protocols [87]. Phenotypes for the HIrisPlex results were then determined using the online tool provided by the Department of Genetic Identification at Erasmus University Medical Center, Rotterdam, the Netherlands [87].

#### Isotopic methods

The same samples used for the genetic sampling were shipped and prepared at the University of Florida in the Department of Anthropology’s Bone Chemistry Laboratory and the clean lab facilities within the Department of Geological Sciences. Tooth enamel and bone were sampled using a Brasseler dental drill fitted with a round-end tapered diamond tipped bit with a ~4 mm cutting edge and maximum diameter of ~1.2 mm. Bits were cleaned between sampling episodes by soaking in methanol and sonicating in DDI-H_2_O. Exposed surfaces of enamel were abraded to remove discoloration and any adhering material. Dentin and tooth material below the cemento-enamel junction were removed from the enamel by drilling. ‘Cleaned’ enamel fragments were powdered using an agate mortar and pestle, and ~25 mg of powder transferred to a microcentrifuge tube, treated with a 2% bleach (NaOHCl) solution for eight hours to remove organics, then rinsed with DDI-H_2_O until neutral pH was obtained. Following this, each sample was pretreated with 0.2 M acetic acid (CH_3_COOH) for eight hours and rinsed to neutral, frozen and lyophilized. Teeth carbonate samples were measured by phosphoric acid reaction at 70°C in a Finnigan-MAT Kiel III carbonate prep device followed by online analysis using a Finnigan-MAT 252 isotope ratio mass spectrometer. Carbon (C) and oxygen (O) isotope data are relative to NBS-19 δ^13^C = 1.95‰ (±0.05‰) and δ^18^O = -2.2‰ (±0.1‰).

For heavy isotope analysis, Teflon vials were first cleaned in bulk with Versa Clean, followed by 24 hour 8N HNO_3_ and 24 h 6N HCl baths. Teflon vials were then rinsed multiple times with MilliQ H_2_O, and 3 ml 6N HCl was added to each vial individually. The vials with the added 6N HCl were then capped and refluxed overnight at 120° C on a hot plate. After the reflux, the HCl was discarded and each vial was rinsed with ultrapure (4x) H_2_O and dried under a laminar flow hood. Once dried, samples were weighed into the pre-cleaned Teflon vials and dissolved in the clean lab with 3 ml 50% nitric acid (HNO_3_). Vials were placed on a hot plate at 120° C and evaporated to dryness under a laminar flow hood.

Following published protocols [88], ion chromatography was used to separate strontium (Sr) and lead (Pb) from single aliquots. Lead was purified with a conventional hydrobromic acid (HBr) procedure on Dowex 1X-8 resin. Washes were collected and evaporated to dryness for subsequent Sr separation. Dried wash residues were dissolved in 3.5 N HNO_3_ and loaded onto cation exchange columns packed with Sr-selective crown ether resin (Sr- spec, Eichrom Technologies, Inc.) to separate Sr from other ions, following existing protocols [89].

Strontium ratios ^87^Sr/^86^Sr were measured using a Micromass Sector 54 thermal ionization mass spectrometer (TIMS). Strontium samples were loaded onto degassed tungsten filaments and run for 200 ratios at 1.5 V. Lead ratios (^206^Pb/^204^Pb, ^207^Pb/^204^Pb, and ^208^Pb/^204^Pb) were measured in solution using a Nu-Plasma multiple-collector inductively-coupled plasma-mass spectrometer (MC-ICP-MS) using the Tl-normalization technique [90]. ^87^Sr/^86^Sr is reported relative to NBS 987 with a published value of 0.71024 (±0.00003, 2σ) and ^20n^Pb/^204^Pb are reported relative to NBS 981 with published values for ^206^Pb/^204^Pb = 16.937 (±0.004, 2σ), ^207^Pb/^204^Pb = 15.490 (±0.003, 2σ), and ^208^Pb/^204^Pb = 36.695 (±0.009, 2σ). Trace element analyses were performed on an Element2 HR-ICP-MS in medium resolution with Re and Rh used as internal standards. Quantification of the results was achieved via external calibration using a set of gravimetrically prepared dilutions of commercial ICP-MS standards (SPEX CertiPrep, Inc.) The reported concentration values are better than ±5%.

At Yale University, select teeth with labial surface staining were examined using a portable X-ray fluorescence spectrometer (Bruker Tracer III-V^+^) to evaluate the elemental composition of residue. Surfaces with no other visible contamination were chosen. The device was factory calibrated against NIST standards and then was further checked against a known Duplex 2205 standard prior to each use in the laboratory. A yellow filter (0.001” Ti, 0.012” Al) was used and the instrument was set at 40 kV and 28 μAmps following Bruker USA recommendations (B. J. Kaiser, Bruker Corporation, pers. comm.). This focused the x-rays from 12 to 40 keV, allowing the instrument sensitivity to be focused on elements above Ca on the periodic table. The analysis was run for 180 seconds with the tooth lying as flat as possible on the analyzer window with a protector film provided by Bruker to minimize contamination. The resulting spectra were qualitatively compared against inorganic materials and studied using the ARTAX software.

#### Dental calculus methods

Dental calculus samples from each individual were collected by GPA using established protocols [91]. DNA extraction was performed at the University of Oklahoma’s Laboratories of Molecular Anthropology and Microbiome Research (LMAMR) in a dedicated ancient DNA (aDNA) laboratory facility following previously published protocols [92]. In brief, prior to extraction, the dental calculus samples were cleaned of surface debris by vortexing the specimens in 1 ml wash solution of 0.5M EDTA. Specimens were then digested in 1 ml of 0.5M EDTA solution on a rolling nutator at room temperature until fully decalcified (~ 48 hours). Cellular debris were pelleted by centrifugation at 13,000 rpm for 1 minute, and the supernatant extracted twice with a phenol, choloroform, and isoamyl alcohol solution (25:24:1), and a third time with chloroform only. The DNA was then isolated on a Qiagen MinElute column and eluted into 60 μl of EB buffer. A non-template negative extraction control was processed in parallel with the samples. DNA concentrations of the ancient DNA extracts were assessed using a Qubit fluorometer High Sensitivity Assay, allowing total DNA yields (nanograms [ng] of DNA per milligram [mg] of sample) to be calculated.

Illumina 16S rRNA amplicon libraries were prepared using a protocol modified for ancient samples [92]. Data produced using this protocol do not retain the quantitative proportions of the starting bacteria, but this method is a useful high-throughput approach for screening for the presence or absence of specific taxa of interest [92]. Universal primers U341F/534R targeting the 16S rRNA V3 region were selected, and Golay (12 bp) indexed reverse primers were employed to facilitate pooling during Illumina sequencing [93]. Throughout library preparation, negative non-template extraction controls and PCR reagent blanks were included and monitored for contamination. Contamination was not observed in any PCR reaction. Each PCR reaction was set up as follows: 5 μl of 5x Phusion buffer, 2.5 μl 2mM decontaminated dNTPs, 0.5 μl 10μM primer 341F, 1.0 μl 10μM primer 534R, 0.25 μl Phusion Hot Start II DNA polymerase (2 U/μl) and 5 ng of DNA template, and sufficient molecular grade water to reach a total reaction volume of 20 μl. PCR cycling conditions were as follows: enzyme activation at 98°C for 30 seconds, followed by 35 cycles of 98°C for 15 seconds, 52°C for 20 seconds, 72°C for 20 seconds, followed by a final 5 minute extension at 72°C. Each PCR reaction was performed in triplicate, and PCR success confirmed via gel electrophoresis. Amplified PCR products were observed for the dental calculus but not for the negative extraction control or PCR blank. For each sample, the PCR products of the three amplifications were pooled. The pools were then combined and purified using a Qiagen MinElute column, quantified using a NanoDrop spectrophotometer and size-selected using a PippinPrep (Sage Science). Prior to sequencing, the amplicon size distribution and successful removal of dimer peaks was confirmed using a Bioanalyzer High Sensitivity DNA assay. The amplicon libraries were sequenced on an Illumina MiSeq flow cell using v3 2x100 bp chemistry at the Yale Center for Genome Analysis.

Bioinformatic analysis was performed following the QIIME pipeline [94]. Paired forward and reverse reads were quality filtered (quality scores < 30 trimmed) and merged using the program PEAR [95]. Sequences with uncalled bases (Ns) were removed from subsequent analysis. Sequences were assigned to Operational Taxonomic Units (OTUs) following a closed-reference OTU protocol. A similarity threshold of 97% was used to assign sequences to OTUs with the QIIME formatted Greengenes 16S rRNA database (v.13_08, Aug 2013) as a reference. Samples were rarefied to 10,000 sequences and analyzed for microbial community composition. All sequences assigned at the L6 level to the genus *Mycobacterium* were then manually examined for sequence similarity to published *Mycobacterium tuberculosis* 16S rRNA reference sequences. Upon completion, the microbial genetic data were deposited into the NCBI Short Read Archive (SRA) under BioProject accession PRJNA505811.

#### Historical and archival methods

We examined 1850 United States Census records, New Haven Vital Records, Archdiocese of Hartford Archives, local church records and the Charles R. Hale Collection of Cemetery Inscriptions [96] for interment records and local/regional identity. All Christ Church burial records were sorted into sex (based on given names) and modified age classes [97]: Infant, 0–2 years; Preschool Child, 2–5; Child, 6–12; Adolescent, 13–18; Young Adult 19–30; Middle Adult, 31–45; Mature Adult; 46–65; and Elderly, 65+ years. Recorded causes of death were compared to modern medical definitions. Life expectancy and survivorship curves were calculated following published methods [98] and compared to published summary data from five United States sites: New Orleans, Louisiana (1785–1786) [99] and urban and rural cemetery data from 1830 through 1859: New York City (Marble Cemetery [41 ½ 2^nd^ Avenue location]) [100] and Clinton, New York; Wellesley and Natick, Massachusetts; Newberry, South Carolina [101]; and Nashville, Tennessee [102]. We also reviewed local/regional/international historical sources for contextual data on mid-19^th^ century New Haven demographic history, culture and immigration, and church/parish development.

All research was conducted and authorized by the Connecticut State Archaeologist (NEB) and the Connecticut Office of State Archaeology, and was approved by the Archdiocese of Hartford and the Yale New Haven Hospital. No permits were required for the described study. This research follows the guidelines and ethical considerations of the American Association of Physical Anthropologists and the Society for American Archaeology. Following inventory, assessment, data recording, imaging and sampling, the remains are to be repatriated to the Archdiocese of Hartford for reburial.

## Results

### Recovery

**Fig 2:**
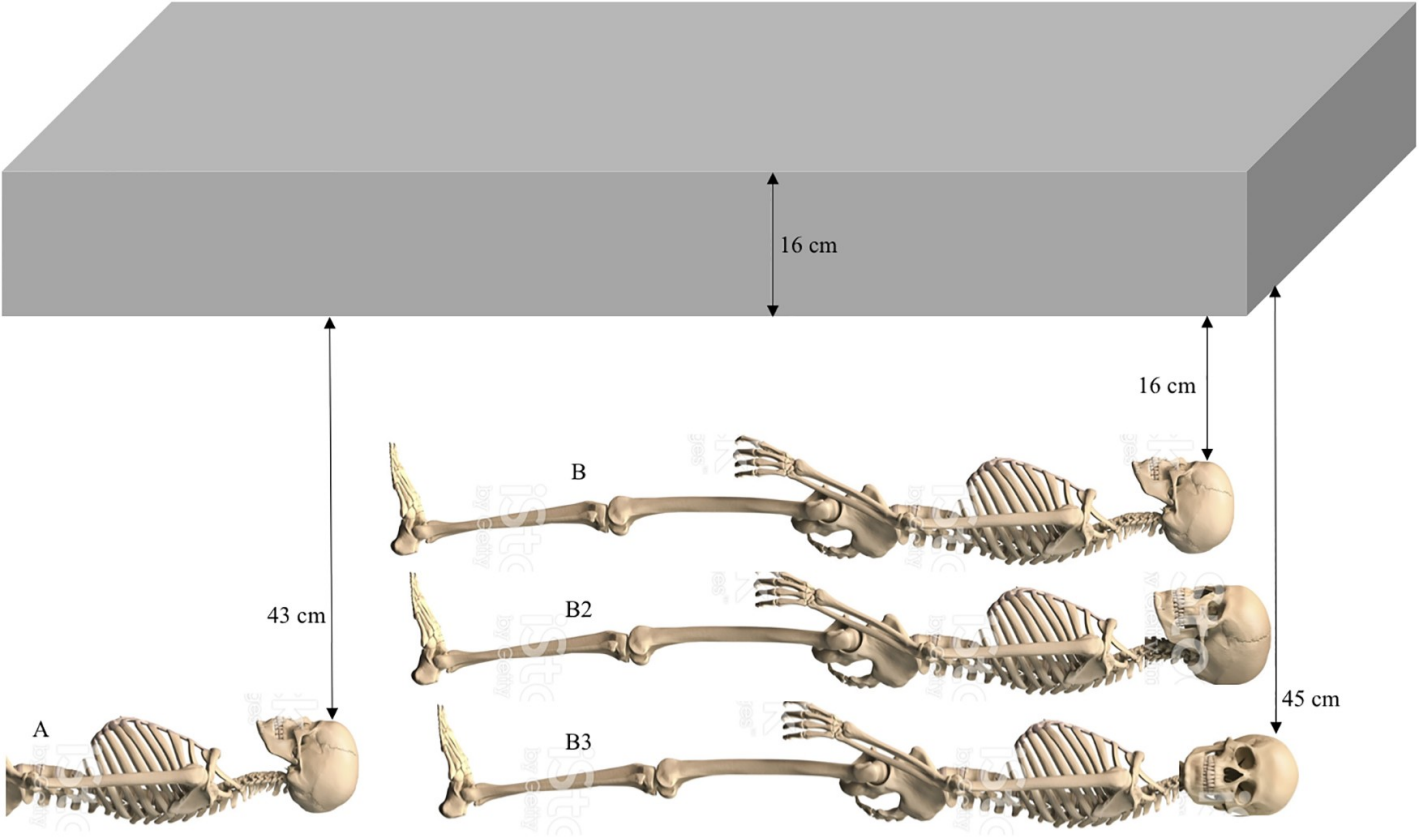
Schematic diagram of the YNH4 burials. See text for details.

Four individuals were recovered—two adult males, and two adult females. Soil pH levels were between 7.2 and 8.1, unusually high for Connecticut soils (NFB, pers. comm.). The stratigraphic integrity of each burial indicates that each individual was placed in a separate coffin but three (Individuals B through B3) were in the same grave shaft. Each individual was buried via traditional Christian practices, with individual coffins and burial shrouds (based on presence of wood fragments, nails and shroud pins). Bodies were oriented East-West with limbs extended. Each burial was discrete with coffin hardware recovered in between skeletal remains (Fig 2). Individual A’s lower body was not present due to the pouring of a concrete pillar as part of 1970s-era YNNH renovations. The concrete baulk directly over Individual B was part of this renovation. Individual B2 was immediately beneath individual B and had better preserved elements. Individual B3 was the lowest interment and directly below B2, and has the most complete and least damaged skeleton.

**Fig 3:**
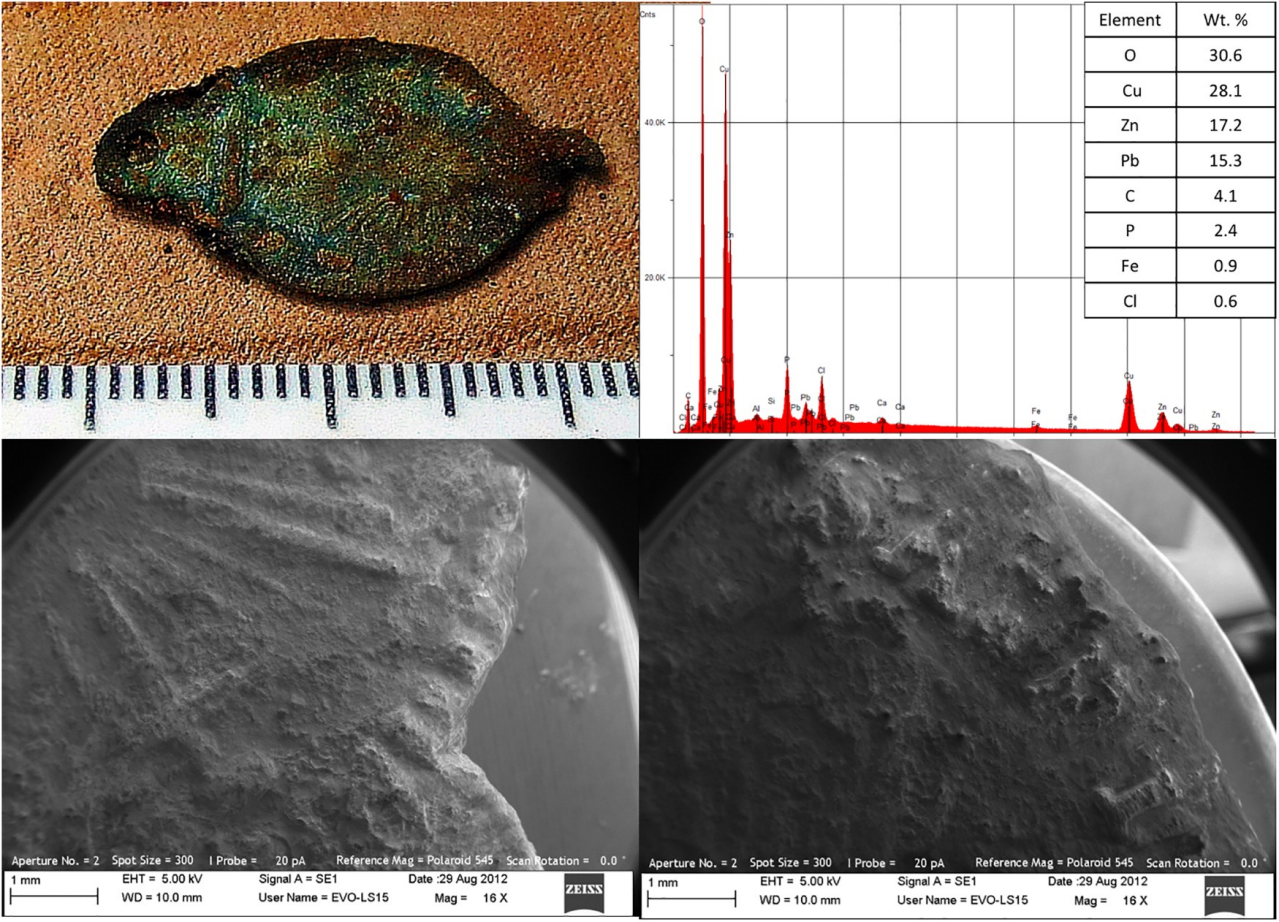
Clockwise from top left: Rosary centerpiece found with individual B3; elemental profile; and details of rosary word and motif.

Very few artifacts were recovered. All bodies were in coffins constructed of relatively soft wood (i.e., pine). Multiple copper alloy shroud pins, square coffin nails (N = 21) and thumb screws (N = 4) were recovered as well as multiple wood fragments. The thumb screws are associated with the shallowest burial (Burial B) and first appear in funerary trade catalogues around 1880 [70]. The other coffin hardware is consistent with mid-19^th^ century manufacture. The rosary centerpiece recovered with Individual B3 (N = 1) is constructed of copper alloy, and was manufactured via mechanical press—a single English word (“SAINT”) is visible (Fig 3). No other features can be associated to a specific manufacturer or time period.

### Archival data

These individuals were interred in the cemetery associated with Christ Church, the first Roman Catholic Church in New Haven and the second in the state of Connecticut. The church and cemetery were built in 1834 to cater to the rapidly expanding Irish immigrant community [103]. Archival records list over 600 burials, including individuals from towns outside of New Haven. Proper names, ages and cause of death are available for multiple individuals; almost all individuals have Irish/Gaelic/Anglo-Saxon surnames (S1 Table). The cemetery received burials steadily until 1854, when the larger St. Bernard’s Catholic cemetery was consecrated (and remains active today). Interments at Christ Church fell dramatically after this date.

Christ Church was re-consecrated as St. John’s Catholic Church in 1858. Sometime between 1869 and 1898, the Christ Church headstones were removed from the St. John’s grounds, and placed at St. Bernard’s. As St. John’s parishioners, administrators and archivists moved, transferred or died, awareness of the cemetery (now missing all headstones) seemed to disappear. In 1969, the church was sold to Yale University and through 1971, the grounds were cleared for additions to Yale New Haven Hospital [104–106]. There are no references to a cemetery in any available real estate transactions.

**Fig 4:**
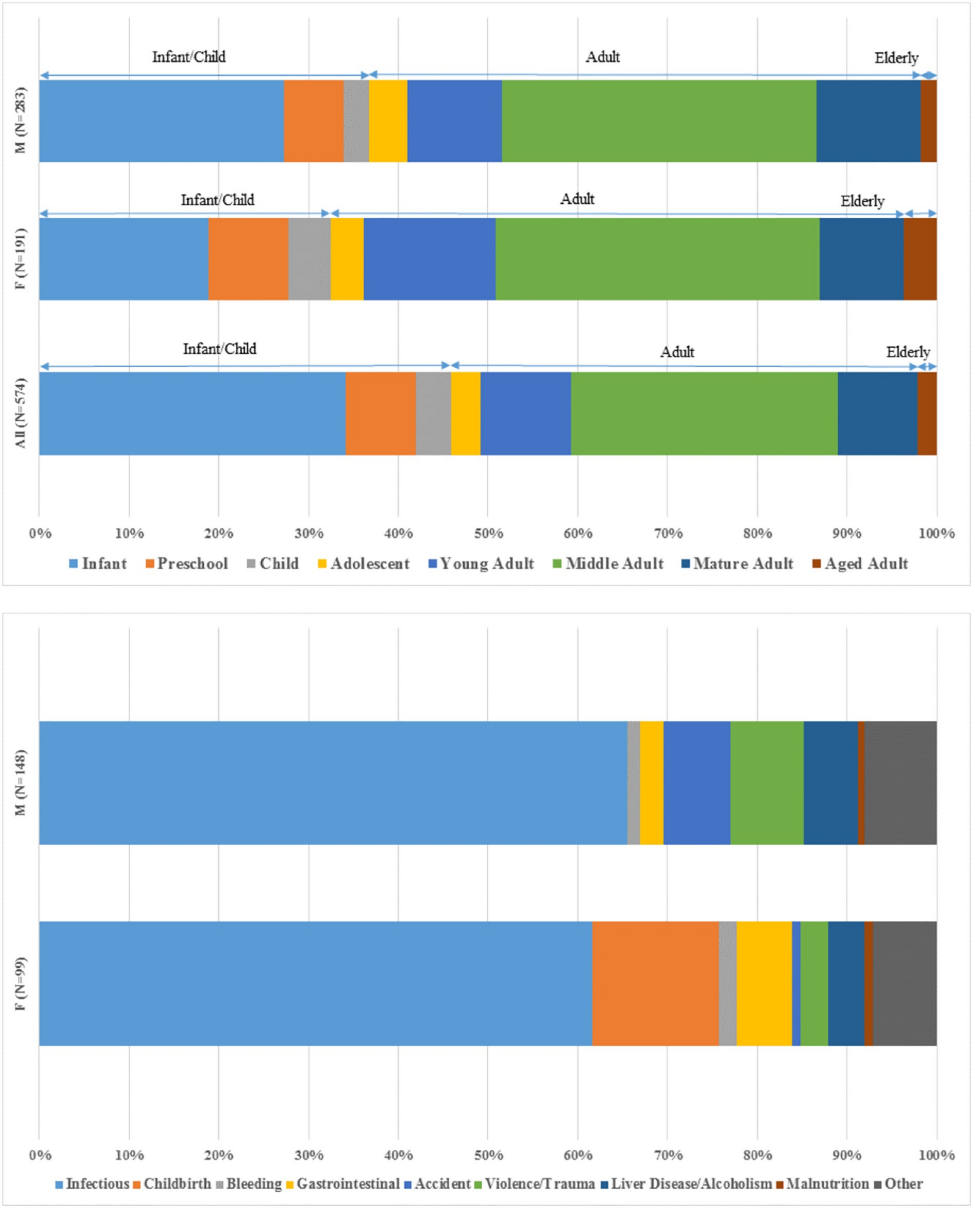
**(a) Demographic distribution of the Christ Church burials**. Note that individuals of unknown age (N = 31) are not included. **(b) Recorded causes of death for Christ Church burials (males and females aged 13–45)**. All data from S1 Table.

605 burial records were transcribed from available historic archives. As expected for a church catering to a relatively small and socioeconomically constrained 19^th^ century urban population, the burials reflect high infant and child mortality (46% of all dead, Fig 4a). Male infant/child mortality (37%) is slightly higher than females (32%) and more females survived to elderly ages than males (4% versus 2%, Fig 4a). For individuals likely participating in the industrial workforce (ages 13 through 45), infectious disease remains the most common cause of death, especially for males (Fig 4b). Childbirth, hemorrhaging and gastrointestinal illnesses represents 22% of female cause of death, and are associated with the relatively higher death rate for young females (Fig 4b). Accidents, violence/trauma and liver disease/alcoholism comprise 22% of male mortality (Fig 4b).

**Fig 5:**
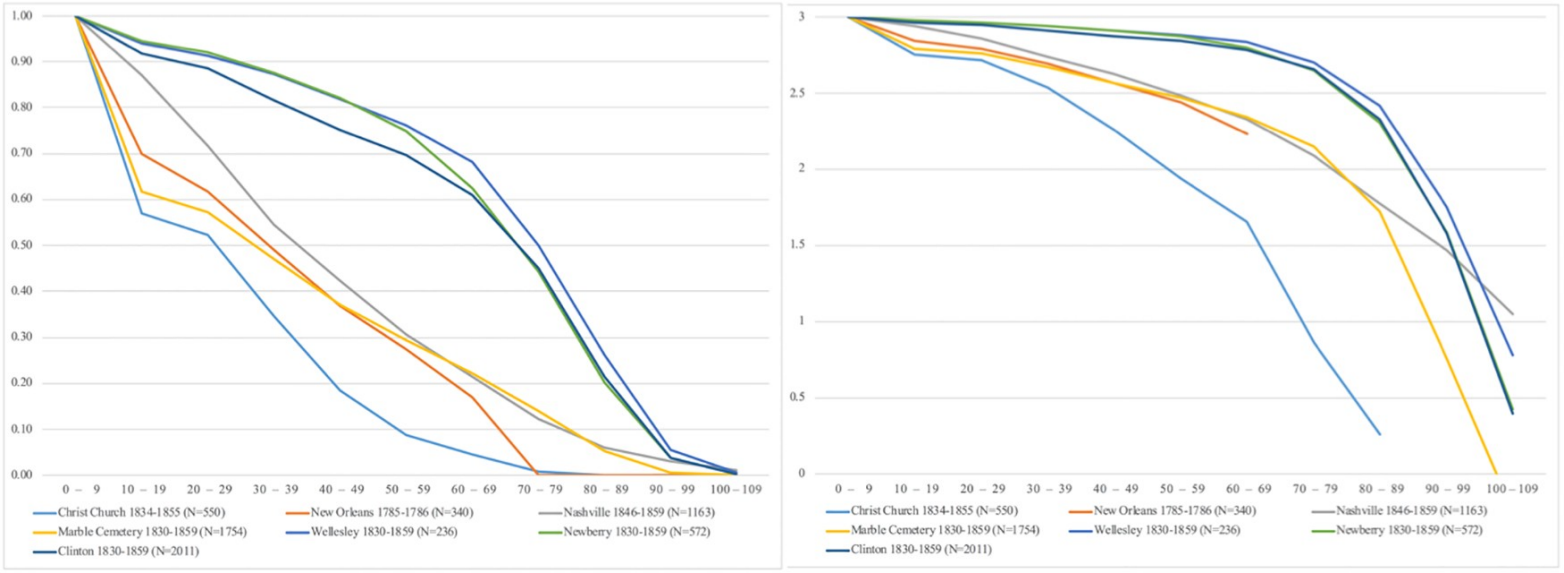
Christ Church demographic profiles. Left: percentage surviving (l(x)) per 10-year age classes in comparison to contemporaneous urban and rural cemeteries. Right: Log survivorship (l(x)) curves. See Table 1 for values.

Comparisons of Christ Church burial records’ survivorship and life expectancy shows very high infant and child mortality and lower life expectancy compared to contemporaneous urban and rural cemeteries (Fig 5; Table 1). Distribution patterns are most similar to urban cemeteries with people of lower socioeconomic status: 18^th^ Century New Orleans, 19^th^ Century Nashville City Cemetery’s African-American burials and New York City’s Marble Cemetery burials (Fig 5; Table 1).

**Table 1:** Percentage and log survivorship and life expectancy for multiple cemeteries. See text for details.

Age Class		0–9	10–19	20–29	30–39	40–49	50–59	60–69	70–79	80–89	90–99	100–109
Urban												
Christ Church 1834–1855 (N = 550)	%	1.00	0.57	0.52	0.35	0.18	0.09	0.05	0.01	0.00	0.00	0.00
	log l(x)	3.00	2.76	2.72	2.54	2.26	1.94	1.66	0.86	0.26	0.00	0.00
	Expectancy	22.64	25.89	17.86	14.42	12.72	11.25	7.00	7.50	5.00	15.00	5.00
New Orleans 1785–1786 (N = 340)	%	1.00	0.70	0.62	0.49	0.37	0.27	0.17	0.00	0.00	0.00	0.00
	log l(x)	3.00	2.85	2.79	2.69	2.57	2.44	2.23	0.00	0.00	0.00	0.00
	Expectancy	31.21	32.44	26.10	21.53	17.08	11.24	5.00	0.00	0.00	0.00	0.00
Nashville 1846–1859 (N = 1163)	%	1.00	0.87	0.72	0.54	0.42	0.31	0.21	0.12	0.06	0.03	0.01
	log l(x)	3.00	2.94	2.85	2.74	2.63	2.49	2.33	2.09	1.77	1.47	1.05
	Expectancy	37.94	32.86	28.86	26.41	22.56	19.21	15.40	13.17	11.81	8.82	5.00
Marble Cemetery 1830–1859 (N = 1754)	%	1.00	0.62	0.57	0.47	0.37	0.29	0.22	0.14	0.05	0.01	0.00
	log l(x)	3.00	2.79	2.76	2.67	2.57	2.47	2.34	2.15	1.72	0.76	0.00
	Expectancy	32.44	39.44	32.11	28.11	24.22	19.29	13.97	9.20	6.20	6.00	5.00
Wellesley 1830–1859 (N = 236)	%	1.00	0.94	0.91	0.87	0.82	0.76	0.68	0.50	0.26	0.06	0.01
	log l(x)	3.00	2.97	2.96	2.94	2.91	2.88	2.83	2.70	2.42	1.75	0.78
	Expectancy	63.12	56.83	48.43	40.32	32.65	24.76	17.09	11.44	7.38	6.07	5.00
Rural												
Newberry 1830–1859 (N = 572)	%	1.00	0.94	0.92	0.88	0.82	0.75	0.62	0.44	0.20	0.04	0.00
	log l(x)	3.00	2.98	2.96	2.94	2.91	2.87	2.79	2.65	2.30	1.59	0.42
	Expectancy	61.21	54.54	45.75	37.85	30.09	22.50	16.00	10.45	7.05	5.69	5.00
Clinton 1830–1859 (N = 2011)	%	1.00	0.92	0.89	0.82	0.75	0.70	0.61	0.45	0.21	0.04	0.00
	log l(x)	3.00	2.96	2.95	2.91	2.88	2.84	2.78	2.65	2.33	1.58	0.40
	Expectancy	58.84	53.59	45.45	38.88	31.74	23.86	16.60	10.66	6.90	5.65	5.00

### Osteological indicators of ancestry, health and disease

**Fig 6:**
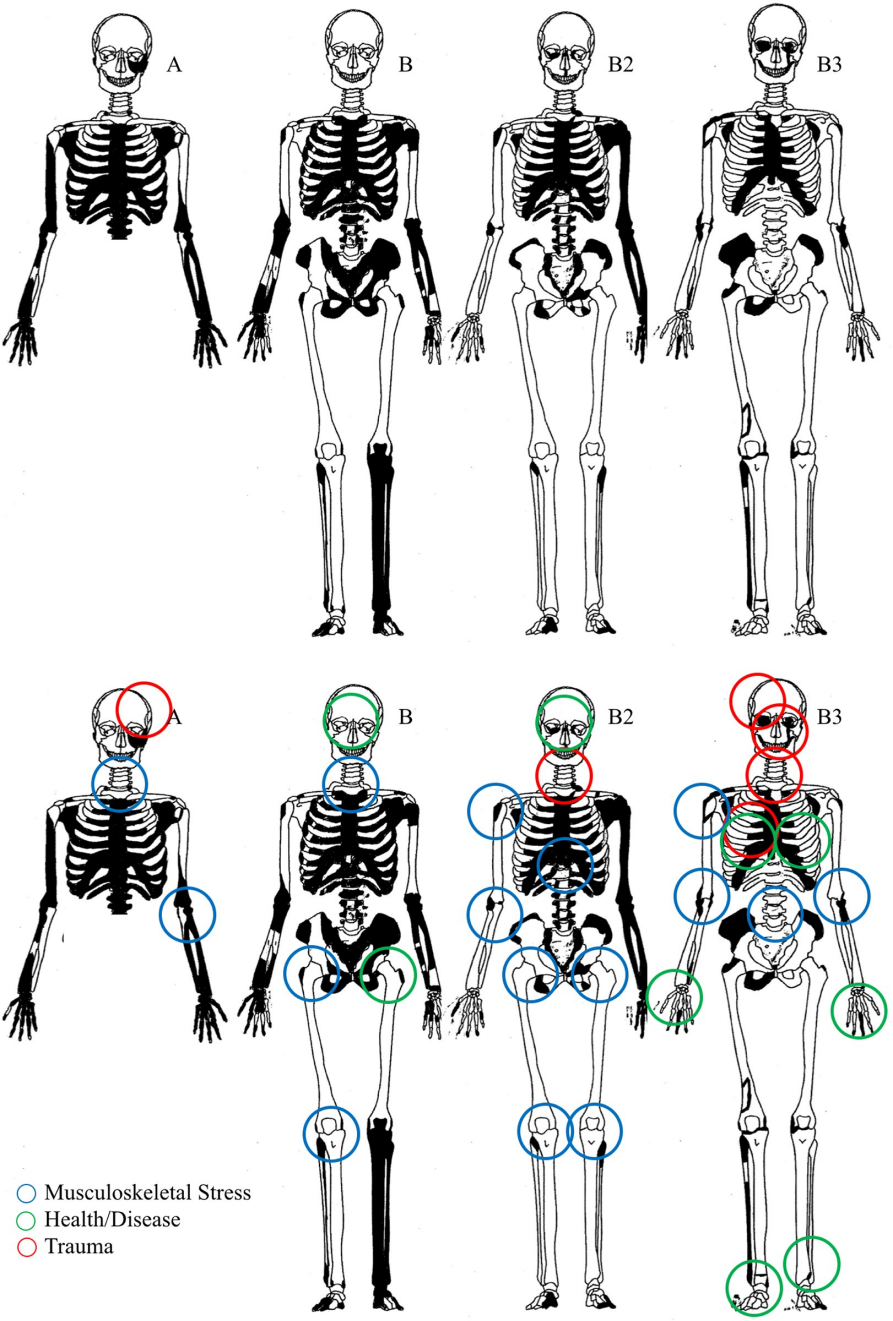
YNH4 element representation (top) and pathology distribution (bottom). Black = missing. Circles represent location and etiology of pathologies. Note YNH4 A is missing lower body elements. See text for details.

**Fig 7:**
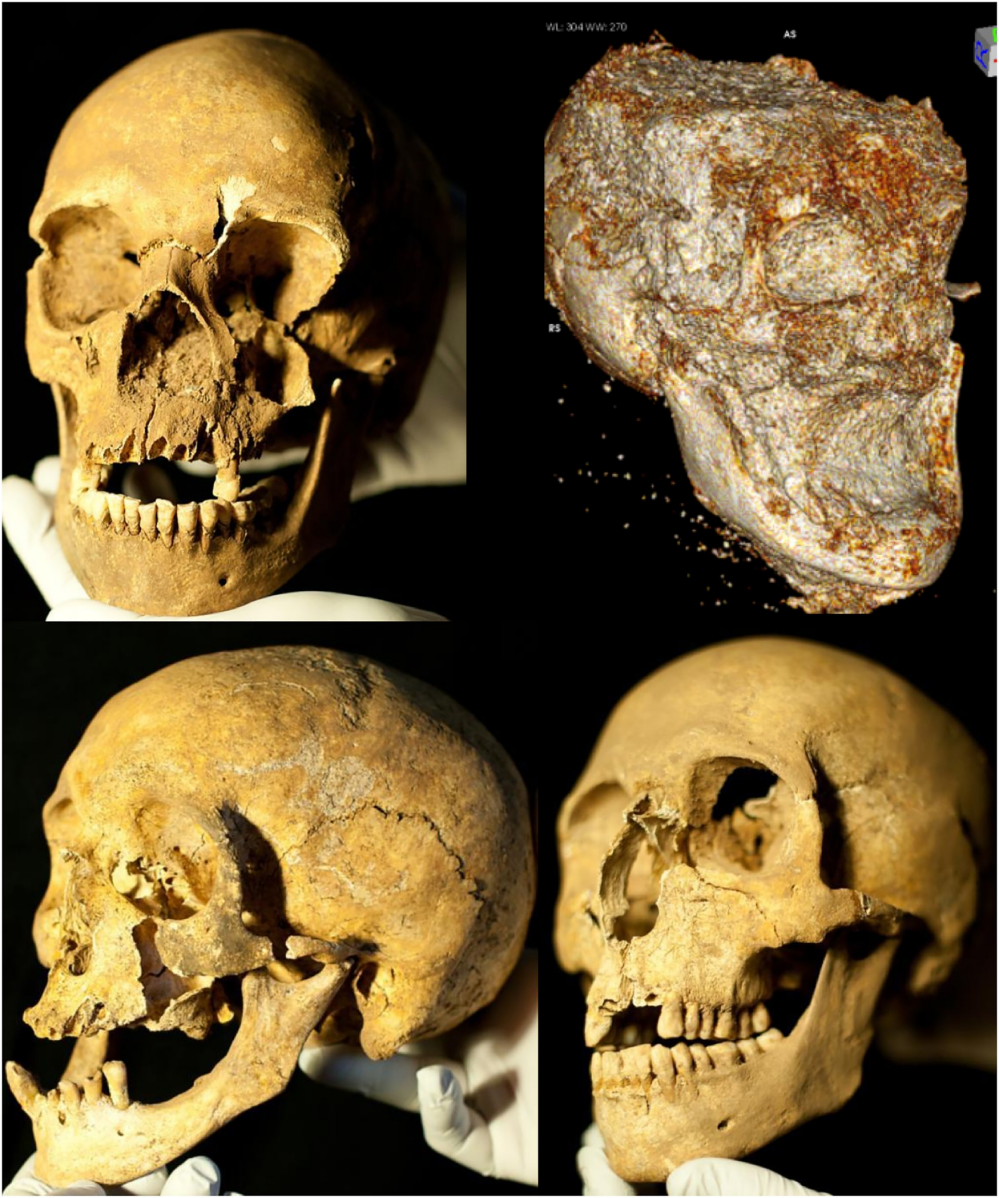
YNH4 Skull preservation. Clockwise from top left: YNH4 Individual A; B (CT scan due to condition); B2 and B3. Photos by Stephanie Anestis; radiograph by GJC, TG and NAP.

**Fig 8:**
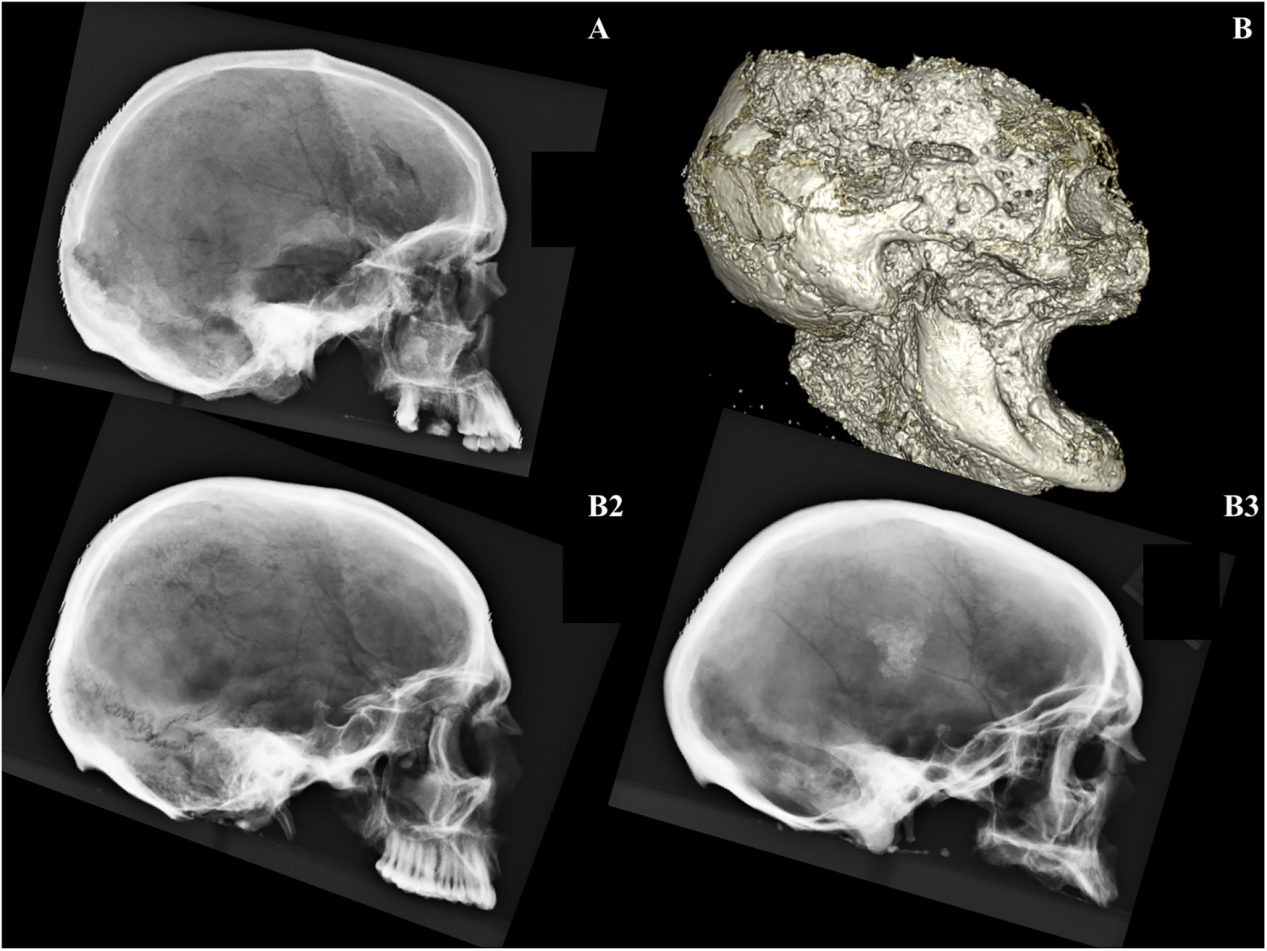
YNH4 lateral radiographs. YNH4 A has a damaged splanchnocranium. The fragile condition of the YNH4 B skull required a CT scan while still in matrix. Radiographs by GJC, TG and NAP

**Fig 9:**
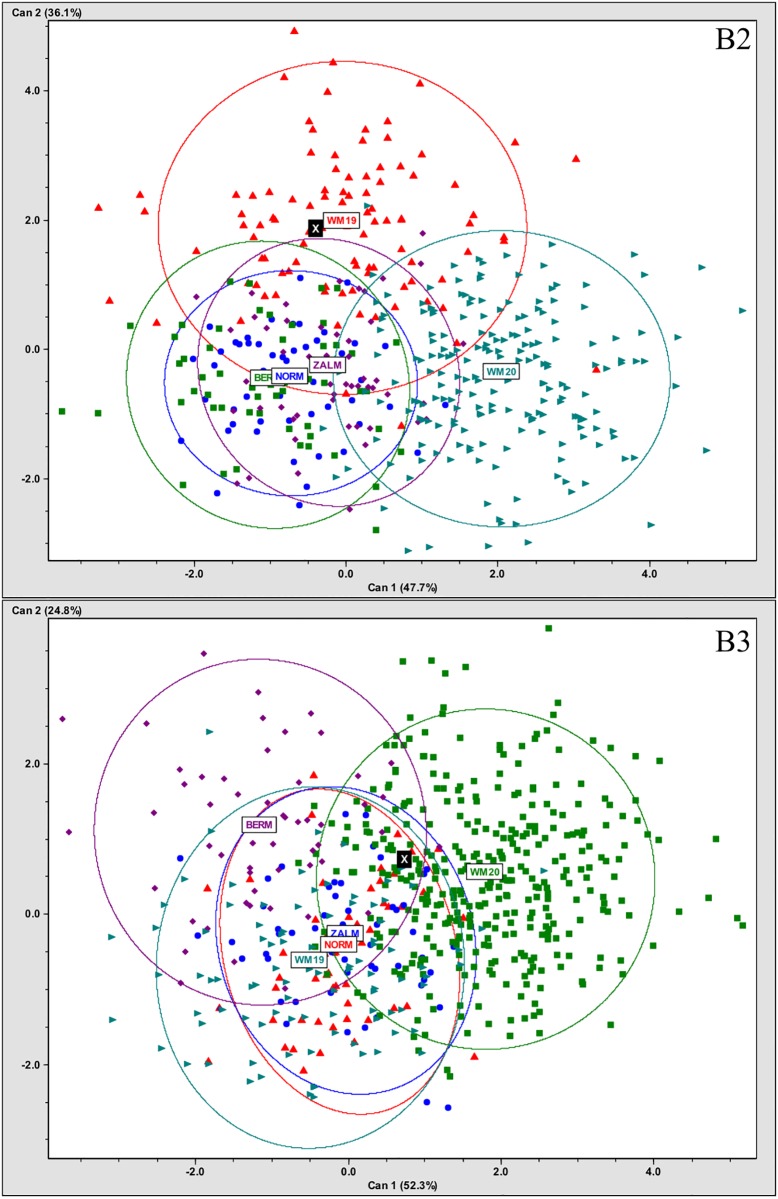
Ancestry estimations for YNH4 individual B2 (top) and B3 (bottom). “X” indicates YNH4 individual location. See S4 Table for data and abbreviations.

Fig 6 illustrates the elements recovered for each individual. All individuals exhibit skeletal markers of stress, disease, trauma, and occupation (Fig 6). All individuals are middle aged adults based on suture closure and bone histology review. Only two skulls (B2 and B3) were sufficiently intact for metric analyses (Figs 7 and 8; S2 Table). Metric and nonmetric assessment (S2 and S3 Tables) indicate European ancestry for both individuals, with B2 most similar to the 19^th^ century American sample, and B3 most similar to the Norwegian sample (Fig 9; S4 Table). Note that these results are affected by the limited number of available measurements, available comparative craniometric samples and questions regarding FORDISC’s accuracy and operating assumptions [107, 108].

**Fig 10:**
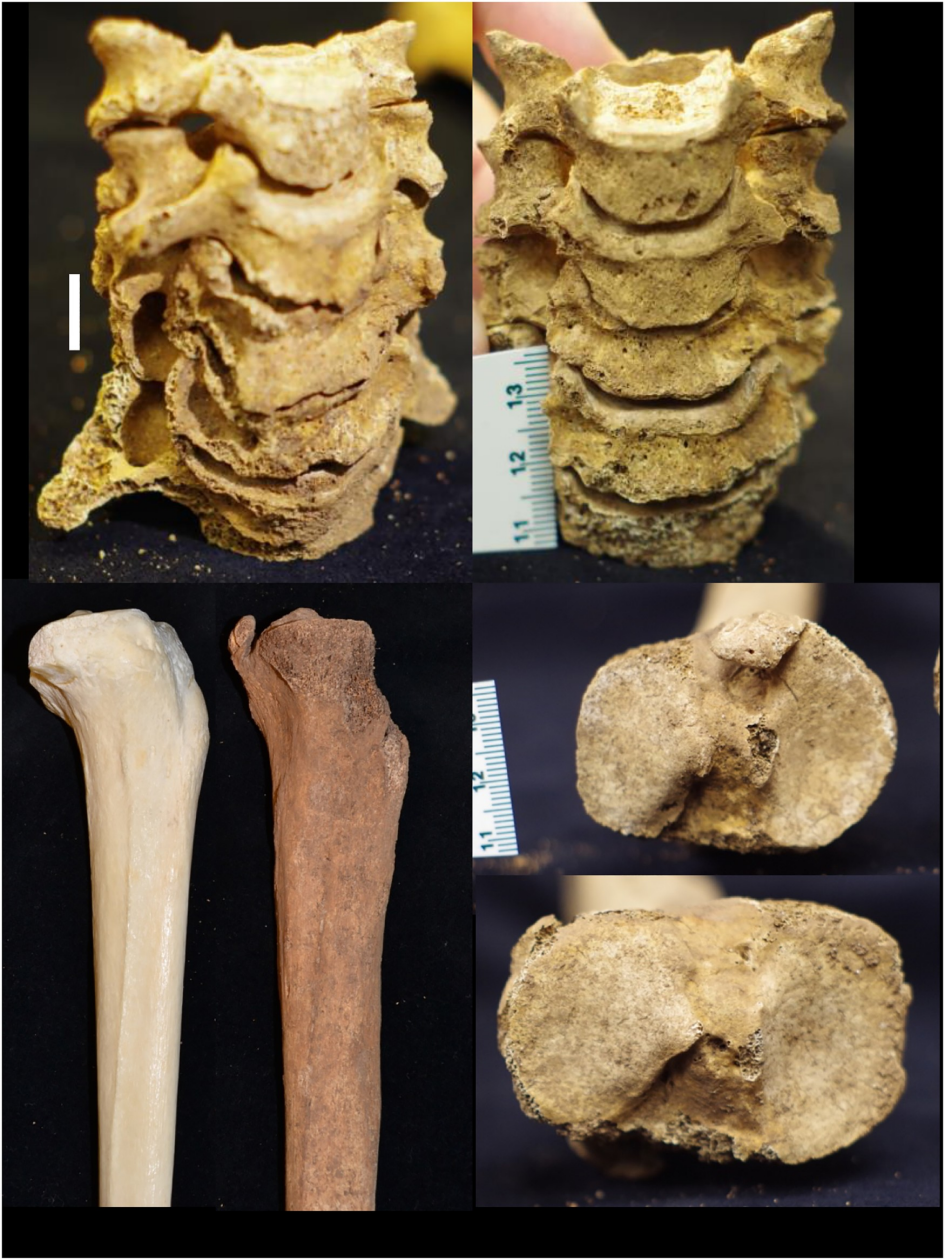
YNH4 postcranial pathologies. Top: YNH4 A (left) and B (right) cervical vertebrae enthesophytes. Bottom left: lateral view of YNH4 B proximal tibia (right) and a normal tibia (left). Bottom right: superior view of YNH4 B (top) and B2 (bottom) left proximal tibiae; See text for details.

Each individual presents musculoskeletal evidence of hard and/or repetitive labor, with joint disease and/or enthesopathies visible across multiple elements (Fig 10, S5 Table). There is variation in the structure and location of these markers—Individual A (female) shows marked degenerative arthritis of the cervical vertebrae, while B (also female) shows enthesophytes and bone spurs at both knee joints (Fig 10). The two males show multiple indicators of heavy labor, including rugose muscle markings, enthesophytes and vertebral markers of compression and stress (S5 Table). B3 is the oldest individual recovered and shows the most severe arthritic features, including bilateral eburnation of the scaphoid bones.

**Fig 11:**
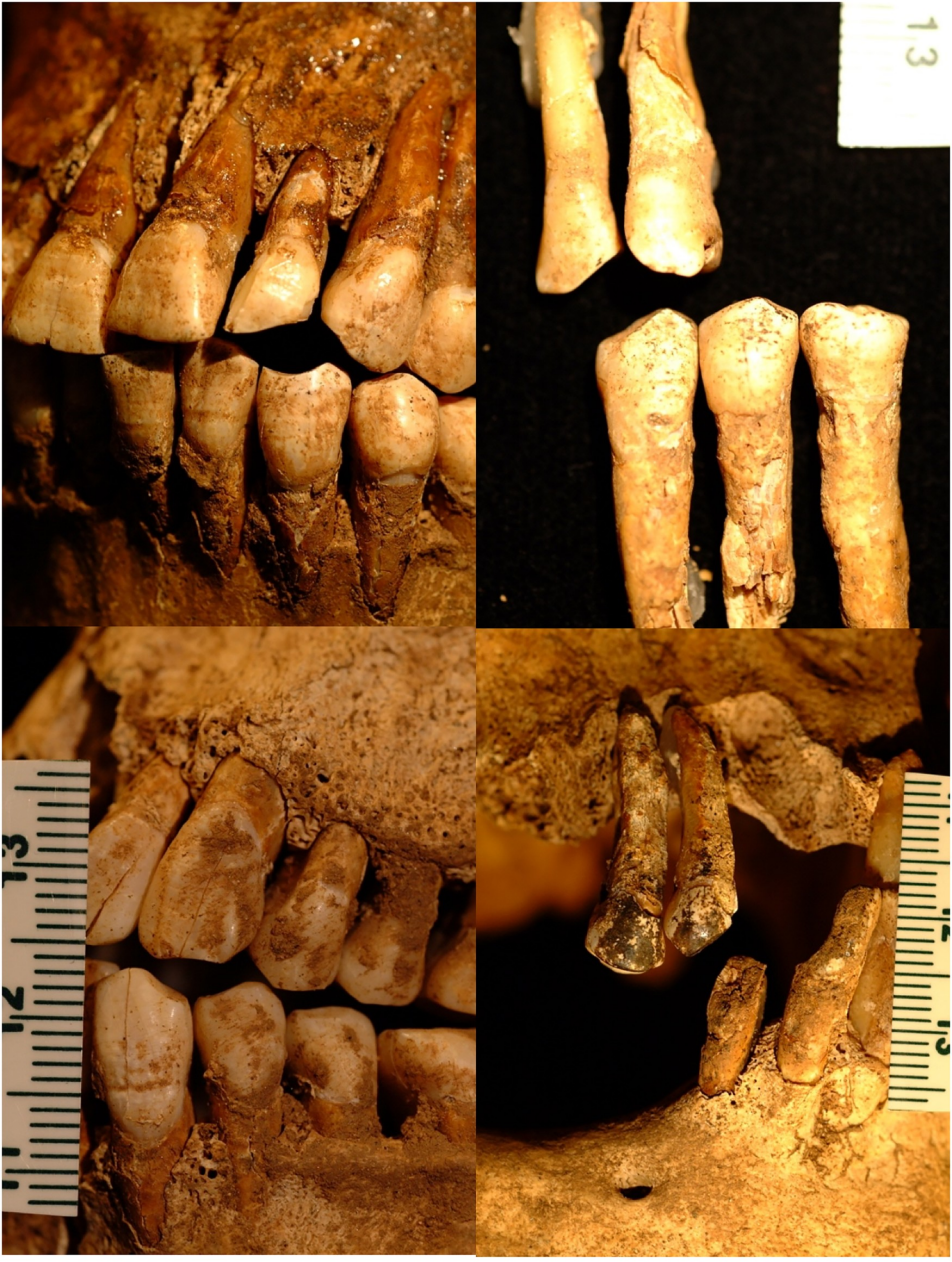
Dental wear resulting from habitual pipe smoking. Clockwise from top left: YNH4 A, B, B2, B3. Scale in mm.

All individuals present with dental disease, with older individuals B & B3 having the most severe indicators. All individuals also show asymmetric notches at the second incisor/canine margins, consistent with habitual ceramic pipe smoking (Fig 11) [109, 110].

**Fig 12:**
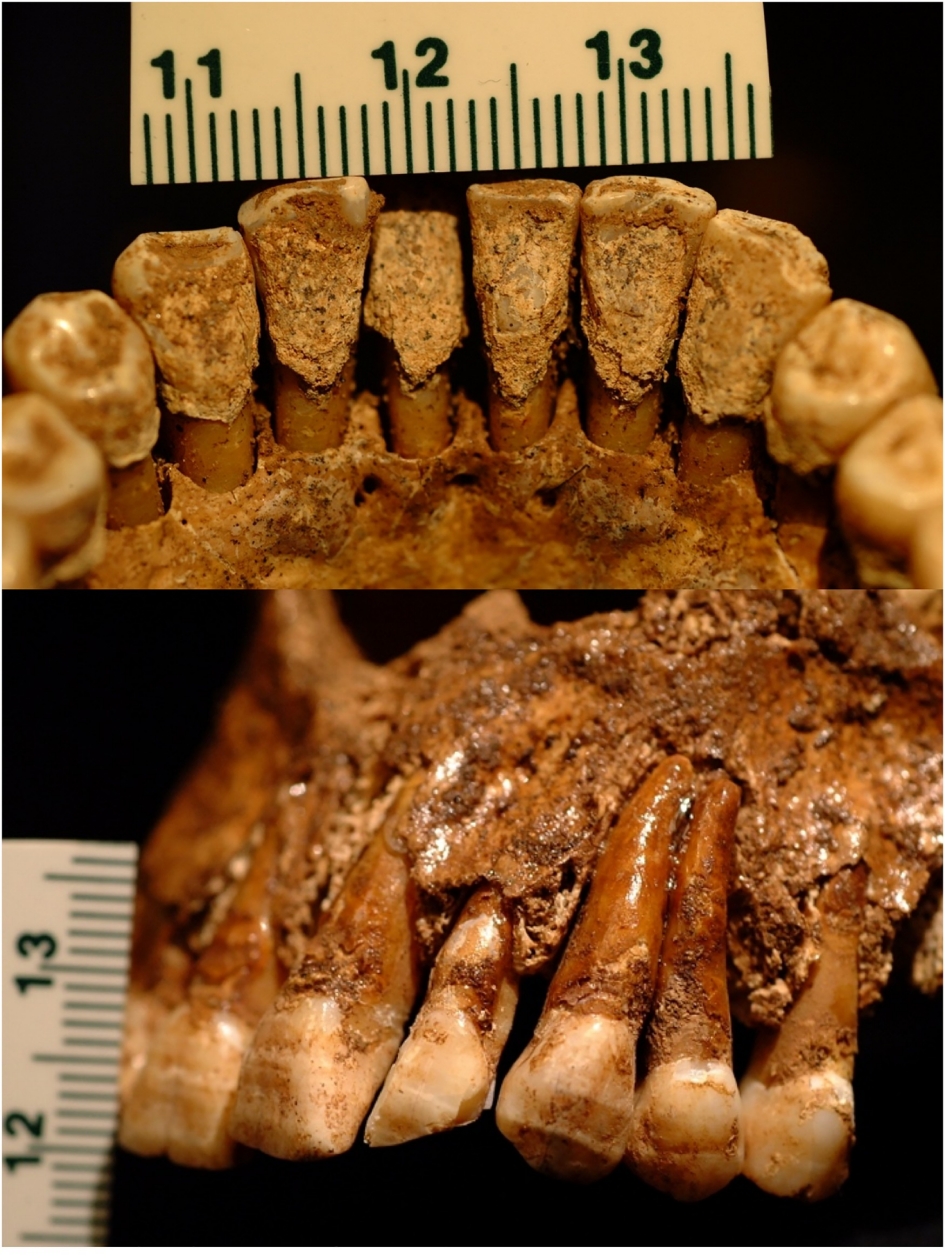
YNH4 dental pathologies. Top: dental calculus accretion on YN4 A anterior mandibular dentition. Bottom: serial linear enamel hypoplasias across YNH4 A maxillary incisors and premolars. See text for details.

Calculus is present on the anterior dentition of all individuals, presenting with annular distribution around the crown and highest thickness on the lingual aspect of the anterior dentition (Fig 12). All individuals exhibit severe periodontal disease and antemortem tooth loss; alveolar resorption in complete in all individuals except Individual B, who retains the apical aspects of alveoli for both upper and lower anterior dentition. Linear enamel hypoplasias show seriation and vary in severity across individuals (Figs 11 and 12). Enamel hypoplasias are located in the three- to four-year age range of crown formation [111], which may be associated with weaning, lack of adequate nutrition, immunological assaults, or any combination of these variables [9, 112–114]. Individual A presents the longest disruption (~0.8mm width measured on upper and lower incisor crowns) relative to the other individuals, who show narrower hypoplasias. The older individuals B and B3 have thick black residue on the lingual and buccal aspects of anterior teeth, suggesting occupational exposure to metals and/or tobacco staining (Fig 11, see below).

**Fig 13:**
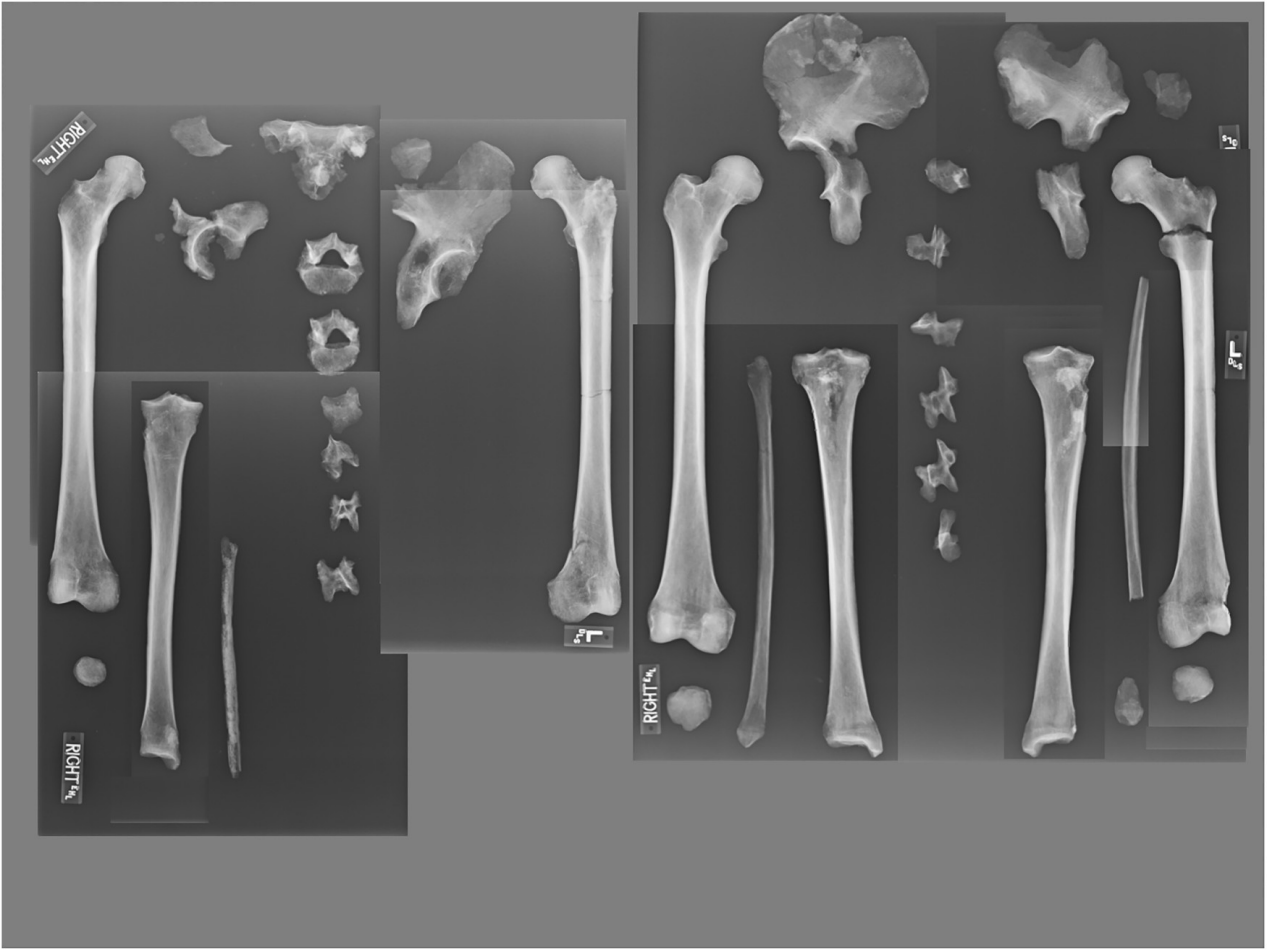
Comparison of YNH4 B (left) and B2 (right) lower limb radiographs. Note relatively higher translucence and lower bone density in B relative to B3. See text for details.

**Fig 14:**
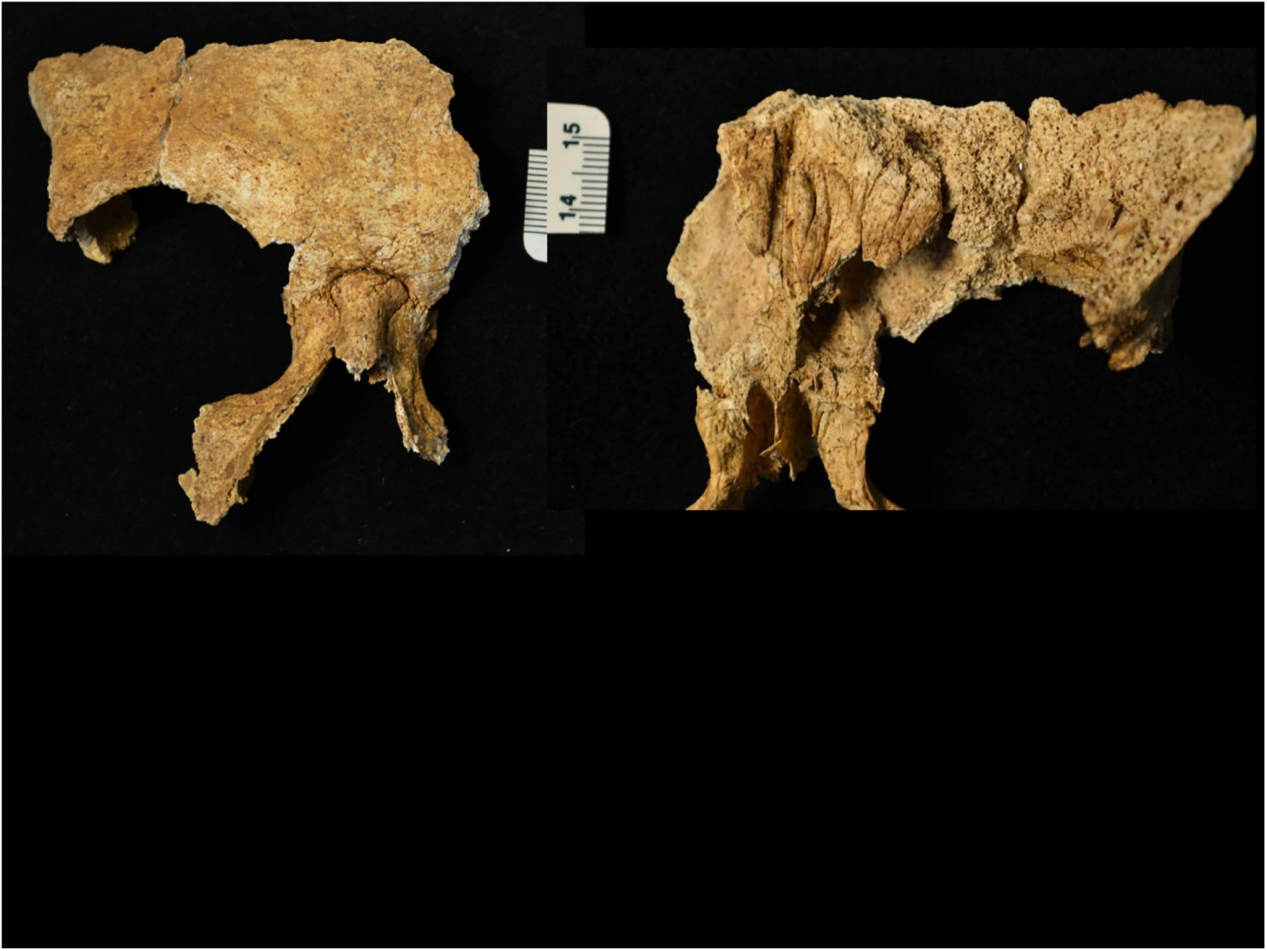
YNH4 B frontal external (left) and internal (right) surfaces, the latter showing evidence of hyperostosis frontalis interna.

**Fig 15:**
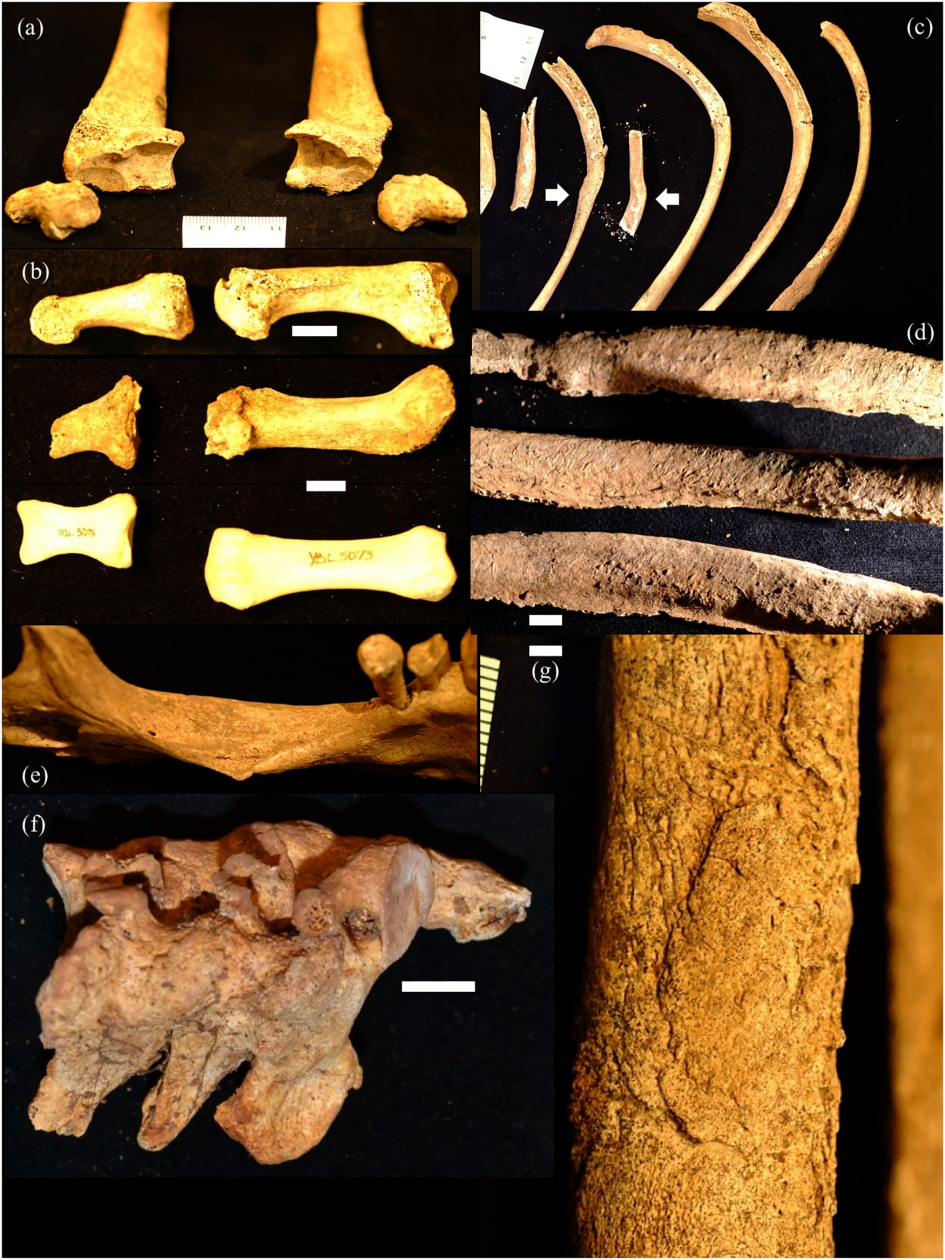
YNH4 B3 pathology markers. a) bilateral distal radii eburnation, b) gout indicators in the pedal and manual rays compared to normal ray (bottom), c) callous bone formation at rib fracture (arrow), d) remodeling of rib surfaces, e) left mandibular corpus antemortem fracture, f) fused C2-4 vertebrae, g) = distal tibia remodeling. Bar scale = 5mm.

Multiple indicators of chronic infection and other health issues are present across individuals. Individual B’s skeleton presents with marked osteomalacia (Fig 13) and hyperostosis frontalis interna (Fig 14), indicating menopause or chronic estrogen deficiency [115, 116]. Individual B3 has the most severe and chronic health issues—multiple ribs show remodeling and periosteal new bone medially (Fig 15). Hand and foot elements and joints show indicators of gout/pseudogout (Fig 15) [117–120]. The distal aspects of the left tibia and fibula show severe osteomyelitis and active bone remodeling (Fig 15).

**Fig 16:**
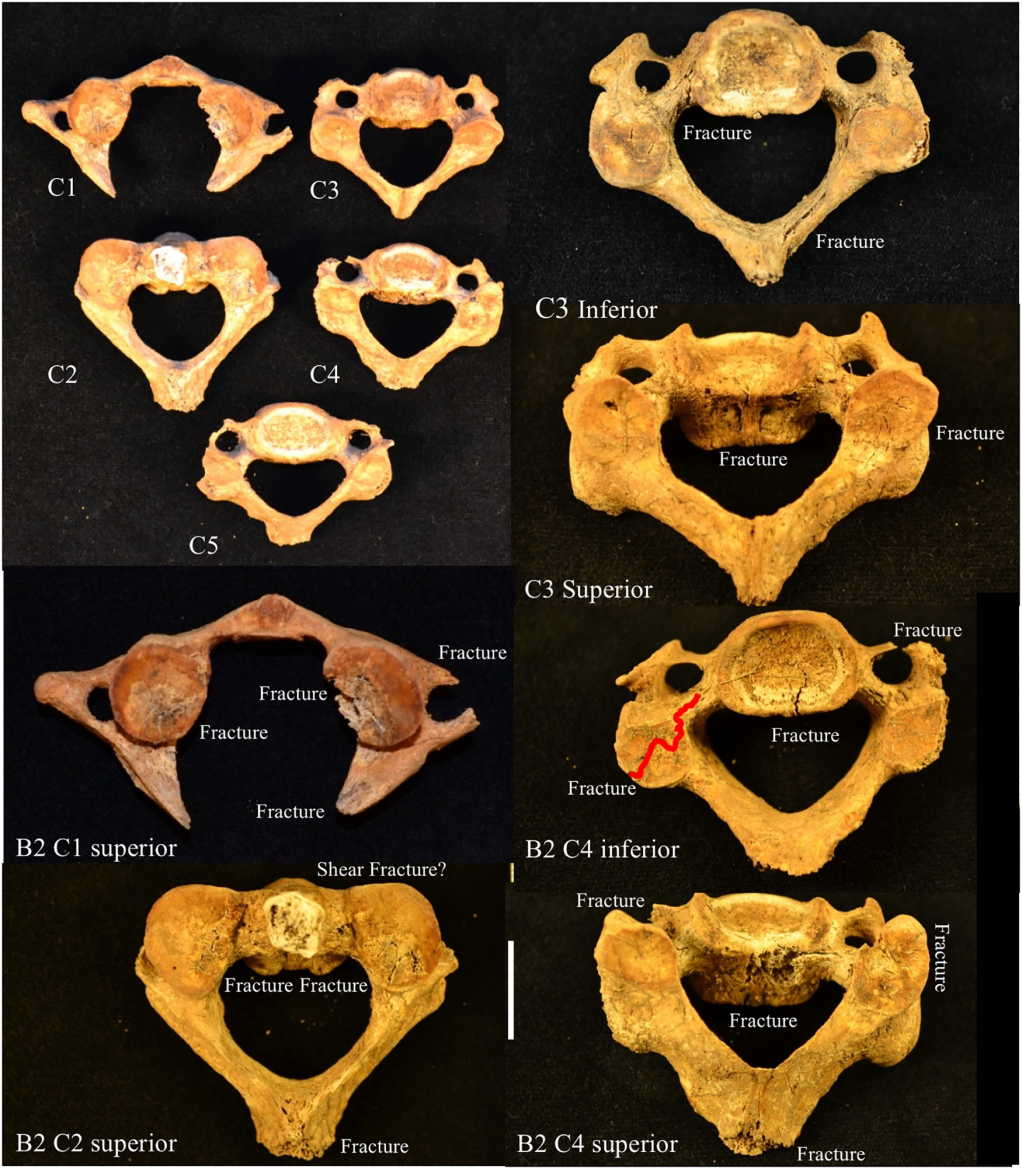
Indicators of perimortem trauma to the cervical vertebrae of YNH4 B2. Top left: overview of affected. Remaining images are detailed images of fracture patterns. See text for details.

Each male exhibits antemortem and perimortem trauma indicators respectively. Individual B3 has multiple antemortem trauma markers, including fused cervical vertebrae and proximally fractured ribs with marked callus bone formation (Fig 15). B3 also has a healed depressed cranial fracture (~3.0mm in diameter) and a healed fracture of the left mandibular corpus (Fig 15). Individual B2 shows perimortem injuries at neck, with fractures at both hyoid greater cornu, fracture of the C1 posterior neural arch, shearing of the C2 dens and torsion/compressive damage to the C3-C4 right superior and inferior articular facets (Fig 16). The pattern of damage appears consistent with hyperextension or torqueing of the neck, with fracturing of the C1 posterior arch and compression of the right C2-C4 articular facets.

*In toto*, all four individuals show shared cultural ecology and labor requirements, with chronic health issues inscribed on bones and teeth reflecting lower socioeconomic status and associated amplification of metabolic stressors.

### Genetic indicators of relatedness, phenotype and ancestry

Genetic data were preserved for all four individuals recovered, but nuclear data were not recovered from individual B2. Genetic data support osteological sex determination for the other individuals.

Individuals A, B and B3 do not show direct kinship, but the degree of autosomal similarity suggest that they are closely related at the population level, suggesting regional/local association (i.e. similar town/village origin). Individuals B and B3 show the strongest genetic similarity within the three individuals (Table 2). Hair and eye color are consistent with European ancestry. Individual B most likely had blue eyes, while individual B3 had brown eyes. Individual A eye color is intermediate between the latter two. Individuals A and B likely had dark (but not black) hair, while B3 had dark blond hair (Table 2).

**Table 2:** YNH4 genotype and phenotype characteristics.

Autosomal STR consensus genotypes								
Individual	Amelogenine	D13S317	D21S11	D18S51	TH01	D5S818	FGA	D3S1358
A	X/X	8/11	31.2/31.2	12/15	6/9	12/12	19/23	14/16
B	X/X	8/12	30/31	13/14	6/9.3	11/12	20/22	14/15
B3	X/Y	11/11	29/31.2	12/17	9/9.3	11/11	20/23	14/15
Individual	VWA	D8S1179	D7	D9S1120	D16S539	D2S1338	D19S433	
A	15/16	14/19	11/11	12/15	9/13	n.d.	13/15	
B	17/17	13/16	10/10	15/15	9/13	20/24	15/15	
B3	16/17	13/17	8/10	16/18	11/12	20/20	13/14	
Mitochondrial HVR1 Region sequencing results								
Individual	Sequenced Positions		Nucleotide Positions differing from rCRS					Haplogroup
A	16024–16400		16069T, 16126C, 16193T, 16278T, 16291T, 16304C					J2B1a
B	16024–16400		16069T, 16126C, 16193T, 16278T					J2B1a
B2	16024–16400		16294T, 16296T, 16304C					T
B3	16024–16400		16311C					H
Y-chromosomal STR consensus genotype (minimal haplotype) for the male individual B3								
Individual	DYS391	DYS389 I	DYS389 II	DYS19	DYS392	DYS393	DYS390	DYS385
B3	10	13	-	-	13	13	24	11, 15
Genotyping results for the HirisPlex Assay								
	SNPs							
Individual	rs1800407-R	rs16891982-F	rs12203592-R	rs12913832-R	rs1393350-R	rs12896399-F	rs1805005 f	rs1805007 f
A	C/T	G/G	A/A	C/T	C/C	G/G	G/G	C/C
B	-	-	-	-	-	-	-	-
B2	-	-	-	-	C/C	G/G	G/G	C/C
B3	C/C	G/G	G/A	C/T	C/T	G/G	G/T	C/C
	SNPs				Predicted Eye color (IrisPlex)			
Individual	rs1805008 r	rs28777 r	rs12913832 r	rs16891982 r	Blue	Intermediate	Brown	Hair color
A	G/G	T/T	C/T	C/C	0.3074	0.4557	0.2369	dark
B	-	-	-	-	n.d.	n.d.	n.d.	dark
B2	G/G	T/T	C/T	C/C	n.d.	n.d.	n.d.	n.d.
B3	G/G	T/T	C/T	C/C	0.1965	0.2447	0.5588	dark blond

Mitochondrial sequence data of the Hyper Variable Region I and II were used to determine the specific mitochondrial haplotypes. Individuals B2 and B3 belong to haplotypes T and H (Table 2), commonly found in European populations and North American individuals of European ancestry [121]. Individuals A & B belong to mitochondrial haplogroup J2B1a, which is relatively rare in modern Central Europe. This haplotype is most common in Southern/South-Eastern Europe and the Levant, and less common in Ireland [122, 123].

Overall, the autosomal STR allele profiles are most consistent with populations from Eastern and South-Eastern Europe, specifically Poland and Romania [124–127]. We were able to determine the Y chromosomal haplotype for individual B3. The nine STR minimal haplotype is most closely associated with males from Southern Italy, Romania and the United Kingdom [86]. In all cases, no allelic markers were consistent with population genetics reported from Ireland.

### Chemical indicators of occupation, diet and geographic origin

**Fig 17:**
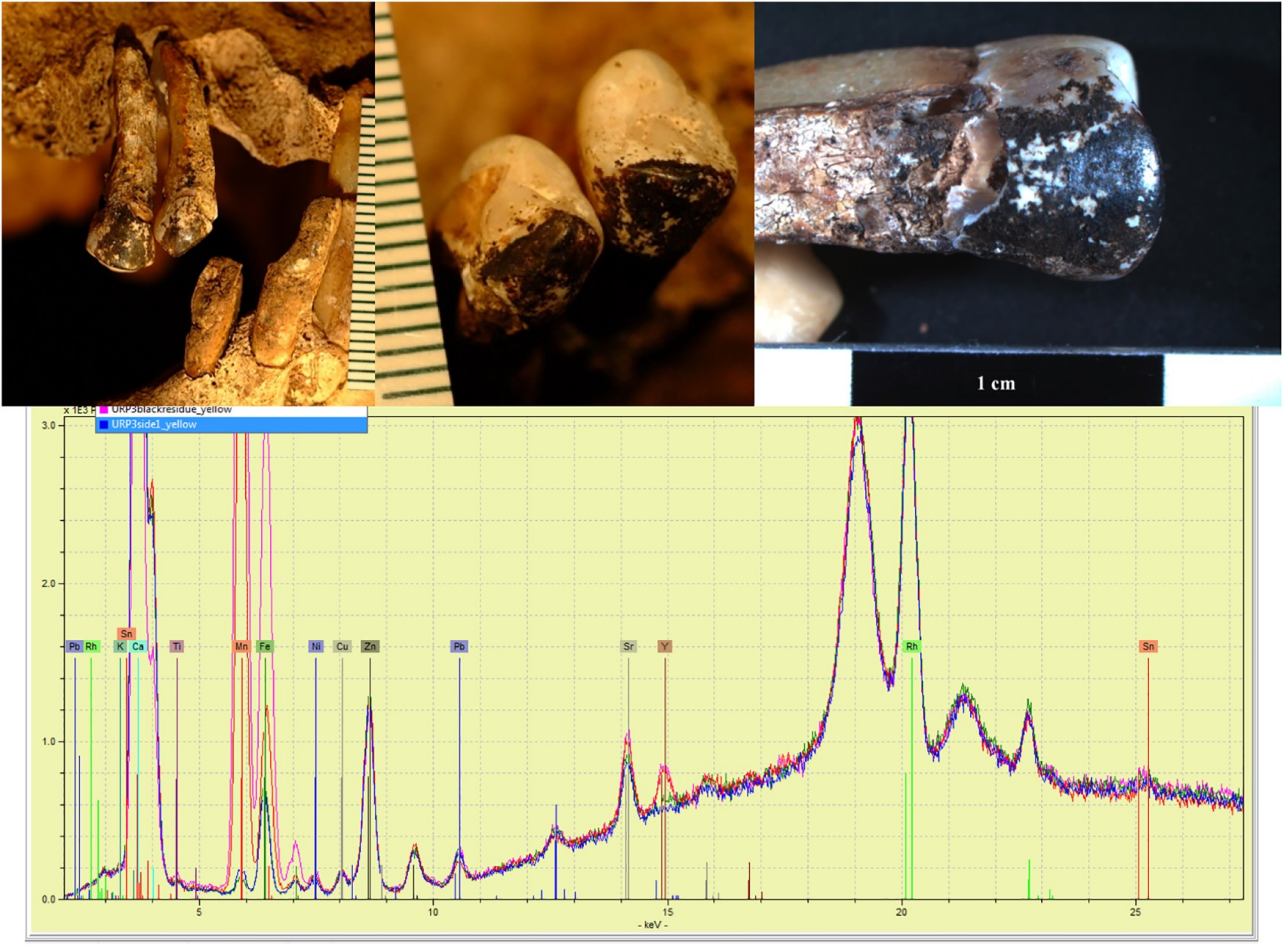
YNH4 tooth staining. Top left, center: staining and residue on YNH4 B3 RP^3-4^. Top right: close up of RP^4^. Bottom: Bruker pXRF analysis. See text for details.

**Fig 18:**
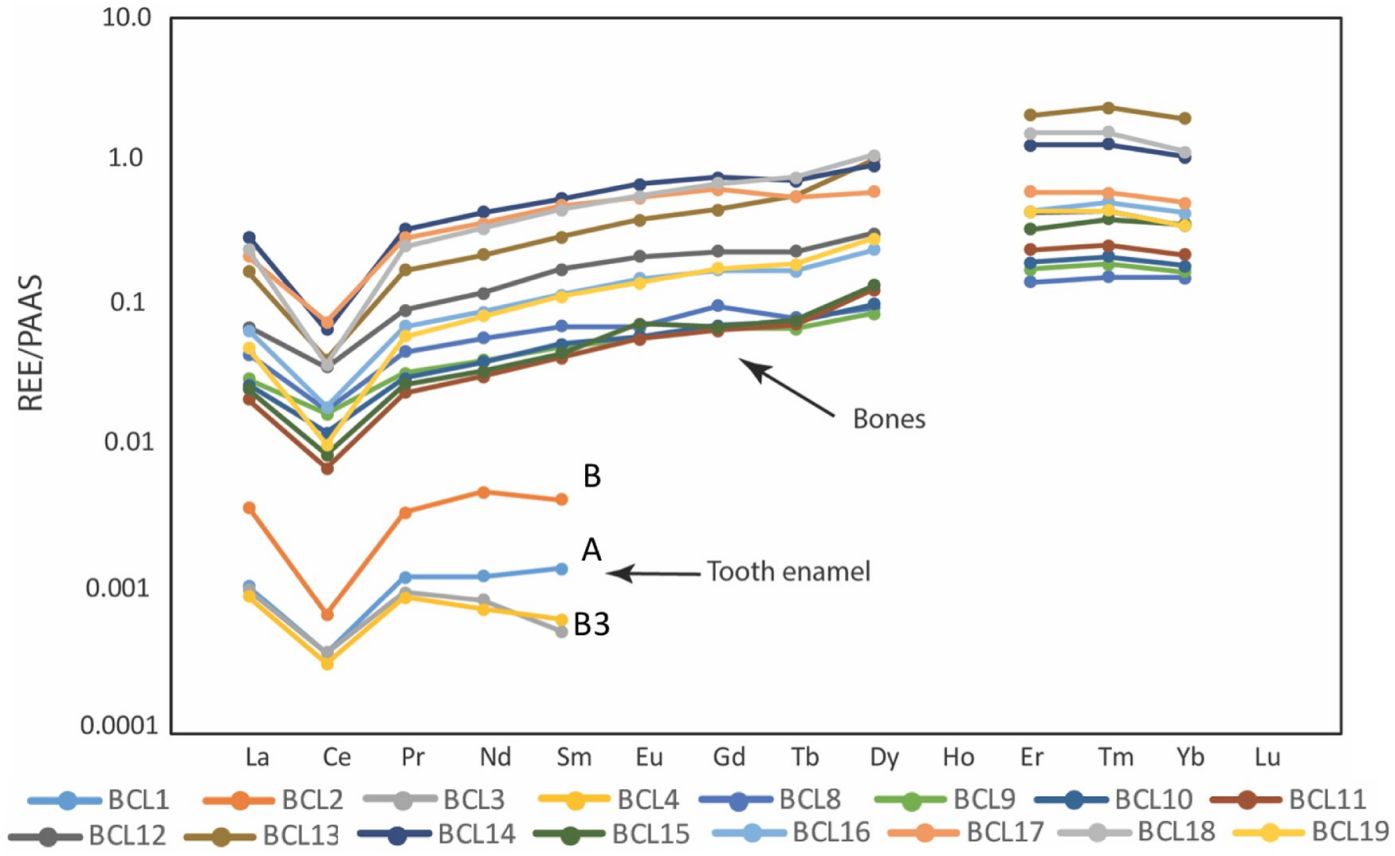
Normalized REE data for tooth enamel (BCL1-BCL4) and bone (BCL8-BCL19). Abbreviations in Table 3. Note the highly elevated REE in bone compared to enamel, indicating postmortem diagenetic change in all of the bone samples.

Multiple individuals show thick black residue on the labial aspect of their anterior dentition (Fig 17). Elemental analysis via Bruker xPRF indicates relatively high concentrations of manganese, iron and yttrium, indicating occupational exposure (i.e., using mouth to hold/manipulate metal objects), tobacco secondary compounds (tooth staining/residue from constant pipe smoking) and/or postmortem precipitation of Fe-Mn oxyhydroxides. Direct evidence for marked diagenetic change is indicated by high concentrations of rare earth elements (REE) in the bone samples, limiting bone assessment of adult-aged residency/mobility via elemental analysis and/or Sr and Pb isotopes (Table 3, Fig 18). As tooth enamel is more resistant to diagenesis, all YNH4 enamel samples show low REE concentrations (Fig 18). While Individual B’s enamel shows slight elevation of Vanadium (V), Lanthanum (La), and Neodymium (Nd) concentrations above Maximum Threshold Concentrations (0.11, 0.1, and 0.058 respectively) the Sr-Pb-O-C isotope values are within the range expected for *in vivo* recovery [128]. For all YNH4 individuals, we focus analysis on tooth enamel isotopes to reconstruct diet and childhood place of residence.

**Table 3:** Trace element data (ppm) for YNH4 tooth enamel and bone samples.

	Tooth enamel				Rib bone samples											
Individual	A	B	B3 M1	B3 M2	A			B			B2			B3		
Sample #	BCL1	BCL2	BCL3	BCL4	BCL8	BCL9	BCL10	BCL11	BCL12	BCL13	BCL14	BCL15	BCL16	BCL17	BCL18	BCL19
Mg	3101	2835	3386	3440	1084	1010	1071	995	1128	1158	1170	983	1079	1244	1028	960
Sc	0.03	0.03	0.02	0.02	0.22	0.25	0.21	0.22	0.56	1.32	0.93	0.31	0.42	0.54	0.71	0.33
V	0.08	0.62	*	*	8.33	11.79	23.06	4.08	27.90	6.70	5.10	4.23	3.77	9.96	4.64	4.26
Cr	*	0.01	0.01	*	2.83	3.41	4.99	1.44	7.59	2.75	2.57	2.35	1.45	0.82	1.53	1.10
Co	0.01	0.02	0.01	0.02	0.36	0.32	0.31	0.26	1.06	1.75	1.40	0.84	0.98	0.72	0.70	0.78
Ni	0.30	0.36	0.39	1.50	2.48	2.58	2.06	3.18	3.30	8.51	6.22	4.26	6.64	5.12	3.96	5.13
Cu	0.61	0.73	0.57	0.70	127	158	21	17	37	25	71	23	55	39	85	96
Zn	87	172	107	88	526	535	207	291	191	854	1696	457	1072	331	1193	559
Sr	31	67	65	71	268	279	282	312	358	398	459	521	453	426	476	435
Y	0.03	0.28	0.01	0.01	3.10	2.54	3.06	3.81	8.15	34.49	28.02	4.27	7.15	16.25	32.06	8.54
Cd	0.008	0.231	0.012	0.005	2.82	3.08	2.02	3.22	2.43	11.34	42.53	8.77	35.29	1.57	41.50	19.54
Ba	10	11	0.31	-0.02	104	113	123	160	175	233	267	211	207	51	178	148
La	0.0403	0.1441	0.0381	0.0345	1.691	1.137	1.034	0.823	2.615	6.414	11.192	0.969	2.489	8.210	9.086	1.865
Ce	0.0294	0.0542	0.0293	0.0246	1.467	1.370	1.008	0.571	2.902	3.300	5.345	0.712	1.521	6.046	3.034	0.832
Pr	0.0110	0.0307	0.0086	0.0078	0.409	0.291	0.271	0.214	0.803	1.539	2.939	0.243	0.624	2.587	2.234	0.522
Nd	0.0400	0.1548	0.0275	0.0236	1.857	1.301	1.265	0.995	3.830	7.085	13.981	1.081	2.834	11.773	10.920	2.643
Sm	0.0079	0.0242	0.0029	0.0035	0.394	0.283	0.293	0.237	0.980	1.653	3.077	0.253	0.656	2.755	2.554	0.632
Eu	*	*	*	*	0.076	0.064	0.066	0.063	0.237	0.429	0.759	0.081	0.166	0.614	0.625	0.156
Gd	*	*	*	*	0.456	0.319	0.332	0.307	1.088	2.143	3.597	0.324	0.806	2.958	3.267	0.826
Tb	*	*	*	*	0.062	0.052	0.058	0.055	0.181	0.437	0.562	0.060	0.132	0.433	0.592	0.147
Dy	*	*	*	*	0.419	0.377	0.444	0.553	1.383	4.514	4.120	0.598	1.059	2.674	4.830	1.269
Er	*	*	*	*	0.410	0.500	0.557	0.685	1.258	5.935	3.698	0.959	1.268	1.751	4.489	1.276
Tm	*	*	*	*	0.063	0.077	0.087	0.105	0.183	0.962	0.534	0.159	0.211	0.245	0.643	0.183
Yb	*	*	*	*	0.426	0.470	0.517	0.621	0.995	5.590	2.997	1.013	1.213	1.426	3.255	0.984
Pb	2	1	5	1	93	88	67	14	108	68	60	19	39	171	224	186
Th	*	*	*	*	0.06	0.07	0.04	0.03	0.11	0.09	0.18	0.04	0.05	0.11	0.07	0.04
U	0.007	0.088	*	*	1.20	2.53	2.95	0.70	9.44	2.67	1.10	0.27	0.47	0.14	1.33	0.27

* = below detectable levels. See text for details.

**Fig 19:**
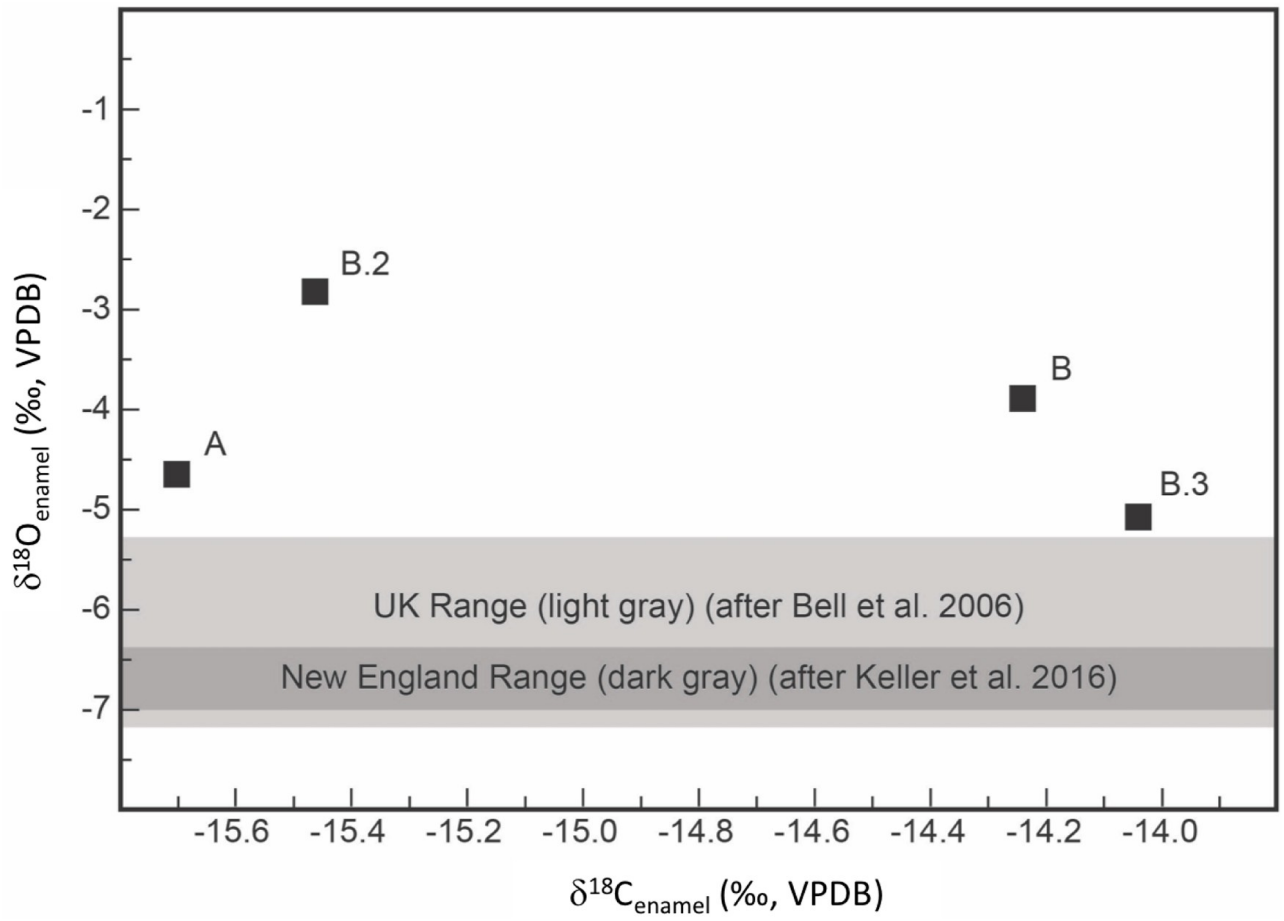
YNH4 tooth oxygen and carbon isotope values, and published geographic ranges.

The YNH4 average carbon isotope value (δ^13^C) is -15‰, indicating a C3-based terrestrial diet for all four individuals (Fig 19). This is consistent with historical evidence for European-based diets and suggests minimal consumption of C4-based food products (i.e., millet, corn, sugar cane) during childhood [68].

Sr concentration in the four individuals assayed range from 31 to 84 ppm, which is on the low end of Sr concentrations measured in modern and archaeological human enamel [128]. Measured Sr isotope ratios in the enamel (^87^Sr/^86^Sr = 0.70888 to 0.71158; Table 4) fall within the possible Sr isotope range for archaeological tooth enamel in Britain [129]. However, this Sr isotope range is not unique to Britain—Sr isotope ratios in human enamel from individuals born during the 20^th^ century show considerable overlap between Europe and the United States, limiting the value of strontium for distinguishing geographic origin in this case [130].

**Table 4:** YNH4 tooth enamel isotopic data.

YNH4 Individual	Tooth	UF ID#	δ ^13^C_en_ (‰, vs VPDB)	δ^18^O_en_ (‰, vs VPDB)	^87^Sr/^86^Sr	Sr conc. (ppm)	^208^Pb/^204^Pb	^207^Pb/^204^Pb	^206^Pb/^204^Pb
A	RM^1^	BCL1	-15.7	-4.6	0.711580	55	38.2449	15.5954	18.3593
B	LC_1_	BCL2	-14.2	-3.9	0.708881	71	38.4719	15.6243	18.5258
B2	LM^2^	BCL20	-14.0	-5.1	0.709312	31	38.8399	15.6700	18.9273
B3	RM^1^	BCL3	-15.5	-2.8	0.710922	84	38.3015	15.6127	18.3547
B3	RM^2^	BCL4	-15.4	-3.5	0.710766	83	38.3987	15.6291	18.4316

**Fig 20:**
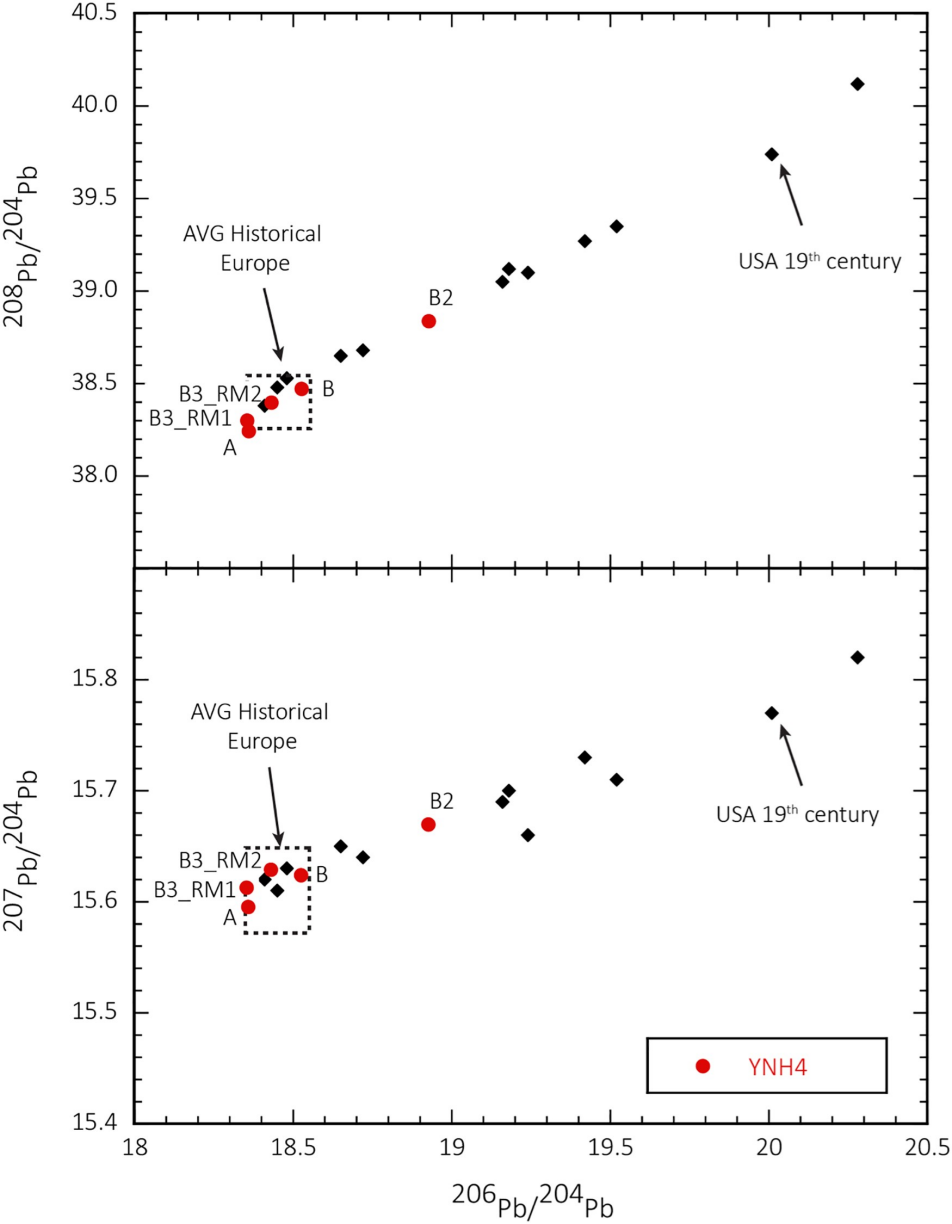
Lead isotope comparison between YNH4 individuals and average historical Europe and 19th century USA lead patterns. Note that all samples with exception of B2 plot within the average Europe field, consistent with inferred European origin of these individuals. B2 plots outside the European field. See text for details. Figure modified from Kamenov and Gulson [131].

In contrast to Sr isotopes, Pb isotopes provide greater discrimination of European versus United States origin [131]. Enamel samples from all four individuals show elevated Pb levels indicating historic anthropogenic exposure [128]. Tooth enamel Pb ratios for all YNH4 individuals except individual B2 plot within the historical European Pb isotope space (Fig 20, Table 4), indicating childhood residence and development outside of the USA. The Pb isotope signal for Individual B2 associates closely with 19^th^ century individuals buried in Colorado, USA, but note that those individuals are likely recent European immigrants themselves [132]. Overall, Individual B2’s LM^2^ lead profile indicates a different immigration and/or residency history than the other three YNH4 individuals, or this individual may have been exposed to a lead source that is distinct from the available published anthropogenic lead values.

Oxygen isotope data of the YNH4 sample are presented in Fig 19. New England human tooth δ^18^O values range between -7‰ to -6.4‰, with a single sample from Connecticut having a value of -6.74‰ [133]. In contrast, all of the YNH4 individuals present with more enriched δ^18^O values, ranging from -2.8 to -5.1 (Table 4), indicating a different developmental history relative to New Englanders. These values also fall outside those reported for Great Britain (-5.3 to -7.1) [134]. Individual B2 enamel δ^18^O value (-5.1%) is consistent with data reported from Cumbria (-5.3), a site close to Dublin[134]. However, this δ^18^O value for tooth enamel falls within the range of other European countries as well [29, 130]. While genetic data suggest Polish or Romanian origins for Individuals A, B, and B3, δ^18^O values are inconsistent with those recorded for this region. Polish data values (n = 2) are -6.6‰ and -6.9‰ respectively [29], and modern Bulgarians (immediately south of Romania) have δ^18^O values around -6‰ [130]. Individuals A, B and B3 exhibit enamel with higher δ^18^O values (Table 4) that are more consistent with southern European/Italian residence during molar tooth development. It is possible that the individuals are of Southeastern/Southern European descent and subsequently moved and grew up somewhere at or near the Mediterranean coast.

#### Dental calculus indicators

Analysis of the amplified 16S rRNA gene V3 sequences confirms preservation of oral microbiome DNA in the YNH4 dental calculus samples (S6 Table). The majority (~60–90%) of taxonomically assigned sequences belong to genera known to be present in the human oral cavity [135, 136]. All four samples also exhibit higher than expected frequencies of the oral archaeon *Methanobrevibacter* and members of the candidate phylum Sachharibacterium (TM7), a known analytical artifact that has been previously reported for ancient oral microbiome samples that contain low levels of exogenous contamination but highly fragmented DNA [92]. The genera *Porphyromonas*, *Treponema*, and *Tannerella*, which contain members associated with periodontal disease, were each found at low abundance (0.01–0.8%) in three of the four individuals (A, B, B2), while only the latter two genera were observed in the dental calculus of individual B3 (S6 Table).

Because it has been previously shown that *Mycobacterium tuberculosis* infection can be identified by genetic analysis of dental plaque [137], we analyzed the dental calculus (calcified dental plaque) of the four YNH4 individuals for genetic sequences specific to this organism. *Mycobacterium* sequences were present in the dental calculus of three of the four individuals (A, B, and B3), but at very low levels (0.01% to 0.08%) (S6 Table). Further analysis revealed that these *Mycobacterium* sequences are inconsistent with *Mycobacterium tuberculosis* complex strains, and likely originate from common soil mycobacteria. These results therefore do not support a diagnosis of tuberculosis, but they also cannot exclude the possibility that these individuals were infected with tuberculosis.

## Discussion

Following discovery, our initial assumptions about the Christ Church cemetery suggested that it contained the remains of Irish and Irish-American parishioners. Macromorphoscopic analyses of the YNH4 skeletons provided concordant data, and the presence of a single rosary component connects these individuals to available historic records. Their location (stacked near the edge of the cemetery fence line) suggest that they were interred at a time when space was a premium, during a period of epidemic and multiple interments, or due to other cultural factors. As craniometric data alone cannot distinguish related populations [138, 139], a narrow bioarchaeological investigation would have misidentified these individuals as Irish immigrants. Multidisciplinary analyses upended these initial assumptions. While European individual and population-level variation and gene flow do not support assortative mating [140, 141] (but see Domingue et al. [142] for USA characteristics), genetic and isotopic data indicate that three of the YNH4 individuals are not from Ireland or any other part of the United Kingdom, while one has a more ambiguous history.

The early to mid-19^th^ century was a period of political unrest and upheaval, economic disparity and epidemics across multiple European nations [143–146]. As the immigrant diaspora into the United States and Connecticut intensified in scale and scope [147, 148], political, social and economic biases led to immigrants crowding into urban tenements or slums [149–151]. Like most New England cities, New Haven area immigrants mostly worked as laborers, working on laying the Farmington Canal, shipbuilding and working in garment factories [152–154].

All four skeletons show indicators of musculoskeletal, infectious disease and addictive substance (i.e., tobacco) stressors [155]. While debate persists on the roles of senescence, etiology and coding standards on entheseal pathologies [156–158], male individuals B2 and B3 show rugosity and arthritic changes likely associated with manual labor [11, 159–161]. While the skeletons of the two women are incomplete, we posit that the elements present indicate gendered labor biomarkers [162, 163]. Individual A shows enthesopathies and muscle insertion rugosity in the upper body. Individual B shows skeletal markers of reduced estradiols and associated osteomalacia, and tendon ossification and inflammation indicators are present at the knee joint [164]. We suggest that both of these women may have been employed in the garment industry or some other repetitive labor regime. During this time period, garment work was/is largely performed by immigrant women [153, 165]. The combination of a long workday (especially in the 19^th^ century), prolonged postural strain and repetitive work generate multiple joint stressors including neck, back and knee strain, leading to musculoskeletal pathologies [166–168].

Skeletal markers of chronic infection (ribs and lower limb) are preserved in individual B3 and dental disease/enamel hypoplastic indicators are present across all dental samples. These data further support the hypothesis that these individuals faced metabolic and immunological stresses throughout development and adulthood. Infectious disease epidemics and high mortality are clear in the Christ Church burial records, with typhus and dysentery deaths showing temporal pulses likely associated with seasonality and crowded living conditions (S1 Table). While tuberculosis (labeled “Phthisis”) was among the most common causes of death for the Christ Church parishioners, multiple studies describe variation in expression of tuberculosis infection across genetic and skeletal samples [44, 169, 170]. Our study is concordant with these prior studies—while skeletal markers such as rib lesions and osteomyelitic changes are present, *M*. *tuberculosis* was not recovered in dental calculus (S6 Table). The lack of molecular evidence does not falsify the possibility of tubercular infection, but this case reiterates the difficulty of connecting epidemiological and bioarchaeological data [171].

### New Haven’s “The Hill” neighborhood, Christ Church Cemetery demographics and ethnoreligious plasticity for early 19^th^ century New Haven immigrants

Northeastern USA cities are well-known for overcrowding lower-income immigrants into slums and tenements [172]. Crowded housing and low resource quality/availability, combined with poor hygiene and lack of adequate medical care led to widespread infectious disease and death within poor urban neighborhoods [173–179]. Within 19^th^ century New Haven, the neighborhood surrounding Christ Church was known as “The Hill”–it was (and continues to be) an epicenter for recent immigrant settlement [180–186]. New Haven residents’ life expectancy in the 1830s (46 years) was lower than for rural town residents (51 years), but longer than those in larger cities such as New York (35.9 years) [179, 187]. For many urban 19^th^ century churches, cemetery size and plot availability led to chaotic burial distribution—stacking of burials in urban cemeteries is commonly reported [188–192].

Demographic data for the Christ Church cemetery show that infectious disease was the most commonly recorded cause of death. Observed sex variation in infectious disease mortality, reproductive biology and potentially lethal/addictive behaviors are consistent with available data on human life history data [193, 194]. The Christ Church cemetery’s demographic pattern is largely consistent with 19^th^ century morbity and mortality data associated with infectious disease prevalence [195], urban living [155, 174, 176], childbirth complications [196–199] and the epidemiological transition during industrialization of the United States [179, 200–202]. The skeletal characteristics of the four Christ Church burials and the church’s demographic profile are concordant with other bioarchaeological investigations of structural violence [17, 19]. Skeletons representing the remains of marginalized populations due to ethnicity, migration, gender or other factors show similar patterns of dental pathologies, lesions, trauma and occupational health issues[42, 43, 57, 203–207]. We posit that socioeconomic, religious and geographic (i.e., the Hill neighborhood) inequalities are implicated in the condition of the four individuals as people and for the Christ Church community as a whole [208–210]. Compiling and examining individual, local and regional mortuary data sources associated with skeletal remains reiterates the value of multidisciplinary efforts and illuminates the intersection of structural violence, health, mortality and bioarchaeology [57, 211–214].

The Christ Church cemetery records indicate severe infant/child mortality and lower life expectancy across all age classes (Fig 4a and 4b). These data are most consistent with New York City’s Marble Cemetery, which housed city dwellers facing marked socioeconomic stratification and infectious disease risk (post-1838, wealthy New Yorkers sought interment elsewhere) [215]. Christ Church cemetery demography also mimics data for an 18^th^ century New Orleans slave cemetery, a population suffering from severe structural violence [99]. We offer two possible explanations for this pattern—the Christ Church parishioners faced similar pressures as these populations, including syndemic infectious disease risk and structural violence pressures [216, 217]. Alternatively, the observed mortality pattern and lower life expectancy are artificial, reflecting the opening of St. Bernard’s cemetery in 1854 –the older Christ Church parishioners would have been interred there and thus generating the lower survivorship curve of the Christ Church burials. Further review of burial records across the city and state may provide further support or falsification of these hypotheses.

The Christ Church burials are characterized by a relatively homogeneous population, namely individuals of Irish descent and/or Gaelic/Anglicized names (S1 Table). Genetic and isotopic data falsify the hypothesis that three YNH4 individuals (A, B and B3) are of Irish or Irish-American ancestry. Available genetic and isotopic indicators suggest that these individuals were adult (not child) immigrants to the United States/New Haven, and they share southern or southeastern European origin and/or descent. As described above, immigrants were critical in the developing American industrial complex, but immigrants from this region are underrepresented in United States census records during the 1830–1850 period.

**Fig 21:**
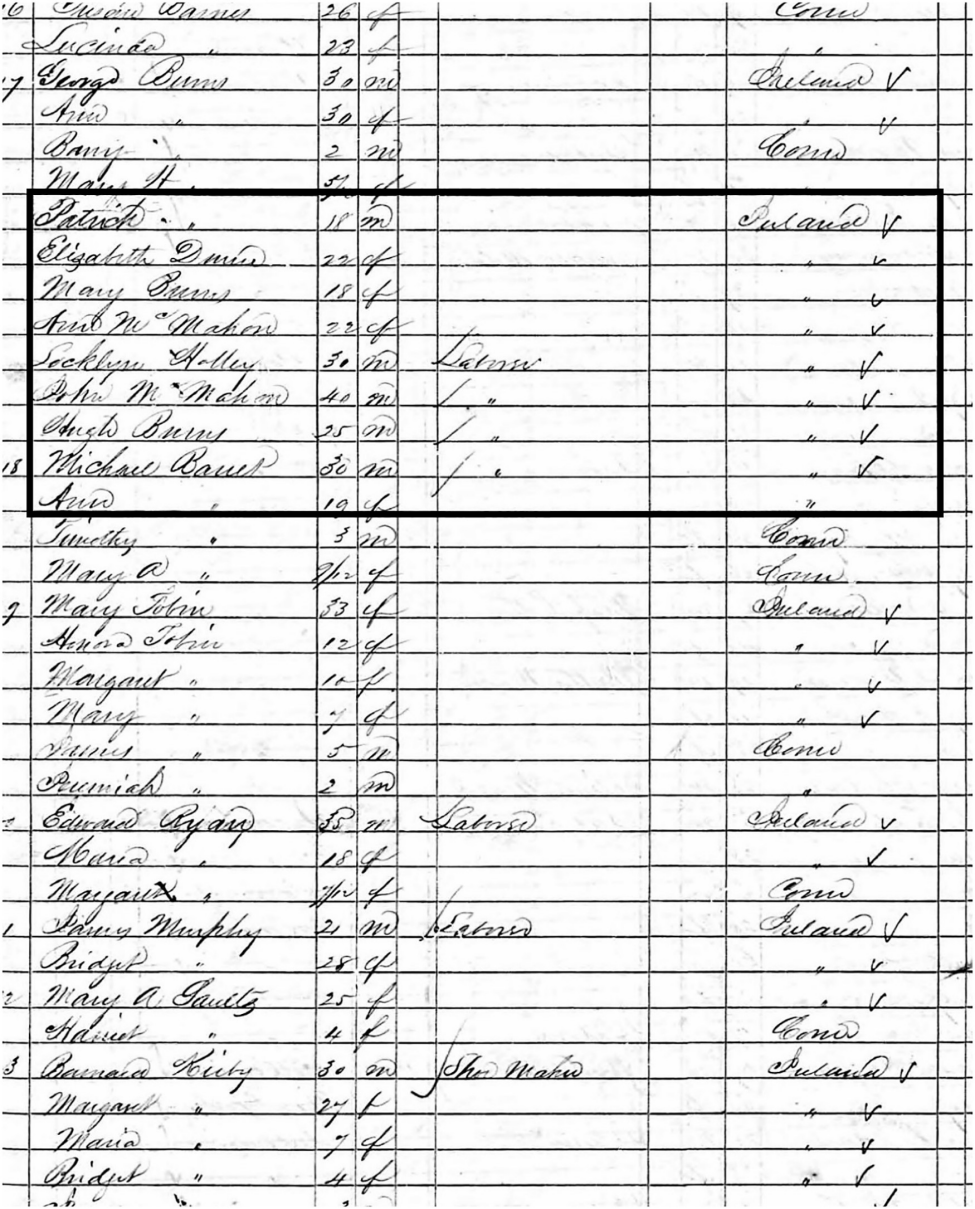
1850 New Haven Census and Country of Origin. Page indicating that a series of individuals are from “Poland”; surnames are consistent with Anglo-Saxon and/or Irish etymology. See text for details.

The Prussian and Austrian Empires encompassed German, Polish and southeastern European territories (including the Mediterranean coast), but the early to mid 19^th^ century was a period of marked unrest and sociopolitical upheaval [218–222]. Multiple reports of Polish/Slavic emigration across Europe and the Americas note the relatively unique cohesion of culture, language and community held by these emigrants [223–227]. Efforts to connect census data to the YNH4 skeletons were largely unsuccessful, as records for the city of New Haven provide limited data on ethnicity. Between 1830 and 1850, the number of Irish immigrants increased from less than a dozen to 3,533 families [228, 229]. At the same time, other ethnicities were present, but represented a small fraction of the population [230]. Available records of Prussian/Polish/German/Italian immigrants within New Haven indicate that most were skilled laborers or tradespeople [231–235]. Three households (Abraham Cohan, Henry Myers, and “Mr. Sobieski”) are recorded as being from Poland in the 1850 New Haven census, but their recorded ages (26, 26 and 30, respectively) are too young when compared to the YNH4 skeletal age estimates, and are excluded as potential matches. The 1850 Waterbury Census lists multiple individuals with anglicized surnames with “Poland” as the state of origin [228] but we cannot confirm accuracy versus census-taker recording/transcription errors (Fig 21). The Italian community was even smaller at this time—there is a single notation of a marriage between two Italians (“Geremia Gaudofo & Maria Gaheano”) on 12 April 1830 (at the first Baptist Church of New Haven) [236]. New Haven’s 1850 census data records four households with “Italy” as the country of origin—two have Anglicized surnames (Franklin & Roberts) and the other two are single men (Passani and Zephyr) [228]. Only 180 people are listed as Italian in the New Haven 1882 census [233]. There were no results for historic nations/principalities (i.e., Sardinia, Monaco, Lucca, etc.) or southeastern Europe states such as Romania or Bulgaria. While we cannot confidently associate the YNH4 with any specific ethnic group, our results are concordant with the historical record of non-Irish immigrants within New Haven at this relatively early period of American immigration.

As noted above, urban 19^th^ century immigrant life was difficult [177, 237] and Irish immigrants crowded into urban New Haven, taking advantage of economic opportunities [153] regardless of physical and emotional cost. At the same time, other non-Irish immigrants spread across New Haven County to find employment at the mills and factories populating the Naugatuck River. There, hydraulic power was used for metallurgy, fabric and rubber production [232, 238–242].

An important source for respite and solace was the Catholic Church [243–245]. American Roman Catholicism has a complex history [246–249] and immigrant churches struggled with limited community support and financial resources given their low socioeconomic status [103, 250, 251]. Procuring real estate and building materials was difficult, leading to very small plots of land and inexpensive buildings to accommodate rapidly growing congregations [243, 245, 246]–as evidenced by New Haven’s Christ Church [103]. Religion was an important component of 19^th^ century identity, but not for the Irish alone—more than 90% of the Polish national population was Catholic in the 1800s [252, 253] and Italy is historically a Catholic nation [254]. While social, cultural and linguistic factors distinguish the Irish/Prussian/Italian/Slavic immigrant experiences, shared Catholic faith and the few available churches led to religious identity superseding ethnicity [103, 251, 255–260]. Within New Haven, we suggest that individual/family socio-structural identity was similarly transformed—state- or ethnocentric identity was reduced in value/significance in favor of faith-based and communal Catholic identity [244, 245]. Such dueling (and associated) social identities have been described in other American populations [261–268]. As New Haven’s immigrant Catholic population grew, ethnicity resurfaced as a social identifier and solidified ethnolinguistic distinction between parishes (i.e., the “German” church of St. Boniface versus the “Italian” church of St. Michael) [103, 269].

While small Catholic churches spread across New Haven County in the mid 19^th^ century, all lacked an important component—a cemetery. From its consecration, New Haven’s Christ Church was the only cemetery for Catholics in the region, including outlying areas. Available burial records note interments of Derby, Waterbury, and Meridan residents (S1 Table). In 1854, a funeral procession moving down the Farmington Canal for interment in New Haven was described:

*“*[Catholic] *funerals* [in Waterbury] *were always largely attended*, *the entire Catholic community accompanying the remains to the cemetery at New Haven*. *The last funeral to go to New Haven was that of Captain Bannon…*”

(O’Donnell 1900: 383)

The Waterbury American of September 8, 1854, contained this editorial note:

*"An Irish funeral procession which passed our office on Saturday* [September 2] *was the largest we have ever seen in this city*. *It numbered twenty-four carriages and 304 persons on foot*, *128 of whom were females…"*

(Anderson et al. 1896: 732)

Within the New Haven Vital Statistics records, Catholic burials with Irish surnames are most common. However, these records do not account for all interments from outlying towns and villages, such as those along the Naugatuck River. We propose that YNH4 individuals A, B and B3 represent Catholic immigrants of southeastern/southern Europe origin, likely residing in an industrial town near the city of New Haven. Upon their deaths, they were brought to Christ Church for funerary rights and interment. Their burial along the fence line may represent geographic distinction within the Christ Church cemetery (i.e., Waterbury or Derby parishioners versus New Haven proper). Their non-Irish genetic identity, geochemical signatures, and musculoskeletal stress markers are consistent with this identification as adult, working class immigrants working in the industrialized New Haven region.

#### YNH4 Individual B2: Named individual identification?

Individual B2 is a middle-aged male, and while few genetic data were recovered, isotopic enamel biomarkers suggest a different origin and/or residence during second molar crown formation than the other YNH4 individuals. Postcranial elements exhibit manual labor indicators, but this individual is largely free of chronic illness biomarkers. However, perimortem trauma indicators are present for neck skeletal elements (Fig 16). The Christ Church burial records list 17 individuals where trauma is indicated as a cause of death; five are middle-aged/mature adult males (S1 Table). Specific details are not provided except for three cases: two with spinal fractures (J. Flood, aged 32, died 30 June 1842; J. Fagan, aged 36, died 07 June 1848) and one executed via judicial hanging (J. McCaffrey, aged 34, died 02 October 1850).

James McCaffrey was an Irish immigrant. Born in 1813 at Templeough, he left sometime around the age of seventeen for the United States. On arrival, he traveled as an itinerant laborer, visiting Quebec and working on steamers up and down the Mississippi to New Orleans and back. He spent time in upstate New York and Connecticut before coming to New Haven in 1847 [270]. While in New Haven in October 1849, he reportedly visited a couple (Ann and Charles Smith) who owned an inn and a bowling alley at the top of East Rock, a trap rock ridge overlooking the city. Shortly after his visit, the Smiths were found dead, shot and bludgeoned to death respectively. McCaffrey was named as the prime suspect based on material evidence (matching of a lead shot ball recovered from one murder victim to casting equipment that McCaffrey left behind) [271, 272]. McCaffrey had fled to Canada, but he was captured and tried for the murder of Ann Smith. The jury found him guilty, and he (and another convicted murderer) were executed behind the New Haven courthouse on October 2, 1850 [273]. Both were hung with knots set behind their respective left ears [272]. While there was a six-foot drop, both men remained alive, with McCaffrey’s heart beating until nine minutes after the drop. Post-execution, it was determined that McCaffrey suffered a broken neck. McCaffrey was placed into a "white wood [pine]" box, and taken to Christ Church and buried there that same day [272]. He was recorded as 37 years old at death.

We were unable to amplify nuclear DNA from Individual B2. Available genetic and craniometric data are consistent with European/Euro-American ancestry. The isotopic data indicate second molar crown development in a region consistent with USA and/or Northern Europe soils, based on the observed δ^18^O and Pb isotopes in the enamel (Table 4, Fig 20). The cervical injuries observed for YNH4 Individual B2 (Fig 16) are consistent with judicial hanging. While the classic “Hangman’s fracture” is not present, there is marked variation in the expression and frequencies of cervical fractures, even in explicitly judicial contexts [274–287]–factors such as knot placement, drop length and body mass play a role in vertebral damage presence and characteristics. As McCaffrey’s execution was performed under the standards and practices of the time [288, 289], we suggest that the observed damage to the hyoid and cervical vertebrae are consistent with [but not conclusive for] judicial hanging agency. Other Christ Church males have unspecified trauma as cause of death, making further elimination difficult. Given the available data, we provisionally identify Individual B2 as James McCaffrey of Templeough, Ireland.

## Conclusion

Evidence of structural violence, epidemiology, occupational stress, socioeconomic status and judicial action have been described for skeletal elements recovered from historic contexts [15, 17, 47, 204, 212, 213, 284, 290–292]. The YNH4 provides additional data for this record, using osteological, molecular, geochemical and archival variables to illuminate of immigrant origin, life history and social identity. Without such multidisciplinary methods, the YNH4 would likely have been identified as Irish immigrants in an early Catholic cemetery. In contrast, we report here on the remarkable diversity and complexity of the immigrant experience, describing biological markers of ancestry, indicators of mobility and stress, socioeconomic factors and the flexibility of ethnic and religious identities. Such examples of cultural embodiment over biological identity are critical for interpretation of historical, cultural and biological variables [1, 250, 293–300].

The title’s phrase “The Dead Shall Be Raised” (Corinthians 1: 15.52) is inscribed across the main gate of Grove Cemetery of New Haven CT, which was constructed in 1845. This passage connects an edifice built during the YNH4’s lives and deaths to our multidisciplinary efforts to illuminate their individual lives and see them as part of New Haven and New England’s immigrant history and identity. Our collaborative approach enhances the available narrative of Catholic immigrant history for this 19^th^ century industrial city and its neighbors. We have also provided a provisional identification of a known individual based on multiple lines of evidence. We strongly recommend this multidisciplinary approach for all cases where skeletal remains are recovered, given the complex interaction of human biology, social structure, and individual/communal identity.

## Supporting information

S1 Table Chronological data on burials (1834–1851) at Christ's Church & St. John's the Evangelist Churches of New Haven, Connecticut. (PDF)

S2 Table YNH4 osteometric data. (PDF)

S3 Table YNH4 nonmetric osteology data. (PDF)

S4 Table YNH4 FORDISC craniometric analyses results. (PDF)

S5 Table YNH4 postcranial entheses remodeling scores. (PDF)

S6 Table YNH4 dental calculus results. (PDF)
