# Supplementary material for: "The dead shall be raised": Multidisciplinary analysis of human skeletons reveals complexity in 19th century immigrant socioeconomic history and identity in New Haven, Connecticut
Source: PLoS One. 2019 Sep 9;14(9):e0219279. doi: 10.1371/journal.pone.0219279 (PMC6733446; doi:10.1371/journal.pone.0219279)
Supplement: S1 Table — (PDF) [file pone.0219279.s001.pdf]

Supplementary Table S1. Chronological data on burials (1834-1851) at Christ's Church St. John's the Evangelist Churches, Davenport Ave. & South St. New Haven, Connecticut. Individuals where trauma is recorded cause of death are in bold. Periods of infectious disease in bold box. See text for details.

Rec.	Month	Day	Year	Surname	Name of Person / Non-New Haven origin	Sex	Age (years)	Age Class	Recorded cause of death/Notes	Recoded Cause of Death
1	3	5	34	O'Connor	Mrs. John O'Connor (from Derby)	F	-	-		Not Recorded
2	3	5	34	McAuliff	James McAuliff	M	-	-		Not Recorded
3	5	5	34	Bohan	Daughter of Bartholmew Bohan	F	3	Preschool		Not Recorded
**4**	**5**	**5**	**34**	**Bryan**	**Abraham Lloyd Bryon (from Oxford)**	**M**	**14**	**Adolescent**	**Killed by falling Church Gallery (grandson)**	**Accident**
5	5	5	34	Healy	Ellen Healy	F	15	Adolescent		Not Recorded
**6**	**5**	**5**	**34**	**Harger**	**Abraham Hardyear [Harger] (from Derby)**	**M**	**-**	**-**	**Killed by falling Church Gallery (grandfather)**	**Accident**
7	5	5	34	Shea	Patrick Shea	M	-	-		Not Recorded
8	10	7	34	McGill	James McGill	M	8	Child		Not Recorded
9	10	7	34	Boden	Mary Boden alias Bohan	F	37	Middle Adult		Not Recorded
10	10	7	34	Hayes	Michael Hayes	M	37	Middle Adult		Not Recorded
11	10	7	34	Green	John Green	M	-	-		Not Recorded
12	10	7	34	Rice	John Rice an infant	M	-	Infant		Not Recorded
13	10	7	34	Rice	James Rice an infant	M	-	Infant		Not Recorded
14	7	20	35	Gregory	James Gregory	M	10	Child		Not Recorded
15	7	20	35	Whelan	Mrs. Whelan	F	-	-		Not Recorded
16	7	20	35	Driscoll	Cornelius Driscoll	M	-	-		Not Recorded
17	9	3	35	Franklin	James Franklin	M	1.17	Infant		Not Recorded
18	12	20	35	Franklin	Ann Franklin	F	3.5	Preschool		Not Recorded
19	12	20	35	Slevin	Philip Slevin	M	-	-		Not Recorded
20	1	9	36	Fitzpatrick	Fitzpatrick	U	3	Preschool		Not Recorded
21	1	9	36	McGirl	Anne McGirl	F	7	Child	Quinsey	Infectious
22	1	9	36	Unknown	A young woman	F	20	Young Adult		Not Recorded
23	1	9	36	Crowley	Crowley an infant	U	-	Infant		Not Recorded
24	1	9	36	Walsh	Walsh an infant	U	-	Infant		Not Recorded
25	4	6	36	Baron	Peter G. Baron	M	42	Middle Adult	Febris (alt. date: October 6, 1836)	Infectious
26	4	20	36	Larkin	Honora Larkin	F	32	Middle Adult	Pneumonitis (Alt date: Grave stone: Apr. 21, 1836)	Infectious
27	4	20	36	Fury	James Fury an infant	M	-	Infant	Febris	Infectious
28	4	25	36	Larkin	Catharine Larkin	F	2	Infant		Not Recorded
29	5	19	36	Meighan	James Meighan an infant	M	-	Infant		Not Recorded
30	6	4	36	McGinon	Michael McGinon	M	22	Young Adult	Pneumonitis	Infectious
31	6	6	36	Higgins	Edward Higgins	M	32	Middle Adult	Hydrops	Other
32	6	11	36	Murray	John Murray	M	2	Infant	Pneumonitis	Infectious
33	6	11	36	Unknown	a male infant	M	-	Infant		Not Recorded
34	6	19	36	Unknown	a male infant	M	-	Infant		Not Recorded
35	6	19	36	Unknown	a male infant	M	-	Infant		Not Recorded
36	7	23	36	McGuinness	Infant of Patrick McGuiness	U	-	Infant		Not Recorded
37	8	2	36	Clark	Patrick Clark	M	2	Infant	Pneumonitis	Infectious
38	8	7	36	Grier	John Grier	M	2	Infant	Worms	Infectious
39	8	15	36	Fitzpatrick	Bridget Fitzpatrick infant	F	-	Infant		Not Recorded
40	9	14	36	Dermody	James Dermody infant	M	-	Infant	Enteritis	Gastrointestinal
41	10	20	36	Curley	Thomas Curley	M	50	Mature Adult	Sudden, found dead	Other
42	11	9	36	Duffy	Duffy a male child	M	-	-		Not Recorded
43	11	24	36	Kelley	Thomas Kelley	M	38	Middle Adult	Bowel complaint	Gastrointestinal
44	12	25	36	Carroll	Philip Carroll	M	40	Middle Adult	Accident falling	Accident
45	1	15	37	Cody	Dennis Cody	M	2	Infant	Phthisis	Infectious
46	2	1	37	Cahill	Dennis Cahill	M	22	Young Adult		Not Recorded
47	2	6	37	Reynolds	Owen Reynolds	M	34	Middle Adult	Cold Catarrh	Infectious
48	2	13	37	Costillow	Infant of Costillow	U	-	Infant	unknown	Not Recorded
49	2	20	37	Cogan	Cogan	U	3	Preschool	Scarlatina	Infectious
50	2	26	37	McNulty	Ann McNulty	F	30	Young Adult	Diarrhoea	Gastrointestinal
**51**	**2**	**27**	**37**	**Flynn**	**Patrick Flynn**	**M**	**56**	**Mature Adult**	**Kick of horse**	**Accident**
52	4	18	37	Harnitte	James Harnitte	M	21	Young Adult		Not Recorded
53	6	19	37	Brady	James Brady [son of] Nicholas & Margaret	M	2	Infant	Church record (no city record) (see record 236)	Not Recorded
54	7	24	37	Downing	Gerald Downing (from Middletown)	M	-	-		Not Recorded
55	8	14	37	Shiel	Catherine Shiel	F	37	Middle Adult	Puerperal	Childbirth
56	9	24	37	Fare	William Fare or Hare (Henry Hare)	M	27	Young Adult	Phthisis [Tuberculosis] (stone: Sept 22, 1837)	Infectious
57	10	8	37	Carmedy	Patrick Carmedy	M	37	Middle Adult	Cold Catarrh	Infectious
58	11	15	37	Sullivan	Ann Sullivan	F	23	Young Adult	Phthisis [Tuberculosis]	Infectious
59	11	30	37	Landragan	Charles Landragan	M	2	Infant	Neckrosis	Other
60	12	27	37	Cook	Thomas Cook	M	30	Young Adult	Pneumonitis	Infectious
61	12	27	37	Mooney	Thomas Mooney	M	34	Middle Adult	Alt. data: stone; Dec 26, 1837 age 30	Not Recorded
**62**	**12**	**29**	**37**	**Healey**	**Timothy Healey**	**M**	**32**	**Middle Adult**	**Accident (by Waggon) [Wagon]**	**Accident**
63	2	2	38	Cook	Thomas Cook	M	24	Young Adult	Church record (no city record)	Not Recorded
64	2	4	38	Ledwidge	James Ledwidge	M	35	Middle Adult	Hemorrhage	Bleeding
65	3	7	38	Egan	Ann Egan	F	28	Young Adult	Phthisis [Tuberculosis]	Infectious
**66**	**5**	**14**	**38**	**Farigan**	**Unknown**	**M**	**22**	**Young Adult**	**Accident**	**Accident**
67	7	2	38	[Hannon] Fannon	Mary Fannon (Hannon)	F	23	Young Adult	Phthisis [Tuberculosis] (see record 97) (twin) (alt record: Jul. 10, 1838)	Infectious
68	7	2	38	Rigan	An infant of John Rigan	U	-	Infant		Not Recorded
69	7	6	38	Joice	Bridget wife of Patrick Joice	F	20	Young Adult		Not Recorded
70	8	11	38	Marlin	Child of Laurence Marlin	U	3	Preschool	Drowned	Accident
71	8	16	38	Hayden	Patrick Hayden	M	25	Young Adult		Not Recorded
72	10	4	38	Costello	an infant of James Costello	U	-	Infant		Not Recorded
73	10	5	38	Furley	an infant of John Furley	U	-	Infant		Not Recorded
74	10	6	38	Joice	an infant of Patrick Joice	U	-	Infant		Not Recorded
75	10	7	38	Reynolds	an infant of Thomas Reynolds	U	1	Infant		Not Recorded
76	10	7	38	Lahy	an infant of John Lahy	U	-	Infant		Not Recorded
77	10	7	38	Unknown	an infant	U	-	Infant		Not Recorded
78	10	7	38	Unknown	an infant	U	-	Infant		Not Recorded
79	10	27	38	Bannon	Thomas Bannon	M	35	Middle Adult	a cold	Infectious
80	11	2	38	Toner	Ann Tonor (Toner)	F	15	Adolescent	a cold	Infectious
81	11	10	38	Ready	Patrick Ready	M	28	Young Adult	Dropsy	Other
82	11	10	38	Keney	Patrick Keney	M	30	Young Adult	Drowned	Accident
83	11	10	38	White	Richard White	M	38	Middle Adult	Phthisis [Tuberculosis]	Infectious
84	11	10	38	Healy	Margaret Healy	F	-	-		Not Recorded
85	1	13	39	Crawley	Child of William Crawley	U	3m	Infant		Not Recorded
86	1	28	39	Nevil	John Nevil	M	24	Young Adult	Scalded	Accident
87	2	8	39	Crawley	Dennis Crawley (Crowley)	M	38	Middle Adult	Phthisis [Tuberculosis]	Infectious
88	6	10	39	Conlin	James Conlin (Conlan)	M	22	Young Adult	Drowned	Accident
89	7	1	39	New	John New (buried at Christ's Church)	M	27	Young Adult	Drowned (added to McNally at St. Bernard's)	Accident
90	7	1	39	Splain	John Splain	M	28	Young Adult	Bowel Complaint	Gastrointestinal
91	7	1	39	Galegan	Michael Galegan	M	30	Young Adult	Phthisis [Tuberculosis]	Infectious
92	7	1	39	Whelan	John Whelan	M	-	-	Phthisis	Infectious
93	9	16	39	Casey	Mary Casey	F	21	Young Adult		Not Recorded
94	10	15	39	Quinn	Owen Quinn	M	30	Young Adult	Phthisis [Tuberculosis]	Infectious
95	10	15	39	Hayes	James Hayes	M	40	Middle Adult	a cold	Infectious
96	11	8	39	Riley	James Riley (* Hale Record: Reilly)	M	24	Young Adult	Phthisis [Tuberculosis] (Alt record: 5/13/1839)	Infectious
97	12	*	39	Hannon	John Hannon (twin)	M	27	Young Adult	Phthisis [Tuberculosis] (see record 67)	Infectious
98	1	27	40	Lynch	John Lynch	M	3	Preschool	Feb Catarrhal	Infectious
99	1	27	40	Carlin	Patric Carlin	M	44	Middle Adult	Phthisis [Tuberculosis] (Consupmtion)	Infectious
100	1	27	40	Fury	Patrick Fury (Furey)	M	44	Middle Adult	Hydrops (added to wife's stone St. Bernard's)	Other
101	5	7	40	Towel	Michael Towel	M	3	Preschool	Pneumonitis	Infectious
102	5	14	40	Cannon	James Cannon	M	40	Middle Adult	Phhisis (sic)	Infectious
103	5	15	40	Coldwell	Mary Coldwell	F	27	Young Adult	Apoplexia	Bleeding
104	5	21	40	Monahan	Bridget Monahan	F	30	Young Adult	Phthisis/Consumption [Tuberculosis]	Infectious
105	6	10	40	Healy	Infant of John Healy	U	1d	Infant	Infantile	Not Recorded
106	6	10	40	Healy	Infant of John Healy	U	4d	Infant	Infantile	Not Recorded
107	6	13	40	Benham	Mary Benham	F	40	Middle Adult	Phthisis/Consumption [Tuberculosis]	Infectious
108	7	27	40	Healy	Dennis Healy	M	40	Middle Adult	Church record (no city record)	Not Recorded
109	7	29	40	Macintire	John Macintire (from Bridgeport)	M	32	Middle Adult	Intemperance	Liver Disease/Alcoholism
110	7	29	40	Carl	Laurence Carl	M	40	Middle Adult	Intemperance	Liver Disease/Alcoholism
111	7	29	40	Donnally	Cornelius Donnally	M	48	Mature Adult	Phthisis/Consumption [Tuberculosis]	Infectious
112	1	4	41	Delaney	Susannah Delaney	F	1.3	Infant	Church record (no city record) (see record 182)	Not Recorded
113	7	12	41	Gilmour	Mary Gilmour (Gilmer)	F	28	Young Adult	Erysipelas	Infectious
114	9	29	41	Hurley	James Hurley	M	37	Middle Adult	Church record (no city record)	Not Recorded
115	1	27	42	Donnegan	Margaret Donnegan (Dunigan [sp.] wife of James)	F	36	Middle Adult	Febr. Puerper	Childbirth
116	2	23	42	Horley	William Horley	M	28	Young Adult		Not Recorded
117	2	23	42	Corbitt	William Corbitt	M	42	Middle Adult		Not Recorded
**118**	**6**	**30**	**42**	**Flood**	**John Flood**	**M**	**32**	**Middle Adult**	**Fractured spine from a fall**	**Accident**

Supplementary Table S1. Chronological data on burials (1834-1851) at Christ's Church St. John's the Evangelist Churches, Davenport Ave. & South St. New Haven, Connecticut. Individuals where trauma is recorded cause of death are in bold. Periods of infectious disease in bold box. See text for details.										
Rec.	Month	Day	Year	Surname	Name of Person / Non-New Haven origin	Sex	Age (years)	Age Class	Recorded cause of death/Notes	Recoded Cause of Death
119	7	30	42	Dorland	Ann Dorland	F	24	Young Adult		Not Recorded
120	8	16	42	Riley	Daniel Riley	M	24	Young Adult	Phthisis [Tuberculosis]	Infectious
121	8	28	42	Cornlin	John Cornlin (from Salem)	M	22	Young Adult	Drowned at Salem	Accident
122	8	28	42	Donnally	James Donnally	M	25	Young Adult	Church record (no city record)	Not Recorded
123	10	12	42	Duff	Margaret Duff ([wife of] Simon)	F	25	Young Adult	Church record (no city record)	Not Recorded
124	11	28	42	Orstrop	John Orstrop	M	37	Middle Adult	Febr. Typhus	Infectious
125	1	10	43	Fitzpatrick	Child of Thomas Fitzpatrick	U	8	Child	Scrophula	Infectious
126	1	24	43	Leahy	Thomas Leahy	M	68	Elderly	Phthisis [Tuberculosis]	Infectious
127	1	30	43	Conaughly	Child of Thomas Conaughly	U	1	Infant		Not Recorded
128	2	28	43	Mooney	Emma Mooney	F	19	Young Adult	Puerperal	Childbirth
129	3	5	43	Brady	Infant of Hugh Brady	U	6m	Infant	(see record 328)	Not Recorded
130	3	10	43	Allen	Infant of Ed Allen	U	1	Infant		Not Recorded
131	3	10	43	Unknown	a child	U	2	Infant		Not Recorded
132	3	19	43	Murphy	Infant of Edward Murphy	U	2m	Infant		Not Recorded
133	3	22	43	Eastrop	John Eastrop	M	4d	Infant		Not Recorded
134	5	16	43	Unknown	An infant aged under	U	1	Infant		Not Recorded
135	5	16	43	Unknown	An infant aged under	U	1	Infant		Not Recorded
136	5	16	43	Unknown	An infant aged under	U	1	Infant		Not Recorded
137	5	16	43	Magil	Edward Magil	M	3	Preschool	Scarlatina	Infectious
138	6	1	43	Delaney	Catherine Delaney	F	3.6	Preschool	Church record (no city record)	Not Recorded
139	6	7	43	Mullen	Wife of Michael Mullen (Ann)	F	20	Young Adult		Not Recorded
140	6	11	43	McGrath	Infant of Thomas McGrath	U	-	-		Not Recorded
141	6	18	43	Boudren	Michael Boudren	M	40	Middle Adult	Phthisis [Tuberculosis]	Infectious
142	6	18	43	Leary	Infant of Daniel Leary	U	11m	Infant		Not Recorded
143	6	20	43	Flood	Mary Ann Flood	F	6	Child	Scarlatina	Infectious
144	8	9	43	Keornan	John Keornan	M	4	Preschool		Not Recorded
145	9	12	43	Bleagkley	Robert Bleagkley [son of] James & Susan	M	7	Child	Church record (no city record)	Not Recorded
146	9	12	43	Samon	Mary, wife of John Samon	F	45	Middle Adult		Not Recorded
147	9	29	43	Mooney	Julia, wife of Lawrence Mooney	F	29	Young Adult	Hydrops (stone: Sept. 19, 1843)	Other
148	11	4	43	Mury	Mary, daughter of Pat Mury	F	1.2	Infant		Not Recorded
149	11	9	43	Obey	Infant of Henry Obey	U	-	Infant		Not Recorded
150	1	1	44	Short	Bernard Short	M	50	Mature Adult	Fever	Infectious
151	1	4	44	Hall	John Hall	M	4	Preschool	Brain fever	Infectious
152	1	9	44	Brazil	Margarette Brazil	F	67	Elderly	Pneumonitis	Infectious
153	1	13	44	Quenlan	John Quenlan	M	4m	Infant		Not Recorded
154	2	2	44	O'Neal	John O'Neal	M	2	Infant	Brain fever	Infectious
155	2	8	44	Driscol	Dennis Driscol	M	17	Adolescent	Fever	Infectious
156	2	15	44	Hurley	John Hurley	M	53	Mature Adult	Pneumonitis	Infectious
157	2	28	44	Cold	John Cold	M	40	Middle Adult		Not Recorded
158	3	30	44	Galegan	James Galegan	M	4.5	Preschool	Scarlatina	Infectious
159	4	8	44	Galigan	Catherine Galigan	F	3	Preschool	Scarlatina	Infectious
160	4	8	44	Craveley	Ellen Craveley	F	1d	Infant	Infantile	Not Recorded
161	4	8	44	Lary	Bridget Lary	F	8m	Infant		Not Recorded
162	5	7	44	Healy	Catherine Healy	F	6	Child	Scarlatina	Infectious
163	5	7	44	Murphy	Philip Murphy	M	20	Young Adult	Phthisis [Tuberculosis]	Infectious
164	6	8	44	Ellis	Bridget Ellis	F	38	Middle Adult	Phthisis [Tuberculosis]	Infectious
165	6	18	44	Costolo	Child of Peter Costolo	U	5	Preschool	Scarlatina	Infectious
166	6	18	44	Costolo	Child of Peter Costolo	U	7m	Infant	Scarlatina	Infectious
167	6	22	44	Costolo	Child of Peter Costolo	U	3	Preschool	Scarlatina	Infectious
168	7	*	44	[Cosgrove] Cosgrif	Mary Cosgrif (Cosgrove) (wife of Henry)	F	35	Middle Adult	feb. Puerperal (stone: July 6, 1844)	Childbirth
169	8	1	44	Dwyer	Son of Patrick Dwyer	M	9m	Infant		Not Recorded
170	8	23	44	[D'Arcy] Darey	Chistopher Darey (D'Arcey)	M	28	Young Adult	Phthisis [Tuberculosis] (alt record: Aug. 22, 1844)	Infectious
171	9	29	44	Murphy	John Murphy	M	2.75	Infant		Not Recorded
172	11	2	44	Brady	Henry Brady ([son of] Hugh & Catherine)	M	-	-	Church record (no city record) (see record 328)	Not Recorded
173	11	6	44	Sheridan	Michael Sheridan	M	78	Elderly		Not Recorded
174	11	10	44	Pruhil	John Pruhil	M	4	Preschool	Phthisis	Infectious
175	12	27	44	Costolo	Matthew Costolo	M	30	Young Adult	Apoplexia	Other
176	1	11	45	Smith	Peter Smith	M	1.4	Infant	Febr. Catarrhal	Infectious
177	1	11	45	McManus	William McManus	M	20	Young Adult	Phthisis [Tuberculosis]	Infectious
178	3	11	45	Divine	Child of Patrick Divine	U	6	Child		Not Recorded
179	4	2	45	Reehel	Child of Terrence Reehel	U	1.5	Infant	Cynanche Trachealis	Infectious
180	4	19	45	Turner	Patrick Turner	M	28	Young Adult	Pneumonitis	Infectious
181	4	22	45	O'Connor	Bernard O'Connor	M	3	Preschool		Not Recorded
182	5	11	45	Delaney	Child (Susannah Delaney) (from Waterbury)	U	9m	Infant	from out of town (see records 112)	Not Recorded
183	7	20	45	Reynolds	Wife of Francis Reynolds	F	30	Young Adult		Not Recorded
184	7	20	45	Lee	John Lee	M	1d	Infant		Not Recorded
185	7	20	45	Farrell	Child of Thomas Farrell	U	3m	Infant		Not Recorded
186	7	20	45	Harrigan	Child of John Harrigan	U	5m	Infant		Not Recorded
187	8	8	45	Reynolds	Thomas Reynolds	M	35	Middle Adult	Phthisis [Tuberculosis] (alt record: August 7, 1845)	Infectious
188	8	8	45	Cawton Irish	Michael Cawton Irish	M	60	Mature Adult	Phthisis [Tuberculosis] at Almshouse	Infectious
189	8	11	45	Reynolds	Catherine Reynolds	F	9m	Infant		Not Recorded
190	8	24	45	Reynolds	Bridget Reynolds	F	2.5	Infant	Variolus	Infectious
191	8	27	45	Riley	Child of Patrick Riley (from Waterbury)	U	1m	Infant		Not Recorded
192	8	27	45	Costello	Child of Matthew Costello	U	6m	Infant		Not Recorded
193	9	16	45	Flood	Child of Richard Flood	U	2	Infant	Pertussis	Infectious
194	9	16	45	Downes	Michael Downes	M	52	Mature Adult	Apoplexia (see record 580)	Other
195	9	19	45	Flood	Bridget Flood (Mary [daughter of] Matthew & Bridget)	F	2	Infant	Pertussis (Vit. Stat. Bridget / stone: Mary)	Infectious
196	9	19	45	Knowland	Child of Daniel Knowland or Ouland	U	3m	Infant	Phthisis	Infectious
197	9	19	45	Millegan	Child of Michael Millegan	U	4m	Infant		Not Recorded
198	9	19	45	Caffrey	Child of Catherine Caffrey	U	6m	Infant		Not Recorded
199	10	22	45	Hurley	John Hurley	M	23	Young Adult	Phthisis [Tuberculosis]	Infectious
200	10	24	45	Gallager	Patrick Gallager (Gallagher)	M	30	Young Adult	Hydrops	Other
201	11	3	45	Finnegan	James Finnegan	M	25	Young Adult	Jaundice	Liver Disease/Alcoholism
202	11	3	45	Ryan	Child of Charles Ryan	U	6m	Infant	Pertussis	Infectious
203	12	7	45	Touhey	Ann Touhey [wife of] Patrick Touhey	F	53	Mature Adult	Church record (no city record)	Not Recorded
204	1	18	46	Meloy	Ellen Meloy	F	22	Young Adult	Insanity	Other
205	1	20	46	Nowland	Child of William Nowland	U	6m	Infant	Croup	Infectious
206	1	20	46	Wallace	Child of Thomas Wallace	U	7m	Infant	Hydrocephalus	Other
207	1	27	46	Gululy	Mary Gululy (Gilhuly)	F	21	Young Adult	Fever (alt record: Jan. 26, 1846)	Infectious
208	2	8	46	Reilly	Child of John Reilly	U	1m	Infant		Not Recorded
209	2	10	46	Sheridan	Mary Sheridan	F	26	Young Adult	Phthisis [Tuberculosis]	Infectious
210	3	7	46	Kane	Patrick Kane	M	48	Mature Adult	Pneumonitis	Infectious
211	3	7	46	Driscol	Child of Dennis O. Driscol	U	1m	Infant		Not Recorded
212	3	15	46	Kearns	John, son of Peter Kearns	M	2	Infant		Not Recorded
213	5	2	46	Kenarney	Martin Kenarney	M	40	Middle Adult	Church record (no city record)	Not Recorded
214	5	4	46	Foley	William Foley ([son of] Willam & Francis) (From Derby)	M	8	Child	(alt record: May 3 1846)	Not Recorded
215	5	5	46	McEnemy	Marie McEnemy	F	35	Middle Adult	Phthisis [Tuberculosis]	Infectious
216	6	27	46	Healy	Dennis Healy	M	40	Middle Adult	Delirium Tremens	Liver Disease/Alcoholism
217	7	12	46	Crawley	Catherine Crawley	F	9m	Infant	Pneumonitis	Infectious
218	7	14	46	McKay	Child of William McKay	U	6m	Infant		Not Recorded
219	7	21	46	Unknown	An infant	U	12d	Infant	Infantile	Not Recorded
220	8	15	46	McEnally	Child of Thomas McEnally	U	6m	Infant		Not Recorded
221	8	23	46	Gray	Bernard Gray	M	33	Middle Adult	Icterus (Grave stone: Aug. 28, 1846 age 36)	Liver Disease/Alcoholism
222	9	7	46	Costello	Child of Daniel Costello	U	1	Infant	Pneumonitis	Infectious
223	9	10	46	Gululy	James Gululy (Gilhuly)	M	25	Young Adult	Fever (Grave stone: Sept. 9, 1846)	Infectious
224	10	2	46	Barry	Widow Barry	F	34	Middle Adult	Phthisis [Tuberculosis]	Infectious
225	10	6	46	Malone	Mary Malone	F	30	Young Adult	Pneumonitis	Infectious
226	10	6	46	Bannon	Ann Bannon wife of Patrick	F	42	Middle Adult	Church record (no city record)	Not Recorded
227	10	8	46	O'Neal	Alice O'Neal	F	63	Mature Adult	Jaundice	Liver Disease/Alcoholism
228	10	10	46	Read	Mary Read (from Waterbury)	F	40	Middle Adult		Not Recorded
229	10	16	46	Costello	Mary Costello	F	3	Preschool	Dropsey	Other
230	11	8	46	Mooney	Alice Mooney	F	63	Mature Adult	Church record (no city record)	Not Recorded
231	12	17	46	Hurley	Timothy Hurley	M	63	Mature Adult	Pneumonitis	Infectious
**232**	**12**	**18**	**46**	**Halem**	**Bridget Halern (Hollaren)**	**F**	**31**	**Middle Adult**	**Accident (runaway horse) (alt record: Dec. 15 1846)**	**Accident**
233	12	19	46	Reily	Cicily Reilly	F	30	Young Adult	Paralysis	Other
234	1	5	47	Fhorty	Son of Michael Fhorty	M	1.4	Infant		Not Recorded
235	1	5	47	Flood	Matthew Flood	M	2.25	Infant	Fever	Infectious
236	1	31	47	Brady	Wife of Nicholas Brady (Margaret) (see 585)	F	37	Middle Adult	Delirium Tremens	Liver Disease/Alcoholism

Supplementary Table S1. Chronological data on burials (1834-1851) at Christ's Church St. John's the Evangelist Churches, Davenport Ave. & South St. New Haven, Connecticut. Individuals where trauma is recorded cause of death are in bold. Periods of infectious disease in bold box. See text for details.

Rec.	Month	Day	Year	Surname	Name of Person / Non-New Haven origin	Sex	Age (years)	Age Class	Recorded cause of death/Notes	Recoded Cause of Death
237	2	6	47	Carroll	Child of Patrick Carroll	U	3m	Infant	Small Pox	Infectious
238	2	17	47	Coogan	James Coogan	M	48	Mature Adult	Paralysis	Other
239	3	4	47	Dolosy	Ann Dolosy	F	35	Middle Adult	Phthisis [Tuberculosis]	Infectious
240	3	17	47	Keeshan	Wife of Dennis Keeshan	F	40	Middle Adult	Delirium Tremens	Liver Disease/Alcoholism
241	3	17	47	Dignon	Mary Dignon	F	3m	Infant	Convulsions	Other
242	3	18	47	Kain	Thomas Kain	M	60	Mature Adult	Pneumonitis	Infectious
243	3	18	47	Burns	Child of Michael Burns	U	1d	Infant	Infantile	Not Recorded
244	4	3	47	Smyth	Julia Smyth	F	25	Young Adult	Church record (no city record)	Not Recorded
245	4	7	47	Hogan	Michael Hogan	M	45	Middle Adult	Phthisis [Tuberculosis]	Infectious
246	4	8	47	Reynolds	Philip Reynolds	M	29	Young Adult	Ship Fever [Typhus]	Infectious
247	4	15	47	Phelan	Michael Phelan	M	21	Young Adult	Ship Fever [Typhus]	Infectious
248	4	16	47	McElroney	Son of Thomas McElorney	M	4	Preschool	Burn	Accident
249	4	28	47	Tiernan	Patrick Tiernan	M	35	Middle Adult	Drowned	Accident
250	5	5	47	Kidney	William Kidney	M	44	Middle Adult	Hematemesis	Bleeding
251	5	5	47	Smith	Rose, wife of Michael Smith	F	-	-	Phthisis	Infectious
252	5	6	47	Sheridan	Julia Sheridan	F	26	Young Adult	Puerperal	Childbirth
253	5	6	47	Turbott	Peter Turbott	M	27	Young Adult	Phthisis [Tuberculosis]	Infectious
254	5	17	47	McDonald	Andrew McDonald	M	16	Adolescent	Ship Fever [Typhus]	Infectious
255	5	18	47	Ryan	Patrick Ryan	M	34	Middle Adult	Drowned	Accident
256	5	18	47	Naily	Thomas Nally	M	49	Mature Adult	Ship Fever [Typhus]	Infectious
257	5	20	47	McGennis	Thomas McGennis	M	49	Mature Adult	Ship Fever [Typhus]	Infectious
258	5	27	47	Collins	Child of James Collins (from Meriden)	U	7	Child	died in Meriden	Not Recorded
259	5	27	47	Larmens	William Larmens	M	20	Young Adult	Fever	Infectious
260	6	10	47	Thomas	John Thomas	M	17	Adolescent	Ship Fever [Typhus]	Infectious
261	6	15	47	Burns	Thomas Burns	M	30	Young Adult	Ship Fever [Typhus]	Infectious
262	6	24	47	Furber	Child of Peter Furber	U	11m	Infant		Not Recorded
263	6	24	47	Nally	Child of Thomas Nally	U	11m	Infant		Not Recorded
264	6	24	47	[Cavanaugh] Kavanagh	Child of Thomas Kavanagh (& Margaret)	U	1m	Infant	(July 15, 1847 age 6 mos.) (see record 367)	Not Recorded
265	7	12	47	Reynolds	Ann Reynolds	F	32	Middle Adult	Ship Fever [Typhus]	Infectious
266	7	16	47	Reynolds	Thomas Reynolds	M	36	Middle Adult	Ship Fever [Typhus]	Infectious
267	7	22	47	Morgan	Child of Patrick Morgan	U	2	Infant	Colic	Other
268	7	23	47	McCahil	Ellen McCahil	F	26	Young Adult	Ship Fever [Typhus]	Infectious
269	7	23	47	Costello	Bridget Costello, wife of James	F	28	Young Adult	Dropsey	Other
270	7	24	47	Keornan	Son of Dan Keornan	M	2	Infant	Cholera Infantum	Infectious
271	7	26	47	Morgan	Daughter of Patrick Morgan	F	9	Child		Not Recorded
272	8	1	47	Strang	Richard Strang ([son of] Martin & Ann)	M	6m	Infant	Church record (no city record)	Not Recorded
273	8	3	47	Farl	Child of Thomas Farl	U	1	Infant		Not Recorded
274	8	4	47	Strong	Child of Martin Strong	U	2	Infant	Dysentery	Infectious
275	8	4	47	Carter	James Carter	M	18	Adolescent	Ship Fever [Typhus]	Infectious
276	8	14	47	Reynolds	Timothy Reynolds	M	14	Adolescent	Ship Fever [Typhus]	Infectious
277	8	15	47	Reynolds	Ann Reynolds	F	52	Mature Adult	Ship Fever [Typhus]	Infectious
278	8	20	47	Honan	Child of Laurena Honan	U	1.25	Infant	Cholera Infantum	Infectious
279	8	23	47	Ryan	George Ryan	M	15m	Infant	Cholera Infantum	Infectious
280	8	25	47	Green	John Green (James [son of] Patrick & Harriet)	M	6m	Infant	Cholera Infantum (August 17, 1848)	Infectious
281	8	31	47	Reynolds	John Reynolds	M	26	Young Adult	Phthisis [Tuberculosis]	Infectious
282	8	31	47	McCan	Mary F. wife of Thomas McCan	F	42	Middle Adult	Phthisis [Tuberculosis]	Infectious
283	9	6	47	Bohan	Son of Patrick Bohan	M	8m	Infant		Not Recorded
284	10	5	47	Shanley	Child of Patrick Shanley	U	7m	Infant		Not Recorded
285	10	7	47	Reynolds	Catherine Reynolds	F	46	Mature Adult	Ship Fever [Typhus]	Infectious
286	10	9	47	Flood	Child of Fel. Flood	U	4	Preschool	Hydrocephalus	Other
287	10	9	47	Unknown	A child	U	3m	Infant	Colic	Other
288	10	17	47	[Fogarty] Facarty	Widow Mary Facarty (Fogarty)	F	60	Mature Adult	Ship Fever [Typhus]	Infectious
289	10	*	47	Unknown	A child	U	4m	Infant	Cholera Infantum	Infectious
290	11	8	47	Early	Catherine Early	F	3	Preschool	Ship Fever [Typhus]	Infectious
291	11	30	47	Doyle	John Doyle	M	56	Mature Adult	Ship Fever [Typhus]	Infectious
292	12	8	47	Doyle	Mary Doyle	F	54	Mature Adult	Ship Fever [Typhus]	Infectious
293	12	11	47	Doyle	Ellen Doyle	F	14	Adolescent	Ship Fever [Typhus]	Infectious
294	12	11	47	Sweetman	Mary Sweetman	F	49	Mature Adult	Paralysis	Other
295	12	13	47	Sheridan	Son of Patrick Sheridan	M	-	-	Volvulus	Gastrointestinal
296	12	15	47	Brenan	Laurence Brenan	M	55	Mature Adult	Otitis	Other
297	12	16	47	Lynch	Son of Patrick Lynch	M	3	Preschool	Burn	Accident
298	12	16	47	Doyle	James Doyle	M	16	Adolescent	Ship Fever [Typhus]	Infectious
299	12	16	47	Laden	James Laden	M	52	Mature Adult	Pneumonitis	Infectious
300	1	8	48	Hanley Kerigan	Ann Hanley alias Kerigan	F	44	Middle Adult	Phthisis [Tuberculosis]	Infectious
301	1	12	48	Kehoe	Thomas Kehoe	M	43	Middle Adult	Fever (Grave stone: Jan 11, 1848)	Infectious
302	1	16	48	Boyle	Charles Boyle	M	22	Young Adult	Dysentery	Infectious
303	1	24	48	Kennedy	Thomas Kennedy (Kenedy)	M	18	Adolescent	Fever Typhus	Infectious
304	2	2	48	Costelo	Mark Costelo	M	62	Mature Adult	Colic	Other
305	2	8	48	Reynolds	Wife of Michael Reynolds	F	35	Middle Adult	Diarehea	Gastrointestinal
306	2	14	48	Reynolds	Patrick Reynolds	M	35	Middle Adult	Ship Fever [Typhus]	Infectious
307	2	17	48	Seeley	Michael Seeley (Seerey)	M	67	Elderly	Phthisis [Tuberculosis]	Infectious
308	3	2	48	Lavery	Peter Lavery	M	1	Infant		Not Recorded
309	3	2	48	Cannon	Patrick Cannon	M	28	Young Adult	Ship Fever [Typhus]	Infectious
310	3	6	48	Molahar	Peter Mohalar [son of] Peter & Mary	M	1.16	Infant	Church record (no city record)	Not Recorded
311	3	22	48	Rheel	Jeremey Rheel	M	36	Middle Adult	Phthisis [Tuberculosis]	Infectious
312	3	31	48	McMahon	Child of John McMahon	U	1	Infant		Not Recorded
313	4	1	48	Caffrey	Jane Caffrey	F	68	Elderly	Heart Disease of	Other
314	4	2	48	Coil	Francis Coil	F	32	Middle Adult	Brain Fever	Infectious
315	4	15	48	McLughlin	James McLughlin	M	28	Young Adult	Ship Fever [Typhus]	Infectious
316	4	15	48	Teehil	Child of Terence Teehil	U	11m	Infant		Not Recorded
317	4	15	48	Mulligan	Child of John Mulligan	U	5m	Infant		Not Recorded
318	5	1	48	Unknown	Child	U	6	Child		Not Recorded
319	5	1	48	Daily	Margaret Daily (Daley [wife of] John)	F	32	Middle Adult	Puerperal (alt record: May 8, 1848)	Childbirth
320	5	1	48	Gihuly	Michael Gilhuly	M	62	Mature Adult	Fever	Infectious
321	5	1	48	Reynolds	Child of John Reynolds	U	4m	Infant		Not Recorded
322	5	10	48	Nephain	Michael Nephain	M	40	Middle Adult	Porphyra Hemorrhagia	Malnutrition
323	5	14	48	Braidy	Child of Hugh Braidy (Mary A.)	U	4m	Infant	(Alt record: headstone May 10, 1848 - Brady) (see record 328)	Not Recorded
324	5	14	48	Maglins	Child of William Maglins	U	6m	Infant		Not Recorded
325	5	20	48	Lyons	Alexander Lyons	M	47	Mature Adult	Icterus	Liver Disease/Alcoholism
326	6	7	48	Hector	Wife of John Hector	F	22	Young Adult	Phthisis [Tuberculosis]	Infectious
**327**	**6**	**7**	**48**	**Fagan**	**Joseph Fagan**	**M**	**36**	**Middle Adult**	**Accident (broken back from a fall)**	**Accident**
328	6	22	48	Brady	Hugh Brady	M	38	Middle Adult	Phthisis [Tuberculosis] (see record 172 & 323) (Alt record: headstone June 30, 1848)	Infectious
329	6	22	48	Costelo	Widow Mary Costelo	F	56	Mature Adult	Accident (a fall)	Accident
330	6	25	48	Ryan	Child of William Ryan	U	1.3	Infant		Not Recorded
331	7	6	48	Fanigan	Rose Fanigan	F	68	Elderly	Heart Disease of	Other
332	8	4	48	Ryan	Catharine Ryan	F	36	Middle Adult	Phthisis [Tuberculosis]	Infectious
333	8	10	48	Colwell	Rose Colwell	F	32	Middle Adult	Fever	Infectious
334	8	13	48	Reynolds	Peter Reynolds	M	36	Middle Adult	Colic	Other
335	8	17	48	Sweeny	William Sweeny	M	26	Young Adult	Dysentery	Infectious
336	8	17	48	Kinsella	Mary Kinsella	F	37	Middle Adult	Febr. Puerperar	Childbirth
337	8	18	48	Harris	Henry Harris	M	40	Middle Adult	Drowned	Accident
338	8	21	48	Hughes	Bridget Hughes (Hale Record - Huges)	F	41	Middle Adult	Dysentery	Infectious
339	8	26	48	Sullivan	Mary Sullivan	F	5	Preschool		Not Recorded
340	8	26	48	Keep	John Keep	M	29	Young Adult	Dysentery	Infectious
341	8	26	48	McDermott	Bernard McDermott [son of] Michael & Catherine)	M	10m	Infant	Church record (no city record)	Not Recorded
342	8	27	48	McDermott	Ann McDermott	F	5m	Infant	Hydrocephalus	Other
343	8	28	48	Spang	Nicholas W. Spang	M	1	Infant		Not Recorded
344	9	2	48	McEveny	Thomas McEveny	M	36	Middle Adult		Not Recorded
345	9	3	48	Dignon	Thomas Dignon (stone: possibly Dickson)	M	30	Young Adult	Dysentery	Infectious
346	9	4	48	Daley	Patrick Daley	M	38	Middle Adult	Dysentery	Infectious
347	9	9	48	Clines	Michael Clines	M	23	Young Adult	Dysentery	Infectious
348	9	19	48	Hauser	Frances Hauser	F	26	Young Adult	Fever	Infectious
349	9	27	48	Gordon	James Gordon	M	1	Infant	Dysentery	Infectious
350	10	3	48	Reily	Cornelius Reilly	M	20	Young Adult	Ship Fever [Typhus]	Infectious
351	10	11	48	Ryan	Jane Ryan	F	24	Young Adult	Phthisis [Tuberculosis]	Infectious
**352**	**10**	**12**	**48**	**McCormick**	**Mary McCormick**	**F**	**21**	**Young Adult**	**Accident (a fall)**	**Accident**
**353**	**10**	**23**	**48**	**Foley**	**John Foley**	**M**	**27**	**Young Adult**	**Accident on Railroad**	**Accident**

Supplementary Table S1. Chronological data on burials (1834-1851) at Christ's Church St. John's the Evangelist Churches, Davenport Ave. & South St. New Haven, Connecticut. Individuals where trauma is recorded cause of death are in bold. Periods of infectious disease in bold box. See text for details.

Rec.	Month	Day	Year	Surname	Name of Person / Non-New Haven origin	Sex	Age (years)	Age Class	Recorded cause of death/Notes	Recoded Cause of Death
354	10	28	48	Sheridan	Owen Sheridan	M	5	Preschool	Dysentery	Infectious
355	11	4	48	Corcoran	William Corcoran	M	1	Infant		Not Recorded
356	11	5	48	Creed	Dorothy Creed	F	25	Young Adult	Fever Typhus (see record 357)	Infectious
357	11	5	48	Creed	Dorothy Creed [with] mother Dorothy (headstone)	F	-	-	(see record 356)	Not Recorded
358	11	12	48	Galagan	James Galagan	M	40	Middle Adult	Delirium Tremens	Liver Disease/Alcoholism
359	11	12	48	Lynn	Michael Lynne (Lynn)	M	40	Middle Adult	Pleuritis (alt record: Grave stone: Nov. 10, 1848)	Infectious
360	11	24	48	Ellis	Ellen Ellis	F	35	Middle Adult	Phthisis [Tuberculosis]	Infectious
361	11	27	48	Costelo	William Costelo	M	45	Middle Adult	Fever	Infectious
362	12	10	48	Harvey	Catharine Harvey	F	25	Young Adult	Killed (Homicide)	Vioence/Trauma
363	12	10	48	Coyle	Bridget Smith Coyle	F	50	Mature Adult	Phthisis [Tuberculosis]	Infectious
**364**	**12**	**14**	**48**	**Kennah**	**Thomas Kennah**	**M**	**28**	**Young Adult**	**Accident (Railroad)**	**Accident**
365	1	12	49	Fagan	Bridget Fagan	F	19	Young Adult	Fever	Infectious
366	1	12	49	Fagan	Bridget Fagan	F	19	Young Adult	Fever	Infectious
367	2	2	49	Cavanaugh	Margaret Cavanaugh (Kavanaugh)	F	27	Young Adult	Scarlatina (alt record: 2/1/1849) (see record 264)	Infectious
368	2	11	49	Scully	John Scully	M	30	Young Adult	Fever	Infectious
369	2	11	49	Hayes	Child of John Hayes	U	-	-		Not Recorded
370	2	11	49	McMullen	Child of Patrick McMullen	U	9m	Infant	Dysentery	Infectious
**371**	**2**	**20**	**49**	**Moran**	**Thomas Moran**	**M**	**29**	**Young Adult**	**Accident - Fall from a tree**	**Accident**
372	2	23	49	Unknown	a Child	U	-	-	Hooping Cough	Infectious
373	3	4	49	Faughran	Edward Faughran	M	4.5	Preschool		Not Recorded
374	3	4	49	McNally	Bridget McNally	F	52	Mature Adult		Not Recorded
375	3	6	49	Mooney	Thomas H. Mooney ([son of] Morgan & Jane T.)	M	1.12	Infant	Church record (no city record)	Not Recorded
376	3	6	49	**Bohan**	Charles Bohan	M	30	Young Adult	Phthisis [Tuberculosis]	Infectious
377	3	9	49	Rooche	James Rooche	M	60	Mature Adult	Fever	Infectious
378	3	18	49	Coggins	Esther Coggins	F	27	Young Adult	Fever	Infectious
379	3	18	49	Weir	James Weir	M	28	Young Adult	Fever	Infectious
380	3	25	49	Callighan	Eliza Callighan	F	40	Middle Adult	Phthisis [Tuberculosis]	Infectious
381	3	29	49	Cox	Ann Cox	F	60	Mature Adult	Decline, Mararsmus?	Malnutrition
382	4	2	49	**Bohan**	Margaret Bohan	F	30	Young Adult	Childbed	Childbirth
**383**	**4**	**6**	**49**	**Bohan**	**Thomas Bohan**	**M**	**19**	**Young Adult**	**Accident - Fracture**	**Accident**
384	4	16	49	Kennedy	Michael Kennedy	M	26	Young Adult	Phthisis [Tuberculosis]	Infectious
385	4	16	49	Herrick	Elizabeth Herrick	F	65	Mature Adult	Fever	Infectious
386	4	21	49	Connor	Michael Connor	M	26	Young Adult	Church record (no city record)	Not Recorded
387	5	11	49	Murphy	Catharine Murphy	F	4m	Infant		Not Recorded
388	5	15	49	Carmason	Mary Carmason	F	23	Young Adult	Dropsey	Other
389	5	23	49	McGuire	James McGuire	M	1.5	Infant	Fever	Infectious
390	6	15	49	Cassidy	Eliza Cassidy	F	-	-	Phthisis	Infectious
391	6	22	49	Dogherty	Martin Dogherty (Dougherty)	M	34	Middle Adult	Phthisis [Tuberculosis]	Infectious
392	7	9	49	Walsh	Robert Walsh	M	7	Child	Dysentery	Infectious
393	7	10	49	Connor	Mary Connor (wife of Patrick)	F	38	Middle Adult	Church record (no city record) (see record 578)	Not Recorded
394	7	21	49	Connelly	Anne Connelly	F	33	Middle Adult	Phthisis [Tuberculosis]	Infectious
395	7	25	49	O'Bryan	James O'Bryan	M	5	Preschool	Dysentery	Infectious
396	8	6	49	Philbrick	Michael Philbrick	M	22	Young Adult	Drowned	Accident
397	8	8	49	Reilly	John Reilly	M	1m	Infant		Not Recorded
398	8	10	49	Magrath	Ellen Magrath	F	1	Infant	Dysentery	Infectious
399	8	12	49	Stiney	Richard Stiney	M	-	-	Hydrocephalus	Other
400	8	14	49	Hayes	William Hayes	M	1.1	Infant	Convulsions	Other
401	8	17	49	McDonough	Michael McDonough	M	-	-		Not Recorded
402	8	19	49	Reilly	John Reilly	M	1.5	Infant	Cholera Infantum	Infectious
403	8	22	49	Costello	Michael Costello	M	35	Middle Adult	Cholera	Infectious
404	8	24	49	Brady	Mrs. Brady	F	-	-	Delirium Tremens	Liver Disease/Alcoholism
405	8	27	49	Mason	John Reilly Mason	M	1	Infant	Convulsions	Other
406	8	27	49	Shanley	Bernard Shanley	M	22	Young Adult	Phthisis [Tuberculosis]	Infectious
407	8	27	49	Bon/Dunn	Mrs. Bon (or Dunn)	F	-	-	Cholera	Infectious
408	8	27	49	McCormick	Thomas McCormick	M	8m	Infant		Not Recorded
409	9	1	49	Carroll	Mary Carroll	F	2.5	Infant	Dysentery & Convulsions	Infectious
410	9	2	49	Dunne	Eliza Dunne	F	70	Elderly	Cholera	Infectious
411	9	3	49	Brougham	John Brougham	M	30	Young Adult	Dysentery	Infectious
412	9	7	49	Blessing	Peter Blessing	M	32	Middle Adult	Choloera	Infectious
413	9	10	49	Taylor	Catherine Taylor	F	26	Young Adult	Dysentery	Infectious
414	9	13	49	Collins	James Collins	M	1	Infant	Dysentery	Infectious
415	9	14	49	Gayner	Mary Gayner	F	1.5	Infant	Dysentery	Infectious
416	9	14	49	Holcomb	Mary Holcomb	F	32	Middle Adult	Dysentery	Infectious
417	9	19	49	Donnegan	Patrick Donnegan	M	28	Young Adult	Cancer	Other
418	9	30	49	Hurley	Margaret Hurley	F	30	Young Adult	Dysentery	Infectious
419	9	30	49	Flynn	Michael Flynn	M	35	Middle Adult	Dysentery	Infectious
420	10	4	49	Gaffney	Thomas Gaffney	M	7m	Infant	Dysentery	Infectious
421	10	5	49	Flynn	Michael Flynn	M	37	Middle Adult	Dysentery	Infectious
422	10	6	49	Turbutt	William Turbutt	M	40	Middle Adult	Dysentery	Infectious
423	10	10	49	Martin	James Martin	M	35	Middle Adult	Dysentery	Infectious
424	10	18	49	Reynolds	John Reynolds	M	20	Young Adult	Fever, Typhus (alt record: Oct. 17, 1849)	Infectious
425	10	19	49	Reilly	John Reilly	M	26	Young Adult	Dysentery	Infectious
426	10	26	49	Hayes	Peter Hayes	M	23	Young Adult	Dysentery	Infectious
427	10	29	49	McGreevy	Mary McGreevy	F	24	Young Adult	Puerperal Convulsions	Childbirth
428	11	1	49	Murray	Bridget Murray	F	58	Mature Adult	Phthisis [Tuberculosis]	Infectious
429	11	20	49	Reilly	Catherine Reilly	F	24	Young Adult	Fever	Infectious
430	11	28	49	Costello	William Costello	M	46	Mature Adult	Church record (no city record) (see record 439)	Not Recorded
431	12	5	49	Klem	Mary C. Klem ([wife of] George J.)	F	32	Middle Adult	Phthisis [Tuberculosis] (alt record: Dec. 4, 1849)	Infectious
432	12	12	49	Toole	Ellen Toole	F	26	Young Adult	Fever	Infectious
433	12	26	49	Kalahan	Catharine Kalahan	F	23	Young Adult	Dysentery	Infectious
434	12	26	49	Gaynor	Walter Gaynor	M	75	Elderly	Dysentery	Infectious
435	1	10	50	Cogan	Margaret Cogan	F	22	Young Adult	Puerperal Fever	Childbirth
436	1	30	50	McSweeney	William McSweeny	M	1	Infant	Dysentery	Infectious
437	2	21	50	Sweeny	Onora Sweeny	F	68	Elderly	Consumption [Tuberculosis]	Infectious
438	2	22	50	Leary	Daniel Leary	M	48	Mature Adult	Consumption [Tuberculosis]	Infectious
439	3	1	50	Costello	Ellen Costello (Elena [wife of] William)	F	52	Mature Adult	Phthisis [Tuberculosis] (March 10, 1850) (see record 430)	Infectious
440	3	7	50	Sillwood	John Sillwood	M	-	-	Asphyxia	Other
441	3	8	50	Feelan	John Feelan	M	12d	Infant		Not Recorded
442	3	12	50	Welch	Morrice Welch	M	27	Young Adult	Pneumonia	Infectious
443	3	14	50	Blake	Elizabeth Blake	F	27	Young Adult	Childbirth	Childbirth
444	3	23	50	Daily	John Daily	M	8m	Infant	unknown	Not Recorded
445	4	1	50	Duffee	Ann Duffee	F	9	Child	Dysentery	Infectious
446	4	11	50	Tayla	Michael Tayla	M	15d	Infant	Hydracephalus	Other
447	4	18	50	Logan	Susan Logan	F	42	Middle Adult	Volvulus [?]	Gastrointestinal
448	4	22	50	Shields	Randall Shields	M	8	Child	Hydracephalus	Other
449	4	26	50	Fitzpatrick	Birdget FitzPatrick	F	26	Young Adult	Consumption [Tuberculosis]	Infectious
450	4	27	50	Magoveran	B. Magoveran	U	26	Young Adult	Church record (no city record)	Not Recorded
451	5	2	50	Leonard	Child of James Leonard	U	0	Infant	Stillborn	Other
452	5	13	50	Marrygan	Ann Marrygan	F	5	Preschool	unknown perhaps Ships Fever	Not Recorded
453	5	21	50	McGrath	Mary McGrath	F	5	Preschool	Railroad accident	Accident
454	5	27	50	Stanford	Thomas Stanford	M	5	Preschool	Pneumonitis	Infectious
455	5	30	50	Johnson	James Johnson	M	1m	Infant	Manasinus	Malnutrition
456	5	31	50	Galigan	Catharine Galigan	F	22	Young Adult	Phthisis [Tuberculosis] (alt record: Galligan May 28 1850)	Infectious
457	6	3	50	Marrigen	Catherine Marrigen	F	1	Infant	Ship Fever [Typhus]	Infectious
458	6	20	50	Peterson	Wilbro Paul Peterson	M	3	Preschool	Manasinus	Malnutrition
459	6	27	50	Carroll	James son of Patrick Carroll	M	21d	Infant	Convulsions, Police Off. Child	Other
**460**	**7**	**4**	**50**	**Welch**	**Honora Welch**	**F**	**10**	**Child**	**Accident - Discharge of Cannon**	**Accident**
461	7	13	50	Clancey	Son of James Clancey	M	0	Infant	Stillborn	Other
462	7	16	50	O'Neil	Mary O'Neil	F	42	Middle Adult	Dropsy	Other
463	7	25	50	Larry	Bridget Larry	F	22	Young Adult	Typhoid Fever	Infectious
464	7	25	50	Arthur	James Arthur	M	24	Young Adult	Consumption [Tuberculosis]	Infectious
**465**	**7**	**29**	**50**	**Costello**	**Thomas Costello**	**M**	**19**	**Young Adult**	**Accident - fall from a scaffold**	**Accident**
466	8	1	50	Keep	Daniel Keep (from Northford)	M	5m	Infant	Catarrhal (fever & teething)	Infectious
467	8	2	50	Laehy	Ellen, Child of John Laehy	F	11m	Infant	Cholera Infantum	Infectious
468	8	4	50	Bruin	Michael Bruin	M	-	-	Phthisis	Infectious
469	8	4	50	Milan	Thomas Milan	M	-	-	Phthisis	Infectious
470	8	7	50	McLaughlin	James McLaughlin	M	2m	Infant	Cholera Infantum	Infectious

Supplementary Table S1. Chronological data on burials (1834-1851) at Christ's Church St. John's the Evangelist Churches, Davenport Ave. & South St. New Haven, Connecticut. Individuals where trauma is recorded cause of death are in bold. Periods of infectious disease in bold box. See text for details.

Rec.	Month	Day	Year	Surname	Name of Person / Non-New Haven origin	Sex	Age (years)	Age Class	Recorded cause of death/Notes	Recoded Cause of Death
471	8	11	50	Bohan	Thomas Bohan	M	5m	Infant	Cholera Infantum	Infectious
472	8	12	50	Dermody	Lenora Dermody	F	36	Middle Adult	Inflamation of the Bowels	Gastrointestinal
473	8	13	50	McLaughlin	Eliza J. McLaughlin ([daughter of] Michael & Rosanna)	F	8.25	Child	Hemorragia	Bleeding
474	8	14	50	Cunningham	Andrew Cunningham	M	3m	Infant	Manasinus & Diarrehea	Malnutrition
475	8	20	50	Malcoy	Child of Edmund Malcoy	U	0	Infant	Stillborn	Other
476	8	27	50	Mynehan	Mary Mynehan	F	2.17	Infant	Convulsions	Other
477	8	31	50	Stanford	Margaret Stanford	F	1.12	Infant	Cholera	Infectious
478	8	31	50	McDermott	Bridge McDermott	F	1.17	Infant	Cough or Teething	Infectious
479	9	3	50	Cassidy	Philip Cassidy	M	16	Adolescent	Liver Disease	Liver Disease/Alcoholism
480	9	5	50	Flaherty	Mary Flaherty [daughter of] Patrick & Bridget	F	17	Adolescent	Phthisis (alt record: 9/3/1850)	Infectious
481	9	13	50	Murtagh	Mary Jane Murtagh	F	10m	Infant	Cholera	Infectious
482	9	15	50	Comasky	Bernard Comasky	M	1	Infant	Hydrocephalus	Other
483	9	15	50	Reilly	John Reilly	M	1	Infant	Dysentery	Infectious
484	9	17	50	Egan	Catherine Egan	F	1	Infant	Hydrocephalus	Other
485	9	18	50	Cassada	Francis Henry Cassada	M	1	Infant	Peritonail Inflamation	Infectious
486	9	18	50	Reynolds	Mrs. Reynolds (wife of James)	F	35	Middle Adult	Chronic Debility	Other
487	9	18	50	Galagher	James Galagher	M	48	Mature Adult	Paralysis	Other
488	9	22	50	Gilen	Bridget Gillen ([wife of] James Gillin)	F	53	Mature Adult	Abscess Lumbar (alt. record: 9/14/1850)	Other
**489**	**10**	**2**	**50**	**McCaffray**	**James McCaffray**	**M**	**34**	**Middle Adult**	**Executed by Hanging, New Haven Jail**	**Execution**
490	10	6	50	Reynolds	Bridget Reynolds	F	83	Elderly	Old age	Other
491	10	16	50	Kivlahern	John Kivlahern	M	1	Infant	Hydrocephalus	Other
492	10	17	50	Reynolds	Son of Bernard Reynolds	M	1	Infant	Infantile	Not Recorded
493	10	21	50	Stang	Martin Stang Jr.	M	2	Infant	Diarrehea	gastrointestinal
494	11	6	50	Farrell	Patrick Farrell	M	30	Young Adult	Dropsy	Other
495	11	25	50	McDermott	Patrick McDermott	M	26	Young Adult	Consumption [Tuberculosis]	Infectious
496	12	5	50	Bowen	Charles Bowen	M	66	Elderly	Church record (no city record)	Not Recorded
497	12	11	50	Faughnan	Michael Faughnan	M	17	Adolescent	Phthisis	Infectious
498	12	17	50	Naughton	Mary Naughton	F	5	Preschool	Bronchitis	Infectious
499	12	17	50	Crowly	Timothy Crowly	M	17	Adolescent	Phthisis	Infectious
500	1	2	51	Kennedy	Homora Kennedy	F	35	Middle Adult	Liver Disease	Liver Disease/Alcoholism
501	1	5	51	McGovern	Mary McGovern	F	44	Middle Adult	Puerperal Fever	Childbirth
502	1	13	51	Shanley	Bridget Shanley	F	36	Middle Adult	Uterine Hemorage	Childbirth
503	1	14	51	Bowen	Margaet Bowen	F	28	Young Adult	Phthisis [Tuberculosis]	Infectious
504	1	14	51	McGuire	Barney McGuire	M	60	Mature Adult	Intemperance	Alcoholism
505	2	15	51	Bohen	Terese Bohen ([daughter of] Ternes & Ann)	F	1	Infant	Church record (no city record)	Not Recorded
506	2	21	51	Neil	Catherine Neil	F	23	Young Adult		Not Recorded
507	2	21	51	Roarke	P. Roarke	U	10m	Infant		Not Recorded
508	2	24	51	McGrath	M. McGrath		35	Middle Adult		Not Recorded
509	2	24	51	White	John R. White	M	5m	Infant		Not Recorded
510	2	27	51	Healy	Margaret Healy	F	10	Child		Not Recorded
511	2	27	51	Jollyan	Patrick Jollyan	M	23	Young Adult		Not Recorded
512	2	28	51	Gorman	James Gorman	M	7m	Infant		Not Recorded
513	3	2	51	Brady	Bernard Brady	M	50	Mature Adult		Not Recorded
514	3	5	51	Bryan	James Bryan	M	1	Infant		Not Recorded
515	3	5	51	Lynch	Michael Lynch	M	1	Infant		Not Recorded
516	3	5	51	Colman	John Colman	M	-	-		Not Recorded
517	3	7	51	Flaherty	Mary Ann Flaherty	F	8	Child		Not Recorded
518	3	7	51	Kennedy	John Kennedy	M	28	Young Adult	Fever	Infectious
519	3	7	51	Hogan	Michael Hogan	M	55	Mature Adult		Not Recorded
520	3	14	51	Peterson	Rosanna Peterson	F	1	Infant		Not Recorded
521	3	18	51	Whelen	Edward Whelen	M	1	Infant	Convulsions	Other
522	3	20	51	Sweeney	George Sweeney	M	2d	Infant		Not Recorded
523	3	27	51	Walsh	Mary Walsh	F	42	Middle Adult	Phthisis [Tuberculosis]	Infectious
524	3	28	51	Sullivan	Andrew Sullivan	M	40	Middle Adult		Not Recorded
525	3	31	51	Keefe	John Keefe	M	18	Adolescent	Ship Fever [Typhus]	Infectious
526	3	31	51	Nowland	Richard Nowland	M	1d	Infant		Not Recorded
527	4	1	51	Healy	Ann Healy	F	5	Preschool	Ship Fever [Typhus]	Infectious
528	4	6	51	Smith	John Smith	M	50	Mature Adult	Fever	Infectious
529	4	19	51	McDonough	Michael McDonough	M	1	Infant		Not Recorded
530	4	25	51	Grooms	Ellen Grooms	F	32	Middle Adult	Phthisis [Tuberculosis]	Infectious
531	5	1	51	Hackett	Sarah Hackett	F	17	Adolescent	Ship Fever [Typhus]	Infectious
532	5	11	51	Galligan	Andrew Galligan	M	45	Middle Adult	Typhoid Fever	Infectious
533	6	10	51	Riley	Mary Riley	F	67	Elderly	Disease of Liver	Liver Disease/Alcoholism
534	6	13	51	Judge	Mary Judge	F	1	Infant	Convulsions	Other
535	6	16	51	Fanny	Catherine Fanny	F	1	Infant	Bron. Measles	Infectious
536	6	18	51	Field	Child of John Field	U	14d	Infant	Convulsions	Other
537	6	20	51	Walsh	Child of Patrick Walsh	U	-	-	Croup	Infectious
538	6	21	51	Toole	Mary Toole	F	7m	Infant	Bron. Measles	Infectious
539	6	23	51	Conlan	Thomas Conlan	M	4m	Infant	Hydrocephalus	Other
540	6	24	51	Foley	Michael Foley	M	1m	Infant	Sudden Death	Other
541	6	26	51	Riordan	Margaret Riordan child of John Riordan	F	1	Infant	Marasmus	Malnutrition
542	6	29	51	Cummings	Alice Cummings	F	2	Infant	Pneumonia	Infectious
543	7	1	51	Lahey	John Lahey	M	50	Mature Adult	Pneumonia	Infectious
544	7	5	51	Holland	Richard Holland	M	5.5	Preschool	Convulsions	Other
545	7	6	51	Smith	Elizabeth Smith	F	43	Middle Adult	Phthisis [Tuberculosis]	Infectious
546	7	7	51	Kelly	Margaret Kelly	F	3	Preschool	Marasmus	Malnutrition
547	7	7	51	Creamer	Margaret Creamer	F	-	-	Chronic Bronchitus	Infectious
548	7	11	51	McGee	John McGee	M	34	Middle Adult	Bilious Fever	Infectious
549	7	12	51	Connelly	Catherine Connelly	F	33	Middle Adult	Dysentery	Infectious
550	7	22	51	O'Brien	Child of Patrick O'Brien	U	4	Preschool	Marasmus	Malnutrition
551	7	22	51	Morgan	Catherine Morgan	F	24	Young Adult	Miscarriage	Childbirth
552	7	23	51	Connelly	Elizabeth Connelly	F	2m	Infant	Cholera Infantum	Infectious
553	7	23	51	Loobey	Child of Andrew Loobey	U	2m	Infant	Cholera Infantum	Infectious
554	7	23	51	Kurnana	John Kurnana	M	7m	Infant	Convulsions	Other
555	7	23	51	Leahy	Child of John Leahy	U	9m	Infant	Catarrhal Fever	Infectious
556	7	27	51	Maxwell	Michael Maxwell	M	1.1	Infant	Cholera Infantum	Infectious
557	7	29	51	Clancey	Margaret Clancey	F	23d	Infant	Atrophy	Other
558	7	30	51	Clancey	Catherine Clancey	F	24d	Infant	Atrophy	Gastrointestinal
559	7	30	51	McGill	Ellen McGill	F	6m	Infant	Cholera Infantum	Infectious
560	8	2	51	Morgan	Ellen Morgan	F	40	Middle Adult	Dysentery	Infectious
561	8	4	51	Boyle	Child of Thomas Boyle	U	7m	Infant	Dysentery	Infectious
562	8	7	51	Powers	Mary Powers	F	14d	Infant	Convulsions	Other
563	8	9	51	Sherden	John Sherden	M	35	Middle Adult	Injury	Accident
564	8	10	51	Dooley	Sarah Jane Dooley	F	2.25	Infant	Cholera Infantum	Infectious
565	8	12	51	Burnz	Richard Burnz	M	42	Middle Adult	Consumption [Tuberculosis]	Infectious
566	8	13	51	McMullen	Henry McMullen	M	1.5	Infant	Cholera Infantum	Infectious
567	8	14	51	McGlinn	Ann McGlinn	F	1	Infant	Cholera Infantum	Infectious
**568**	**8**	**15**	**51**	**Lenner**	**Patrick Lenner**	**M**	**-**	**-**	**Injury**	**Accident**
569	8	19	51	McQueeny	Celia McQueeny	F	4.67	Preschool	Drowning	Accident
570	8	21	51	Sweeney	Infant of Michael Sweeney	U	10m	Infant	Hydrocephalus	Other
571	8	21	51	Miglinn	James Miglinn	M	6m	Infant	Congenital Phthisis	Infectious
572	8	22	51	Mahoney	John Mahoney	M	8m	Infant	Cholera Infantum	Infectious
573	8	25	51	Farall	Thomas Farall	M	6m	Infant	Cholera Infantum	Infectious
574	8	28	51	Keefe	Michael Keefe	M	7	Child	Bronchitis & Measels	Infectious
575	8	28	51	Ledwith	Elizabeth Ledwith	F	17	Adolescent	Encysted Ovarian Tumor	Other
576	9	1	51	Connolly	James Connolly	M	11m	Infant	Cholera Infantum	Infectious
577	9	7	51	Cullen	Rose Cullen	F	18	Adolescent		Not Recorded
578	9	18	51	Connor	Patrick Connor	M	40	Middle Adult	(see record 393)	Not Recorded
579	1	8	52	Conway	Alice Conway	F	32	Middle Adult	Uterine Hemorage	Bleeding
580	4	4	52	Downes	Bridget Downes	F	56	Mature Adult	(see record 194)	Not Recorded
581	9	9	52	Coleman	William Coleman	M	62	Mature Adult		Not Recorded
582	12	20	55	Mooney	Morgan Mooney	?	38	Middle Adult	Church record (no city record)	Not Recorded
583	2	28	57	McNulty	Willliam McNulty	M	30	Young Adult	Church record (no city record)	Not Recorded
584	1	*	41?	Costello	Child 1 of 7 (John Costello)	U	2.5	Infant	(headstone June 19, 1841)	Not Recorded
585	1	*	41?	Costello	Child 1 of 7 (Catherine Costello)	U	5.5	Preschool	(headstone June 16, 1841)	Not Recorded
586	1	*	41?	Costello	Child 1 of 7 (William Costello)	U	7m	Infant	(headstone June 16, 1841)	Not Recorded
587	8	7	11 (41?)	Flood	Jane Flood [daughter of] Matthew and Bridget (error ?)	F	1.3	Infant	Unknown - headstone at Christ's Church	Not Recorded
588	6	2	31(41?)	Shay	Patrick Shay (transcription error ?)	M	55	Mature Adult	Unknown - headstone at Christ's Church	Not Recorded

Supplementary Table S1. Chronological data on burials (1834-1851) at Christ's Church St. John's the Evangelist Churches, Davenport Ave. & South St. New Haven, Connecticut. Individuals where trauma is recorded cause of death are in bold. Periods of infectious disease in bold box. See text for details.

Rec.	Month	Day	Year	Surname	Name of Person / Non-New Haven origin	Sex	Age (years)	Age Class	Recorded cause of death/Notes	Recoded Cause of Death
589	*	*	62 (42?)	Mayer	Caroline Mayer	F	11m	Infant	Church record (no city record)	Not Recorded
590	9	11	94(54?)	Ryan	Catherine Ryan	F	29	Young Adult	Church record (no city record)	Not Recorded
591	9	6	84(?)	Ryan	Henry Ryan	M	36	Middle Adult	Church record (no city record)	Not Recorded
592	1	*	?	Unknown	Child 1 of 7	U	1	Infant		Not Recorded
593	1	*	?	Unknown	Child 1 of 7	U	1	Infant		Not Recorded
594	1	*	?	Unknown	Child 1 of 7	U	1	Infant		Not Recorded
595	1	*	?	Unknown	Child 1 of 7	U	1	Infant		Not Recorded
596	1	*	?	Boyd	Child (Nellie Boyd [daughter of] William & Mary)	U	3	Preschool		Not Recorded
597	7	*	?	Mackay	Son of Archy Mackay	M	5	Preschool		Not Recorded
598	7	*	?	Corcoran	Infant of John Corcoran	U	>1	Infant		Not Recorded
599	8	*	?	Unknown	An infant	U	-	Infant		Not Recorded
600	9	*	?	Logan	Child of John Logan	U	3m	Infant	Cholera Infantum	Infectious
601	12	*	?	Hart	Miss Hart	F	40	Middle Adult		Not Recorded
602	12	*	?	Caffrey	Child of Patrick Caffrey	U	1m	Infant		Not Recorded
603	*	*	?	Unknown	a boy	M	3	Preschool	Hydrocephalus	Other
604	*	*	?	Reed	Ann Reed	F	-	-	Unknown - headstone at Christ's Church	Not Recorded
605	*	*	?	Unknown	per Father Smyth's recollection 5 infants	F	-	Infant	Church record (no city record)	Not Recorded
