# Supplementary material for: "The dead shall be raised": Multidisciplinary analysis of human skeletons reveals complexity in 19th century immigrant socioeconomic history and identity in New Haven, Connecticut
Source: PLoS One. 2019 Sep 9;14(9):e0219279. doi: 10.1371/journal.pone.0219279 (PMC6733446; doi:10.1371/journal.pone.0219279)
Supplement: S2 Table — (PDF) [file pone.0219279.s002.pdf]

Supplementary Table S2. Osteometric data (mm). Mean and Standard Deviations (parentheses). Blank = element too damaged to measure or missing.

		A	B	B2	B3
Cranium	GOL (Maximum Cranial Length)	-	-	7Q1E3:F36E3	191.99 (0.52)
	XCB (Maximum Cranial Breadth)	-	-	139.3 (0.24)	140.3 (0.84)
	ZYB (Bizygomatic Breadth)	-	-	130.9 (0.25)	-
	BBH (Basion-Bregma Height)	-	-	127.7 (0.22)	129.1 (0.3)
	BNL (Cranial Base Length)	-	-	100.2 (0.08)	104.4 (0.24)
	BPL (Basion-Prosthion Length)	-	-	93.37 (0.13)	96.21 (0.43)
	MAB (Maxillo-Alveolar Breadth)	-	-	53.3 (0.82)	-
	MAL (maxilla-Alveolar Length)	-	-	-	56.5 (0.53)
	AUB (Biauricular Breadth)	-	-	122 (0.28)	119.6 (0.39)
	UFHT (Upper Facial Height)	-	-	70.8 (0.23)	69.4 (0.14)
	WFB (Minimum Frontal Breadth)	-	-	93.5 (0.62)	94.4 (0.11)
	UFBR (Upper Facial Breadth)	-	-	101.6 (0.02)	106.3 (0.09)
	NLH (Nasal Height)	-	-	51.1 (0.41)	50 (0.4)
	NLB (Nasal Breadth)	-	-	20.7 (0.21)	19.7 (0.13)
	OBB (Orbital Breadth)	-	-	37.7 (0.35)	43 (0.38)
	OBH (Orbital Height)	-	-	36.7 (0.22)	45.3 (0.57)
	EKB (Biorbital Breadth)	-	-	90.7 (0.59)	99.2 (0.34)
	DKB (Interorbital Breadth)	-	-	19.9 (0.15)	17 (0)
	FRC (Frontal Chord)	-	-	112.3 (0.17)	117.9 (0.32)
	PAC (Parietal Chord)	-	-	114 (0.64)	96.9 (0.31)
	OCC (Occipital Chord)	-	-	91.5 (0.48)	104.4 (0.71)
	FOL (Foramen Magnum Length)	-	-	40.3 (0.18)	38.2 (0.07)
	FOB (Foramen Magnum Breadth)	-	-	-	27.8 (0.11)
	MDH (Mastoid Height)	-	-	28 (1)	32.4 (0.93)
	ASB (Biasterionic Breadth)	-	-	112.1 (0.15)	110 (1.11)
	MOW (Mid-orbital Width)	-	-	37.2 (0.48)	-
	ZMB (Zygomaxillary Breadth)	-	-	86.1 (0.07)	91.2 (0.24)
Mandible	GNI (Chin Height)	-	-	30.1 (0.22)	31.1 (0.42)
	HMF (Mandibular Body Height at Mental Foramen)	-	-	15.8 (0)	-
	TMF (Mandibular Body Thickness at Mental Foramen)	-	-	13.2 (0.17)	-
	GOG (Bigonial Width)	-	-	93 (0.04)	102 (0.76)
	CDL (Bicondylar Breadth)	-	-	125.9 (0.13)	-
	WRB (Minimum Ramus Breadth)	-	-	32.9 (0.06)	-
	MLN (Mandibular Length)	-	-	-	67.3 (0.58)
	XRH (Maximum Ramus Height)	-	-	63.1 (0.43)	-
Clavicle	Max Length	129 (0.0)	-	-	158 (0)
	Circumference at Middle	3.5 (0.06)	-	-	32.3 (0.12)

		A	B	B2	B3
Humerus	Max Length	-	-	-	336 (0)
	Biomechanical Length	-	-	-	330 (0)
	Biepicondylar Width	-	-	-	39.4 (0.1)
	Max Midshaft Diameter	-	-	-	20.8 (0.12)
	Min Midshaft Diameter	-	-	-	16.6 (0.1)
Radius	AP Midshaft Diameter	-	-	-	11.9 (0)
	ML Midshaft Diameter	-	-	-	14.9 (0.06)
Ulna	Max Length	-	-	230.5 (0.32)	265.7 (0.58)
	Biomechanical Length	-	-	-	240.8 (0.31)
	Physiological Length	-	-	14.5 (0.1)	232.4 (0.06)
	Max AP Diameter	-	-	17.2 (0.12)	12.5 (0.11)
	Max ML Diameter	-	-	-	18 (0.06)
	Min Circumference	-	406 (0)	445 (0)	-
Pelvis	Os Coxae Height	-	402.7 (0.6)	441 (0)	-
	Iliac Length	-	86.7 (0.29)	46.5 (0.2)	-
	Pubic Length	-	-	88.5 (0.06)	-
	Acetabulosymphyseal Length	-	-	90.5 (0.5)	-
	Ischial Length	-	27.7 (0)	-	97.4 (0.32)
	Acetabular Height	-	26.9 (0.06)	-	54.9 (0.51)
	Acetabular Depth	-	333 (0)	29.1 (0.06)	-
	Obturator Foramen Length	-	-	27.9 (0.06)	55.2 (0.21)
	Obturator Foramen Breadth	-	-	369.7 (0.58)	32.5 (0.55)
Femur	Max Length	-	-	-	461.3 (0.58)
	Biomechanical Length	-	77.7 (0.58)	64.5 (0.1)	-
	Bicondylar Length	-	84.7 (0.58)	45.7 (0.21)	-
	Max Head Diameter	-	27.7 (0.1)	80.7 (0.06)	48.3 (0.15)
	Midshaft Circumference	-	21.1 (0.06)	99.4 (0.15)	87.7 (0.06)
	Midshaft Circumference	-	21.5 (0.12)	28.6 (0.12)	89.7 (0.29)
	Epicondylar Width	-	-	24.3 (0.1)	-
	Torsion	-	-	27.5 (0.06)	-
	AP Midshaft Diameter	-	-	354 (0)	30.3 (0.1)
	ML Midshaft Diameter	-	-	14.6 (0.1)	27.1 (0.06)
Tibia	Max Length	-	-	11.4 (0.12)	-
	Biomechanical Length- Medial	-	-	42.4 (0.99)	-
	Circumference at NF	-	-	-	91 (0.5)
	Cicumference at Nutrient Foramen	-	-	-	89.9 (0.2)
	ML Midshaft Diameter	-	-	-	25.3 (0.06)
	ML Shaft Diameter at NF	-	-	-	25.1 (0.1)
Fibula	Max Length	-	-	-	360 (0)
