# Supplementary material for: "The dead shall be raised": Multidisciplinary analysis of human skeletons reveals complexity in 19th century immigrant socioeconomic history and identity in New Haven, Connecticut
Source: PLoS One. 2019 Sep 9;14(9):e0219279. doi: 10.1371/journal.pone.0219279 (PMC6733446; doi:10.1371/journal.pone.0219279)
Supplement: S3 Table — (PDF) [file pone.0219279.s003.pdf]

**Supplementary Table S3. Nonmetric data scoring following Buikstra and Ubelaker [70] and Hauser and De Stefano [72]. L= left, R=right. X = feature too damaged to measure or missing.**

| | A | B | B2 | B3 |
|---|---|---|---|---|
| Anterior Nasal Spine | X | X | X | 3 |
| Inferior Nasal Aperture | 3 | X | 3 | 4 |
| Interorbital Breadth | 2 | 2 | 1 | 1 |
| Malar Tubercle | 1 | 0 | 1 | 0 |
| Nasal Aperture Shape | X | X | 1 | 1 |
| Nasal Aperture Width | X | X | 1 | 1 |
| Nasal Bone Contour | 1 | 4 | 3 | 4 |
| Nasal Bone Shape | 2 | 3 | 2 | 2 |
| Nasal Overgrowth | X | X | X | |
| Nasofrontal Suture | 1 | 1 | 2 | 2 |
| Orbital Shape | 2 | X | 2 | 1 |
| Post-Bregmatic Depression | 0 | X | 0 | 0 |
| Posterior Zygomatic Tubercle | 1 | 0 | 0 | 0 |
| Supranasal suture | 2 | 0 | 0 | 2 |
| Zygomatiocomaxillary Suture | 0 | X | 0 | X |
| Transverse Palatine Suture | X | 1 | X | X |

| | A | | B | | B2 | | B3 | |
|---|---|---|---|---|---|---|---|---|
| | L | R | L | R | L | R | L | R |
| Infraorbital suture | X | X | 0 | 0 | 2 | 0 | X | X |
| Infraorbital foramen | X | X | X | X | 3 | 3 | X | 0 |
| Zygomaticofacial foramen | X | 0 | X | X | 3 | 3 | 1 | 1 |
| Metopic Suture | 1 | | X | | 0 | | 1 | |
| Supraorbital notch | 1 | 1 | X | X | 1 | 0 | 1 | 1 |
| Supraorbital formaen | 0 | 0 | X | 0 | 1 | 0 | 0 | 1 |
| Supratrochlear notch | X | 0 | X | X | 0 | 0 | 0 | 1 |
| Coronal ossicle | 0 | 0 | X | X | 0 | 0 | 0 | 0 |

| | A | | B | | B2 | | B3 | |
|---|---|---|---|---|---|---|---|---|
| | L | R | L | R | L | R | L | R |
| Epipteric bone | 0 | 0 | X | X | 0 | 0 | 0 | 0 |
| Bregmatic bone | 0 | | X | | 0 | | 0 | |
| Sagittal ossice | 0 | | X | | 0 | | 0 | |
| Parietal foramen | 0 | 0 | X | X | 1 | X | 0 | 0 |
| Apical Bone | 0 | | 1 | | 0 | | X | |
| Inca bone | 0 | | 0 | | 0 | | X | |
| Lambdoid ossicle | 0 | 0 | 0 | 0 | 1 | 0 | X | X |
| Asterionic bone | 1 | 0 | 1 | X | 0 | 1 | 0 | 0 |
| Occipitomastoid ossicle | 0 | 0 | 0 | X | 0 | 0 | 0 | 0 |
| Parietal notch bone | 0 | 0 | 0 | X | 0 | 0 | 0 | 0 |
| Auditory exostosis | 0 | 0 | 0 | 0 | 0 | 0 | 0 | 0 |
| Mastoid foramen count | 0 | 0 | 1 | 2 | 1 | 2 | 2 | 3 |
| Mastoid foramen location | 0 | 0 | 1 | 1 | 1 | 1 | 1 | 4 |
| Condylar canal | 1 | 2 | X | X | X | X | 1 | 2 |
| Divided hypoglossal canal | 0 | 0 | 0 | X | 0 | 0 | 0 | 0 |
| Tympanic dehiscence | 0 | 0 | 1 | 0 | 0 | 0 | 0 | 0 |
| Foramen spinosum | 0 | 0 | 0 | 0 | 0 | 0 | 0 | 0 |
| Foramen ovale | 0 | 0 | 0 | 0 | 0 | 0 | 0 | 0 |
| Pterygospinous bridge | 0 | X | 3 | 3 | 3 | 3 | 1 | 1 |
| Pterygoalar bridge | 0 | X | X | X | 3 | 3 | X | X |
| Palatine torus development | 0 | | 2 | | 0 | | X | |
| Palatine torus location | 0 | | 3 | | 0 | | X | |
| Mylohyoid bridge developme | 0 | 0 | 0 | 0 | 0 | 0 | 1 | 0 |
| Mylohyoid bridge location | 0 | 0 | 0 | 0 | 0 | 0 | 2 | 0 |
| Mental foramen count | 1 | 1 | 1 | 1 | 0 | 0 | 1 | 1 |
| Mandibular torus | 0 | 0 | 0 | 0 | 0 | 0 | 0 | 0 |
