# Supplementary material for: "The dead shall be raised": Multidisciplinary analysis of human skeletons reveals complexity in 19th century immigrant socioeconomic history and identity in New Haven, Connecticut
Source: PLoS One. 2019 Sep 9;14(9):e0219279. doi: 10.1371/journal.pone.0219279 (PMC6733446; doi:10.1371/journal.pone.0219279)
Supplement: S4 Table — (PDF) [file pone.0219279.s004.pdf]

Supplementary Data Table S4. Craniometric assessment of YNH4 individuals B2 and B3 via FORDISC stepwise weighted method.

YNH4 Individual B2

OBB	BBH	ZYB	ASB	GOL
BPL	PAC	NLH	MAB	ZMB

From Group	Total Number	Into Group BERM	NORM	WM19	WM20	ZALM	Correct
BERM	56	37	13	1	0	5	66.10%
NORM	55	7	26	1	2	19	47.30%
WM19	98	7	5	72	6	8	73.50%
WM20	197	5	7	8	167	10	84.80%
ZALM	53	9	10	6	2	26	49.10%

Total Correct: 328 out of 459 (71.5 %) *** CROSS-VALIDATED ***

Multigroup Classification

Group	Classified into	Distance from	Probabilities Posterior	Typ F	Typ Chi	Typ R
WM19	**WM19**	6	0.857	0.829	0.816	0.939 (6/99)
NORM		11.2	0.063	0.378	0.342	0.143 (48/56)
BERM		11.5	0.055	0.356	0.321	0.333 (38/57)
ZALM		13.5	0.02	0.228	0.197	0.037 (52/54)
WM20		16.4	0.005	0.103	0.088	0.157 (167/198)

YNH4 INDIVIDUAL B2 is closest to WM19s

	YNH4 B2	Chk	Group Means (mm) BERM 56	NORM 55	WM19 98	WM20 197	ZALM 53
OBB	38	-	40.1	40.4	38.1	41.3	40
BBH	128	-	130.3	131.7	133.9	141.2	134.8
ZYB	131		135.6	134.4	132.1	129.5	133
ASB	112		113.6	111.9	111.6	114.6	111
GOL	183		180.3	188.5	183.1	187.7	185.1
BPL	93	-	93.8	97	94.9	98.5	97.1
PAC	114		110.1	114.4	120.4	118.2	115.3
NLH	51	-	51.7	52	52.8	52.8	51.4
MAB	53	--	63.9	63.6	59.6	61.2	64.2
ZMB	86	-	93.3	94	91.2	89.4	94.8

Natural Log of VCVM Determinant = 27.352

YNH4 Individual B3

OBB	BBH	ZMB	FOL
ASB	XCB	GOL	OCC

From Group	Total Number	Into Group BERM	NORM	WM19	WM20	ZALM	Correct
BERM	56	41	6	4	3	2	73.20%
NORM	55	4	32	0	5	14	58.20%
WM19	98	9	11	64	5	9	65.30%
WM20	369	14	25	18	284	28	77.00%
ZALM	53	5	8	9	3	28	52.80%

Total Correct: 449 out of 631 (71.2 %) *** CROSS-VALIDATED ***

Multigroup Classification

Group	Classified into	Distance from	Probabilities Posterior	Typ F	Typ Chi	Typ R
NORM	**NORM**	7.1	0.765	0.545	0.522	0.357 (36/56)
ZALM		11	0.112	0.224	0.202	0.056 (51/54)
WM20		11.6	0.084	0.183	0.172	0.235 (283/370)
BERM		13.3	0.036	0.118	0.103	0.088 (52/57)
WM19		18	0.003	0.026	0.021	0.071 (92/99)

YNH4 INDIVIDUAL B3 is closest to NORMs

	YNH4 B3	Chk	Group Means (mm) BERM 56	NORM 55	WM19 98	WM20 369	ZALM 53
OBB	43	+	40.1	40.4	38.1	41.3	40
BBH	129	-	130.3	131.7	133.9	140.4	134.8
ZMB	91		93.3	94	91.2	89.4	94.8
FOL	38		39	36.5	36.3	37.6	37.2
ASB	110	-	113.6	111.9	111.6	114.5	111
XCB	140	-	147.6	141.9	142.7	140.5	141.4
GOL	192	+	180.3	188.5	183.1	187.2	185.1
OCC	104	+	94	97.3	93.5	100.3	96.2

Natural Log of VCVM Determinant = 21.9739

BERM = Berg, Carinthia, Austria; NORM = Medieval Norse, Oslo, Norway; WM19 = 19th C. Euro-Americans, USA; WM20 = 20th C. Euro-Americans, USA; ZALM = Midieval Zalavar, Hungary. M = Males (all samples).

+/- measurement deviates higher/lower than all group means; ++/-- deviates one to two standard deviations
