# Supplementary material for: "The dead shall be raised": Multidisciplinary analysis of human skeletons reveals complexity in 19th century immigrant socioeconomic history and identity in New Haven, Connecticut
Source: PLoS One. 2019 Sep 9;14(9):e0219279. doi: 10.1371/journal.pone.0219279 (PMC6733446; doi:10.1371/journal.pone.0219279)
Supplement: S5 Table — (PDF) [file pone.0219279.s005.pdf]

Supplementary Table S5. Entheseal Scoring following Mariotti et al. [76, 77]. L=left, R=right. RB=Robusticity, EF=enthesophytic, OL=osteolytic. - = not present; X = feature too damaged to record.

			A						B						B2						B3					
			L			R			L			R			L			R			L			R		
			RB	EF	OL	RB	EF	OL	RB	EF	OL	RB	EF	OL	RB	EF	OL	RB	EF	OL	RB	EF	OL	RB	EF	OL
Forelimb		M. triceps brachii (scapula)	-			X			X			X			-			1C	1	0	3	1	3	2	0	2
Forelimb	Clavicle	Costoclavicular lig.	X			1B	1	2	-			-			-			-			-			-		
Forelimb	Clavicle	Conoid lig.	1	1	0	1C	1	0	X			2	2	3	1C	1	0	1C	1	**2**	2	2	0	1	1	0
Forelimb	Clavicle	Trapezoid lig.	1B	0	1	2	2	0	X			X			1C	1	0	2	2	0	3	3	1	2	1	1
Forelimb	Clavicle	M. pectoralis major	-			2	2	1	1	**1**	0	1	**1**	0	2	1	0	3	2	0	1C	1	2	X		
Forelimb	Clavicle	M. deltoideus	-			-			2	2	1	X			X			1C	1	2	3	3	0	2	2	2
Forelimb	Humerus	M. pectoralis major	X			-			1B	1	2	X			-			X			2	1	0	3	2	0
Forelimb	Humerus	M. lat. dorsii/teres major	X			X			X			1	0	1	-			X			2	1	3	2	1	3
Forelimb	Humerus	M. deltoideus	X			X			X			1	1	0	-			2	1	0	2	2	0	2	1	0
Forelimb	Humerus	M. brachioradialis	-			-			X			X			-			X			2	1	0	X		
Forelimb	Radius	M. biceps brachii	X			X			X			X			-			2	0	1	3	2	3	X		
Forelimb	Radius	M. pronator teres	-			X			1B	0	1	X			-			1A	0	0	X			2	2	1
Forelimb	Radius	Interosseous membrane	-			X			1A	0	0	1A	0	0	1C	1		-			2	3	1	2	2	0
Forelimb	Ulna	M. brachialis	-			-			-			-			2	3	1	-			3	3	1	3	3	2
Forelimb	Ulna	M. triceps brachii	-			-			-			-			X			-			2	1	3	2	1	3
Forelimb	Ulna	M. supinator	2	0	2	-			-			-			3	2	2	-			3	1	2	3	1	3
Hindlimb	Femur	M. gluteus maximus	-						2	1	2	2	1	1	2	2	1	1C	1	1	3	3	1	3	3	2
Hindlimb	Femur	M. iliopsoas							X			X			X			X			2	1	0	2	1	0
Hindlimb	Femur	M. vastus medialis							2	2	1	2	1	1	1C	1	0	1C	1	0	2	1	0	3	2	0
Hindlimb		M. soleus (tibia)							-			3	3	1	2	2	1	2	2	1	3	3	2	3	1	1
Hindlimb		Quadriceps tendon (tibia)							-			2	1	0	X			2	1	0	X			2	1	0
Hindlimb		Quadriceps tendon (patella)							-			X			1B	1	1	1B	1	0	-			2	1	2
Hindlimb		Achilles tendon (calcaneus)							-			X			3	3	0	X			3	2	2	X		
