# Supplementary material for: "The dead shall be raised": Multidisciplinary analysis of human skeletons reveals complexity in 19th century immigrant socioeconomic history and identity in New Haven, Connecticut
Source: PLoS One. 2019 Sep 9;14(9):e0219279. doi: 10.1371/journal.pone.0219279 (PMC6733446; doi:10.1371/journal.pone.0219279)
Supplement: S6 Table — (PDF) [file pone.0219279.s006.pdf]

Supplementary Table S6. Dental calculus weight, DNA yield and microbiome classification and distribution across the YNH4. Taxa sorted by presence/absence in the human oral microbiome and by average frequency across the four individuals.

						YNH.A	YNH.B	YNH.B2	YNH.B3	
					Calculus Weight (mg)	9.7	10.6	10.2	9.0	
					DNA yield (ng DNA / mg sample)	13.7	14.8	40.0	14.5	
Kingdom	Phylum	Class	Order	Family	Genus	DNA yield (ng DNA/mg sample)				Present in human oral microbiome?
Archaea	Euryarchaeota	Methanobacteria	Methanobacteriales	Methanobacteriaceae	*Methanobrevibacter*	3649	2893	671	2873	YES
Bacteria	Firmicutes	Clostridia	Clostridiales	[Mogibacteriaceae]		326	1130	1184	575	YES
Bacteria	TM7	TM7-3				432	961	1399	263	YES
Bacteria	Actinobacteria	Coriobacteriia	Coriobacteriales	Coriobacteriaceae		256	463	276	1407	YES
Bacteria	Chloroflexi	Anaerolineae	Anaerolineales	Anaerolinaceae	*SHD-231*	116	558	763	295	YES
Bacteria	Synergistetes	Synergistia	Synergistales	Dethiosulfovibrionaceae	*TG5*	148	295	979	290	YES
Bacteria	Firmicutes	Clostridia	Clostridiales	[Tissierellaceae]		29	350	491	112	YES
Bacteria	TM7	TM7-3	I025	Rs-045		30	617	33	72	YES
Bacteria	Firmicutes	Clostridia	Clostridiales			87	138	423	98	YES
Bacteria	Firmicutes	Bacilli	Lactobacillales	Streptococcaceae	*Streptococcus*	111	179	385	54	YES
Bacteria	Actinobacteria	Actinobacteria	Actinomycetales	Actinomycetaceae	*Actinomyces*	96	183	262	47	YES
Bacteria	Firmicutes	Clostridia	Clostridiales	Lachnospiraceae		58	119	315	82	YES
Bacteria	Proteobacteria	Epsilonproteobacteria	Campylobacterales	Campylobacteraceae	*Campylobacter*	74	85	220	71	YES
Bacteria	Proteobacteria	Betaproteobacteria	Burkholderiales	Burkholderiaceae	*Lautropia*	181	0	75	5	YES
Bacteria	Bacteroidetes	Flavobacteriia	Flavobacteriales	Flavobacteriaceae	*Capnocytophaga*	42	11	181	19	YES
Bacteria	Proteobacteria	Gammaproteobacteria	Pasteurellales	Pasteurellaceae	*Aggregatibacter*	17	2	186	7	YES
Bacteria	TM7	TM7-3	CW040	F16		19	21	159	8	YES
Bacteria	Firmicutes	Clostridia	Clostridiales	Peptostreptococcaceae		72	13	70	37	YES
Bacteria	TM7	TM7-3	CW040			43	20	122	5	YES
Bacteria	Firmicutes	Clostridia	Clostridiales	[Mogibacteriaceae]	*Mogibacterium*	12	9	102	5	YES
Bacteria	Proteobacteria	Deltaproteobacteria	Desulfobacterales	Desulfobulbaceae	*Desulfobulbus*	12	27	20	62	YES
Bacteria	Firmicutes	Clostridia	Clostridiales	Veillonellaceae	*Selenomonas*	16	28	49	26	YES
Bacteria	Fusobacteria	Fusobacteriia	Fusobacteriales	Leptotrichiaceae	*Leptotrichia*	12	5	86	15	YES
Bacteria	Proteobacteria	Betaproteobacteria	Neisseriales	Neisseriaceae	*Neisseria*	13	1	87	13	YES
Bacteria	Spirochaetes	Spirochaetes	Spirochaetales	Spirochaetaceae	*Treponema*	7	16	81	5	YES
Bacteria	Proteobacteria	Betaproteobacteria	Neisseriales	Neisseriaceae		12	15	67	2	YES
Bacteria	Bacteroidetes	Bacteroidia	Bacteroidales	Porphyromonadaceae	*Tannerella*	26	19	40	7	YES
Bacteria	Firmicutes	Bacilli	Gemellales	Gemellaceae	*Gemella*	26	5	47	8	YES
Bacteria	Firmicutes	Clostridia	Clostridiales	Eubacteriaceae	*Pseudoramibacter_Eubacterium*	4	0	69	7	YES
Bacteria	Bacteroidetes	Bacteroidia	Bacteroidales	Porphyromonadaceae	*Paludibacter*	22	6	37	12	YES
Bacteria	Fusobacteria	Fusobacteriia	Fusobacteriales	Fusobacteriaceae	*Fusobacterium*	3	4	57	2	YES
Bacteria	Firmicutes	Clostridia	Clostridiales	[Tissierellaceae]	*Parvimonas*	12	7	41	4	NO
Bacteria	Actinobacteria	Actinobacteria	Actinomycetales	Micrococcaceae	*Rothia*	21	1	3	26	YES
Bacteria	Bacteroidetes	Bacteroidia	Bacteroidales	Porphyromonadaceae	*Porphyromonas*	3	1	36	0	YES
Bacteria	Firmicutes	Clostridia	Clostridiales	Peptostreptococcaceae	*Filifactor*	5	2	32	1	YES
Bacteria	Proteobacteria	Betaproteobacteria	Neisseriales	Neisseriaceae	*Eikenella*	3	4	25	2	YES
Bacteria	Proteobacteria	Gammaproteobacteria	Cardiobacteriales	Cardiobacteriaceae	*Cardiobacterium*	1	1	26	4	YES
Bacteria	Bacteroidetes	Bacteroidia	Bacteroidales	Prevotellaceae	*Prevotella*	5	1	21	1	YES
Bacteria	Actinobacteria	Coriobacteriia	Coriobacteriales	Coriobacteriaceae	*Atopobium*	4	3	7	14	YES
Bacteria	Firmicutes	Clostridia	Clostridiales	Lachnospiraceae	*Catonella*	14	0	6	6	YES
Bacteria	Firmicutes	Clostridia	Clostridiales	Lachnospiraceae	*Butyrivibrio*	2	5	15	2	YES
Bacteria	Firmicutes	Clostridia	Clostridiales	Peptococcaceae	*Peptococcus*	2	4	14	4	YES
Bacteria	Firmicutes	Bacilli	Lactobacillales	Carnobacteriaceae	*Granulicatella*	12	0	5	0	YES
Bacteria	Firmicutes	Clostridia	Clostridiales	[Tissierellaceae]	*Peptoniphilus*	2	4	9	0	YES
Bacteria	Actinobacteria	Actinobacteria	Actinomycetales	Corynebacteriaceae	*Corynebacterium*	0	0	3	10	YES
Bacteria	Proteobacteria	Gammaproteobacteria	Pasteurellales	Pasteurellaceae	*Haemophilus*	4	0	3	1	YES
Bacteria	Actinobacteria	Coriobacteriia	Coriobacteriales	Coriobacteriaceae	*Slackia*	0	5	1	2	YES
Bacteria	Firmicutes	Clostridia	Clostridiales	Lachnospiraceae	*Oribacterium*	3	0	2	3	YES
Bacteria	Firmicutes	Erysipelotrichi	Erysipelotrichales	Erysipelotrichaceae	*Bulleidia*	1	1	5	0	YES
Bacteria	Firmicutes	Clostridia	Clostridiales	Veillonellaceae	*Dialister*	4	1	2	0	YES
Bacteria	Firmicutes	Clostridia	Clostridiales	Veillonellaceae	*Veillonella*	4	2	0	1	YES
Bacteria	Actinobacteria	Actinobacteria	Actinomycetales	Propionibacteriaceae	*Propionibacterium*	0	3	0	4	YES
Bacteria	Proteobacteria	Deltaproteobacteria	Desulfovibrionales	Desulfovibrionaceae	*Desulfovibrio*	0	4	0	0	YES
Bacteria	Firmicutes	Bacilli	Bacillales	Paenibacillaceae	*Paenibacillus*	1	3	0	0	YES
Bacteria	Actinobacteria	Actinobacteria	Bifidobacteriales	Bifidobacteriaceae	*Bifidobacterium*	0	0	0	3	YES
Bacteria	Proteobacteria	Betaproteobacteria	Burkholderiales	Oxalobacteraceae	*Ralstonia*	0	0	0	3	YES
Bacteria	Firmicutes	Bacilli	Lactobacillales	Aerococcaceae	*Abiotrophia*	1	0	1	0	YES
Bacteria	Proteobacteria	Gammaproteobacteria	Pseudomonadales	Moraxellaceae	*Moraxella*	1	0	1	0	YES
Bacteria	Firmicutes	Clostridia	Clostridiales	Peptostreptococcaceae	*Peptostreptococcus*	1	0	1	0	YES
Bacteria	Actinobacteria	Actinobacteria	Actinomycetales	Micrococcaceae	*Kocuria*	0	0	0	2	YES
Bacteria	Firmicutes	Bacilli	Bacillales	Bacillaceae	*Bacillus*	0	0	0	1	YES
Bacteria	Bacteroidetes	Bacteroidia	Bacteroidales	Bacteroidaceae	*Bacteroides*	0	0	0	1	YES
Bacteria	Proteobacteria	Betaproteobacteria	Neisseriales	Neisseriaceae	*Kingella*	0	0	0	1	YES
Bacteria	Firmicutes	Clostridia	Clostridiales	Veillonellaceae	*Megasphaera*	1	0	0	0	YES
Bacteria	Actinobacteria	Actinobacteria	Actinomycetales	Pseudonocardiaceae		982	58	1	9	NO
Bacteria	Proteobacteria	Alphaproteobacteria	Rhodospirillales	Rhodospirillaceae		184	102	29	412	NO
Bacteria	Actinobacteria	Actinobacteria	Actinomycetales	Frankiaceae		0	4	14	539	NO
Bacteria	Proteobacteria	Alphaproteobacteria	Rhizobiales	Hyphomicrobiaceae	*Pedomicrobium*	45	121	13	378	NO
Bacteria	Acidobacteria	Acidobacteria-6	iii1-15			214	43	0	73	NO
Bacteria	Proteobacteria	Betaproteobacteria	MND1			188	62	14	61	NO
Bacteria	Gemmatimonadetes	Gemm-1				93	26	9	99	NO
Bacteria	Proteobacteria	Alphaproteobacteria	Rhizobiales			63	41	7	111	NO
Bacteria	Nitrospirae	Nitrospira	Nitrospirales	Nitrospiraceae		85	94	20	22	NO
Bacteria	Proteobacteria	Alphaproteobacteria	Rhodobacterales	Hyphomonadaceae		16	25	6	172	NO
Bacteria	Actinobacteria	Thermoleophilia	Solirubrobacterales			7	5	12	187	NO
Bacteria	Proteobacteria	Alphaproteobacteria	Rhizobiales	Hyphomicrobiaceae		98	8	2	101	NO

Supplementary Table S6. Dental calculus weight, DNA yield and microbiome classification and distribution across the YNH4. Taxa sorted by presence/absence in the human oral microbiome and by average frequency across the four individuals.

						YNH.A	YNH.B	YNH.B2	YNH.B3	
					Calculus Weight (mg)	9.7	10.6	10.2	9.0	
					DNA yield (ng DNA / mg sample)	13.7	14.8	40.0	14.5	
Kingdom	Phylum	Class	Order	Family	Genus	DNA yield (ng DNA/mg sample)				Present in human oral microbiome?
Bacteria	Chloroflexi	S085				125	13	0	66	NO
Bacteria	Proteobacteria	Betaproteobacteria	Burkholderiales	Comamonadaceae		48	8	116	30	NO
Bacteria	Nitrospirae	Nitrospira	Nitrospirales	0319-6A21		142	38	7	7	NO
Bacteria	Proteobacteria	Alphaproteobacteria	Rhizobiales	Hyphomicrobiaceae	*Rhodoplanes*	50	22	6	115	NO
Bacteria	Proteobacteria	Betaproteobacteria				134	14	4	37	NO
Bacteria	Firmicutes	Clostridia	Clostridiales	[Tissierellaceae]	*Sporanaerobacter*	83	3	54	48	NO
Bacteria	Synergistetes	Synergistia	Synergistales	Synergistaceae	*Candidatus Tammella*	24	83	28	11	NO
Bacteria	Proteobacteria	Alphaproteobacteria				54	13	2	74	NO
Bacteria	Proteobacteria	Deltaproteobacteria	Syntrophobacterales	Syntrophobacteraceae		56	21	3	50	NO
Bacteria	Proteobacteria	Gammaproteobacteria	Xanthomonadales	Sinobacteraceae		33	65	5	13	NO
Bacteria	Chloroflexi	Anaerolineae	SBR1031	A4b		41	75	0	0	NO
Bacteria	Actinobacteria	Acidimicrobiia	Acidimicrobiales	AKIW874		91	15	0	1	NO
Bacteria	Acidobacteria	DA052	Ellin6513			72	19	7	6	NO
Bacteria	Firmicutes	Clostridia	Clostridiales	Veillonellaceae	*Schwartzia*	13	24	41	23	NO
Bacteria	Acidobacteria	Solibacteres	Solibacterales			24	22	2	44	NO
Bacteria	Actinobacteria	Actinobacteria	Actinomycetales			7	31	1	48	NO
Bacteria	Proteobacteria	Alphaproteobacteria	Rhizobiales	Xanthobacteraceae		21	24	2	39	NO
Bacteria	Acidobacteria	Acidobacteria-6	iii1-15	mb2424		32	34	4	15	NO
Bacteria	Proteobacteria	Betaproteobacteria	IS-44			45	27	3	3	NO
Bacteria	Chloroflexi	Anaerolineae	GCA004			0	74	0	0	NO
Bacteria	Proteobacteria	Gammaproteobacteria	Xanthomonadales	Sinobacteraceae	*Steroidobacter*	0	58	3	5	NO
Bacteria	Acidobacteria	[Chloracidobacteria]	11-24			55	8	0	0	NO
Bacteria	Proteobacteria	Deltaproteobacteria	Desulfovibrionales	Desulfomicrobiaceae	*Desulfomicrobium*	2	0	55	1	NO
Bacteria	Acidobacteria	Acidobacteria-6	CCU21			42	4	1	10	NO
Bacteria	Bacteroidetes	Cytophagia	Cytophagales	Cytophagaceae		40	17	0	0	NO
Bacteria	Proteobacteria	Alphaproteobacteria	Rhizobiales	Bradyrhizobiaceae	*Bradyrhizobium*	44	7	0	5	NO
Bacteria	Firmicutes	Clostridia	Clostridiales	[Acidaminobacteraceae]		4	18	27	6	NO
Bacteria	Actinobacteria	Acidimicrobiia	Acidimicrobiales	EB1017		23	7	2	22	NO
Bacteria	Bacteroidetes	[Saprospirae]	[Saprospirales]	Chitinophagaceae		8	4	1	41	NO
Bacteria	Nitrospirae	Nitrospira	Nitrospirales	Nitrospiraceae	*Nitrospira*	20	12	0	22	NO
Bacteria	Bacteroidetes	Flavobacteriia	Flavobacteriales	[Weeksellaceae]		4	1	45	3	NO
Bacteria	Actinobacteria	Thermoleophilia	Gaiellales	Gaiellaceae		27	3	0	22	NO
Bacteria	Acidobacteria	[Chloracidobacteria]	RB41	Ellin6075		3	33	1	11	NO
Bacteria	Firmicutes	Bacilli	Lactobacillales	Aerococcaceae		26	1	18	1	NO
Bacteria	Bacteroidetes	Bacteroidia	Bacteroidales			3	8	16	19	NO
Bacteria	Actinobacteria	Actinobacteria	Actinomycetales	Streptosporangiaceae	*Nonomuraea*	0	46	0	0	NO
Bacteria	Elusimicrobia	Endomicrobia				12	2	24	4	NO
Bacteria	Actinobacteria	Actinobacteria	Actinomycetales	Propionibacteriaceae		6	6	13	14	NO
Bacteria	Proteobacteria	Alphaproteobacteria	Rhizobiales	Hyphomicrobiaceae	*Hyphomicrobium*	18	4	3	14	NO
Bacteria	GN02	BD1-5				12	1	21	2	NO
Bacteria	Actinobacteria	Actinobacteria	Actinomycetales	Streptomycetaceae	*Streptomyces*	8	1	8	19	NO
Bacteria	Firmicutes	Clostridia	Clostridiales	Lachnospiraceae	*Moryella*	1	1	19	14	NO
Bacteria	Chloroflexi	Ellin6529				24	8	0	1	NO
Bacteria	Firmicutes	Clostridia	Clostridiales	Clostridiaceae	*Clostridium*	13	3	1	15	NO
Bacteria	Firmicutes	Clostridia	Clostridiales	Syntrophomonadaceae	*Syntrophomonas*	0	3	16	11	NO
Bacteria	Proteobacteria	Alphaproteobacteria	Ellin329			5	3	1	20	NO
Bacteria	Acidobacteria	Acidobacteriia	Acidobacteriales	Koribacteraceae		6	1	0	22	NO
Bacteria	Proteobacteria	Betaproteobacteria	Ellin6067			1	23	4	0	NO
Bacteria	Acidobacteria	Solibacteres	Solibacterales	PAUC26f		12	12	0	4	NO
Bacteria	Actinobacteria	Actinobacteria	Actinomycetales	Frankiaceae	*Frankia*	0	22	1	4	NO
Bacteria	Actinobacteria	Actinobacteria	Actinomycetales	Nocardiaceae	*Rhodococcus*	2	1	0	24	NO
Bacteria	Acidobacteria	Acidobacteria-5				16	7	0	4	NO
Bacteria	Proteobacteria	Alphaproteobacteria	Rhizobiales	Methylocystaceae		1	0	1	23	NO
Bacteria	Acidobacteria	Solibacteres	Solibacterales	Solibacteraceae	*Candidatus Solibacter*	3	0	0	22	NO
Bacteria	Acidobacteria	Solibacteres	Solibacterales	AKIW659		12	5	6	0	NO
Bacteria	Proteobacteria	Gammaproteobacteria	Pseudomonadales	Pseudomonadaceae	*Pseudomonas*	17	2	3	0	NO
Bacteria	Acidobacteria	[Chloracidobacteria]	RB41			6	5	0	11	NO
Bacteria	Armatimonadetes	[Fimbriimonadia]	[Fimbriimonadales]			0	15	0	7	NO
Bacteria	Gemmatimonadetes	Gemmatimonadetes	KD8-87			14	6	0	2	NO
Bacteria	Chloroflexi	Anaerolineae	envOPS12			11	10	0	0	NO
Bacteria	TM7	TM7-3	EW055			3	4	13	0	NO
Bacteria	Actinobacteria	Acidimicrobiia	Acidimicrobiales			12	5	0	2	NO
Bacteria	Gemmatimonadetes	Gemmatimonadetes				19	0	0	0	NO
Bacteria	Proteobacteria	Alphaproteobacteria	Rhizobiales	Bradyrhizobiaceae		8	6	0	4	NO
Bacteria	Proteobacteria	Deltaproteobacteria	Myxococcales			8	4	1	4	NO
Bacteria	Actinobacteria	Actinobacteria	Actinomycetales	Mycobacteriaceae	*Mycobacterium*	1	7	0	8	NO
Bacteria	SR1					1	1	12	1	NO
Bacteria	TM6	SJA-4				1	3	11	0	NO
Bacteria	Actinobacteria	Actinobacteria	Actinomycetales	Frankiaceae	*Kaistibacter*	0	0	0	15	NO
Bacteria	Proteobacteria					11	4	0	0	NO
Bacteria	Armatimonadetes	0319-6E2				2	12	0	0	NO
Bacteria	Firmicutes	Clostridia	Clostridiales	[Acidaminobacteraceae]	*Fusibacter*	6	0	6	1	NO
Bacteria	Proteobacteria	Alphaproteobacteria	Sphingomonadales	Sphingomonadaceae	*Sphingomonas*	0	11	1	1	NO
Bacteria	Acidobacteria	Sva0725	Sva0725			12	1	0	0	NO
Bacteria	Proteobacteria	Betaproteobacteria	Rhodocyclales	Rhodocyclaceae	*Propionivibrio*	2	1	9	0	NO
Bacteria	Firmicutes	Clostridia	Clostridiales	Ruminococcaceae		1	2	2	7	NO

Supplementary Table S6. Dental calculus weight, DNA yield and microbiome classification and distribution across the YNH4. Taxa sorted by presence/absence in the human oral microbiome and by average frequency across the four individuals.

						YNH.A	YNH.B	YNH.B2	YNH.B3	
				Calculus Weight (mg)		9.7	10.6	10.2	9.0	
				DNA yield (ng DNA / mg sample)		13.7	14.8	40.0	14.5	
Kingdom	Phylum	Class	Order	Family	Genus	DNA yield (ng DNA/mg sample)				Present in human oral microbiome?
Bacteria	Armatimonadetes	[Fimbriimonadia]	[Fimbriimonadales]	[Fimbriimonadaceae]		1	9	1	1	NO
Bacteria	Chloroflexi	TK10	B07_WMSP1			3	2	1	6	NO
Bacteria	Proteobacteria	Alphaproteobacteria	Rhizobiales	Rhodobiaceae	*Afifella*	3	3	0	6	NO
Archaea	Crenarchaeota	Thaumarchaeota	Cenarchaeales	Cenarchaeaceae		7	5	0	0	NO
Bacteria	Synergistetes	Synergistia	Synergistales	Dethiosulfovibrionaceae	*Pyramidobacter*	2	2	2	5	NO
Bacteria	Chloroflexi	Anaerolineae	H39			8	3	0	0	NO
Bacteria	Chloroflexi	Anaerolineae	S0208			11	0	0	0	NO
Bacteria	Chloroflexi	Thermomicrobia	JG30-KF-CM45			1	3	0	7	NO
Bacteria	Proteobacteria	Alphaproteobacteria	Rhodospirillales			0	9	0	2	NO
Bacteria	Firmicutes	Clostridia	Clostridiales	Ruminococcaceae	*Oscillospira*	1	0	0	9	NO
Bacteria	Chloroflexi	Anaerolineae	A31	S47		6	4	0	0	NO
Bacteria	Chloroflexi	TK17	mle1-48			5	0	0	5	NO
Bacteria	Planctomycetes	Planctomycetia	Gemmatales	Gemmataceae		4	2	0	4	NO
Bacteria	Firmicutes	Clostridia	Clostridiales	Clostridiaceae		6	1	2	0	NO
Bacteria	Cyanobacteria	4C0d-2	MLE1-12			6	2	1	0	NO
Bacteria	Proteobacteria	Alphaproteobacteria	Rhizobiales	Beijerinckiaceae		2	0	1	6	NO
Bacteria	Proteobacteria	Alphaproteobacteria	Rhodospirillales	Rhodospirillaceae	*Magnetospirillum*	2	4	0	3	NO
Bacteria	Actinobacteria	MB-A2-108	0319-7L14			7	2	0	0	NO
Bacteria	Firmicutes	Bacilli	Gemellales	Gemellaceae		9	0	0	0	NO
Bacteria	Proteobacteria	Deltaproteobacteria	Myxococcales	Haliangiaceae		3	0	0	6	NO
Bacteria	Proteobacteria	Gammaproteobacteria	Xanthomonadales			9	0	0	0	NO
Bacteria	Firmicutes	Clostridia	Clostridiales	[Mogibacteriaceae]	*Anaerovorax*	1	0	6	1	NO
Bacteria	Gemmatimonadetes	Gemmatimonadetes	Ellin5290			4	1	1	2	NO
Bacteria	Acidobacteria	Acidobacteria-6	iii1-15	RB40		2	1	0	5	NO
Bacteria	Actinobacteria	Actinobacteria	Actinomycetales	Micromonosporaceae		7	1	0	0	NO
Bacteria	Chloroflexi	TK17				3	1	0	4	NO
Bacteria	Actinobacteria	Acidimicrobiia	Acidimicrobiales	C111		0	0	1	6	NO
Bacteria	Firmicutes	Clostridia	Clostridiales	Veillonellaceae		0	2	1	4	NO
Bacteria	Actinobacteria	Actinobacteria	Actinomycetales	Propionibacteriaceae	*Brooklawnia*	2	0	0	5	NO
Bacteria	Chloroflexi	Gitt-GS-136				7	0	0	0	NO
Bacteria	Planctomycetes	Phycisphaerae	Phycisphaerales			6	1	0	0	NO
Bacteria	Proteobacteria	Betaproteobacteria	SBla14			7	0	0	0	NO
Bacteria	WS2	SHA-109				7	0	0	0	NO
Bacteria	Proteobacteria	Alphaproteobacteria	Rhodobacterales	Rhodobacteraceae		0	0	2	4	NO
Bacteria	Proteobacteria	Alphaproteobacteria	Rhizobiales	Phyllobacteriaceae	*Mesorhizobium*	0	3	1	2	NO
Bacteria	Actinobacteria	Actinobacteria	Actinomycetales	Nocardiaceae	*Nocardia*	1	5	0	0	NO
Bacteria	Actinobacteria	Actinobacteria	Actinomycetales	Nocardiaceae		0	0	0	6	NO
Bacteria	Chloroflexi	Anaerolineae	Caldilineales	Caldilineaceae		2	4	0	0	NO
Bacteria	Firmicutes	Bacilli	Bacillales	Bacillaceae		6	0	0	0	NO
Bacteria	Proteobacteria	Gammaproteobacteria				0	0	0	6	NO
Bacteria	Verrucomicrobia	[Pedosphaerae]	[Pedosphaerales]			6	0	0	0	NO
Bacteria	Firmicutes	Clostridia	Clostridiales	[Tissierellaceae]	*ph2*	1	0	4	0	NO
Bacteria	Synergistetes	Synergistia	Synergistales	Synergistaceae		0	0	4	1	NO
Archaea	Crenarchaeota	Thaumarchaeota	Nitrososphaerales	Nitrososphaeraceae	*Candidatus Nitrososphaera*	4	0	1	0	NO
Bacteria	Acidobacteria	Acidobacteriia	Acidobacteriales	Koribacteraceae	*Candidatus Koribacter*	3	2	0	0	NO
Bacteria	Chloroflexi	TK10	AKYG885	Dolo_23		3	1	0	1	NO
Bacteria	GAL15					2	3	0	0	NO
Bacteria	Proteobacteria	Alphaproteobacteria	Rhodospirillales	Acetobacteraceae		2	1	0	2	NO
Bacteria	Proteobacteria	Gammaproteobacteria	Chromatiales			5	0	0	0	NO
Bacteria	Proteobacteria	Gammaproteobacteria	Legionellales	Coxiellaceae		2	2	0	1	NO
Bacteria	WS3	PRR-12	Sediment-1	CV106		5	0	0	0	NO
Bacteria	Acidobacteria	[Chloracidobacteria]	PK29			3	0	1	0	NO
Bacteria	Acidobacteria	BPC102				2	1	1	0	NO
Bacteria	Proteobacteria	Deltaproteobacteria	Bdellovibrionales	Bdellovibrionaceae	*Bdellovibrio*	0	3	0	1	NO
Bacteria	Actinobacteria	KIST-JJY010				4	0	0	0	NO
Bacteria	Gemmatimonadetes	Gemmatimonadetes	C114			4	0	0	0	NO
Bacteria	Proteobacteria	Alphaproteobacteria	Rhizobiales	Phyllobacteriaceae		0	2	0	2	NO
Bacteria	Proteobacteria	Alphaproteobacteria	Rickettsiales			1	1	0	2	NO
Bacteria	Proteobacteria	Deltaproteobacteria	MIZ46			3	0	0	1	NO
Bacteria	Proteobacteria	Gammaproteobacteria	Pseudomonadales	Pseudomonadaceae		4	0	0	0	NO
Bacteria	SBR1093	VHS-B5-50				3	1	0	0	NO
Bacteria	WPS-2					0	0	0	4	NO
Bacteria	Bacteroidetes	[Saprospirae]	[Saprospirales]	Chitinophagaceae	*Chitinophaga*	0	3	0	0	NO
Bacteria	Firmicutes	Clostridia	Clostridiales	Christensenellaceae	*Christensenella*	0	2	0	1	NO
Bacteria	Verrucomicrobia	[Spartobacteria]	[Chthoniobacterales]	[Chthoniobacteraceae]	*DA101*	0	1	0	2	NO
Bacteria	Proteobacteria	Alphaproteobacteria	Rhizobiales	Hyphomicrobiaceae	*Devosia*	1	2	0	0	NO
Archaea	Crenarchaeota	Thaumarchaeota	Cenarchaeales	Cenarchaeaceae	*Nitrosopumilus*	3	0	0	0	NO
Bacteria	Actinobacteria	Actinobacteria	Actinomycetales	Micromonosporaceae	*Pilimelia*	0	0	0	3	NO
Bacteria	Acidobacteria	EC1113				2	1	0	0	NO
Bacteria	Acidobacteria	Solibacteres	JH-WHS99			0	3	0	0	NO
Bacteria	Actinobacteria	Actinobacteria	Actinomycetales	Geodermatophilaceae		0	2	0	1	NO
Bacteria	Actinobacteria	Actinobacteria	Actinomycetales	Nocardioidaceae		2	1	0	0	NO
Bacteria	Chlorobi	SJA-28				3	0	0	0	NO
Bacteria	Cyanobacteria	Chloroplast	Streptophyta			3	0	0	0	NO
Bacteria	Gemmatimonadetes	Gemmatimonadetes	N1423WL			0	0	0	3	NO
Bacteria	Planctomycetes	Planctomycetia	Gemmatales	Isosphaeraceae		1	1	0	1	NO

Supplementary Table S6. Dental calculus weight, DNA yield and microbiome classification and distribution across the YNH4. Taxa sorted by presence/absence in the human oral microbiome and by average frequency across the four individuals.

						YNH.A	YNH.B	YNH.B2	YNH.B3	
				Calculus Weight (mg)		9.7	10.6	10.2	9.0	
				DNA yield (ng DNA / mg sample)		13.7	14.8	40.0	14.5	
Kingdom	Phylum	Class	Order	Family	Genus	DNA yield (ng DNA/mg sample)				Present in human oral microbiome?
Bacteria	Proteobacteria	Alphaproteobacteria	Kiloniellales			1	0	0	2	NO
Bacteria	Proteobacteria	Deltaproteobacteria	Myxococcales	Polyangiaceae		3	0	0	0	NO
Bacteria	Firmicutes	Clostridia	Clostridiales	Lachnospiraceae	*[Ruminococcus]*	0	0	2	0	NO
Bacteria	Chlorobi	OPB56				0	0	2	0	NO
Bacteria	Actinobacteria	Thermoleophilia	Solirubrobacterales	Solirubrobacteraceae		1	0	1	0	NO
Bacteria	Proteobacteria	Gammaproteobacteria	Legionellales	Coxiellaceae	*Aquicella*	1	1	0	0	NO
Bacteria	Actinobacteria	Actinobacteria	Actinomycetales	Micrococcaceae	*Arthrobacter*	2	0	0	0	NO
Bacteria	Actinobacteria	Actinobacteria	Actinomycetales	Geodermatophilaceae	*Blastococcus*	0	0	0	2	NO
Bacteria	Firmicutes	Clostridia	Clostridiales	Clostridiaceae	*Caldanaerocella*	2	0	0	0	NO
Bacteria	Verrucomicrobia	[Spartobacteria]	[Chthoniobacterales]	[Chthoniobacteraceae]	*Candidatus Xiphinematobacter*	0	1	0	1	NO
Bacteria	Firmicutes	Clostridia	Clostridiales	Clostridiaceae	*Clostridiisalibacter*	2	0	0	0	NO
Bacteria	Proteobacteria	Gammaproteobacteria	Xanthomonadales	Xanthomonadaceae	*Dokdonella*	0	0	0	2	NO
Bacteria	Armatimonadetes	[Fimbriimonadia]	[Fimbriimonadales]	[Fimbriimonadaceae]	*Fimbriimonas*	0	0	0	2	NO
Bacteria	Actinobacteria	Actinobacteria	Actinomycetales	Nocardioidaceae	*Kribbella*	1	1	0	0	NO
Bacteria	Proteobacteria	Betaproteobacteria	Burkholderiales	Comamonadaceae	*Methylibium*	2	0	0	0	NO
Bacteria	Proteobacteria	Alphaproteobacteria	Rhizobiales	Phyllobacteriaceae	*Thermovum*	1	0	0	1	NO
Bacteria						2	0	0	0	NO
Bacteria	Acidobacteria	[Chloracidobacteria]	Ellin7246			0	0	0	2	NO
Bacteria	Acidobacteria	PAUC37f				0	2	0	0	NO
Bacteria	Actinobacteria	Actinobacteria	Actinomycetales	Actinomycetaceae		0	1	0	1	NO
Bacteria	Actinobacteria	Actinobacteria	Actinomycetales	Streptomycetaceae		2	0	0	0	NO
Bacteria	Actinobacteria	Actinobacteria	Actinomycetales	Streptosporangiaceae		0	2	0	0	NO
Bacteria	Actinobacteria	Thermoleophilia	Gaiellales			0	2	0	0	NO
Bacteria	Actinobacteria	Thermoleophilia	Solirubrobacterales	Conexibacteraceae		2	0	0	0	NO
Bacteria	BRC1	PRR-11				0	0	0	2	NO
Bacteria	Chlorobi					0	2	0	0	NO
Bacteria	Chloroflexi	TK10	AKYG885	5B-12		2	0	0	0	NO
Bacteria	Gemmatimonadetes	Gemm-2				2	0	0	0	NO
Bacteria	PAUC34f					2	0	0	0	NO
Bacteria	Planctomycetes	Phycisphaerae	WD2101			2	0	0	0	NO
Bacteria	Proteobacteria	Alphaproteobacteria	Sphingomonadales	Sphingomonadaceae		0	1	0	1	NO
Bacteria	Proteobacteria	Betaproteobacteria	Burkholderiales	Alcaligenaceae		1	1	0	0	NO
Bacteria	Proteobacteria	Betaproteobacteria	SC-I-84			1	0	0	1	NO
Bacteria	Proteobacteria	Gammaproteobacteria	Legionellales	Legionellaceae		0	1	0	1	NO
Bacteria	TM7	MJK10				2	0	0	0	NO
Bacteria	TM7	TM7-1				1	1	0	0	NO
Bacteria	WS3	PRR-12	Sediment-1			2	0	0	0	NO
Bacteria	Bacteroidetes	Flavobacteriia	Flavobacteriales	[Weeksellaceae]	*Chryseobacterium*	0	0	1	0	NO
Bacteria	Firmicutes	Clostridia	Clostridiales	Lachnospiraceae	*Epulopiscium*	0	0	1	0	NO
Bacteria	Proteobacteria	Epsilonproteobacteria	Campylobacterales	Helicobacteraceae	*Flexispira*	0	0	1	0	NO
Bacteria	Bacteroidetes	[Rhodothermi]	[Rhodothermales]	[Balneolaceae]	*KSA1*	0	0	1	0	NO
Bacteria	Proteobacteria	Betaproteobacteria	Burkholderiales	Comamonadaceae	*Lampropedia*	0	0	1	0	NO
Bacteria	Fusobacteria	Fusobacteriia	Fusobacteriales	Fusobacteriaceae	*Propionigenium*	0	0	1	0	NO
Bacteria	Firmicutes	Clostridia	Clostridiales	[Tissierellaceae]	*Tepidimicrobium*	0	0	1	0	NO
Bacteria	Bacteroidetes	Bacteroidia	Bacteroidales	BS11		0	0	1	0	NO
Bacteria	Chloroflexi	Thermomicrobia	AKYG1722			0	0	1	0	NO
Bacteria	Firmicutes	Bacilli	Bacillales			0	0	1	0	NO
Bacteria	Fusobacteria	Fusobacteriia	Fusobacteriales			0	0	1	0	NO
Bacteria	Proteobacteria	Deltaproteobacteria	Desulfovibrionales	Desulfomicrobiaceae		0	0	1	0	NO
Bacteria	Bacteroidetes	Bacteroidia	Bacteroidales	[Paraprevotellaceae]	*[Prevotella]*	0	0	0	1	NO
Bacteria	Actinobacteria	Actinobacteria	Actinomycetales	Thermomonosporaceae	*Actinoallomurus*	0	0	0	1	NO
Bacteria	Firmicutes	Clostridia	Clostridiales	Clostridiaceae	*Alkaliphilus*	0	0	0	1	NO
Bacteria	Proteobacteria	Alphaproteobacteria	Rhizobiales	Phyllobacteriaceae	*Aminobacter*	1	0	0	0	NO
Bacteria	Actinobacteria	Actinobacteria	Actinomycetales	Pseudonocardiaceae	*Amycolatopsis*	1	0	0	0	NO
Bacteria	Proteobacteria	Alphaproteobacteria	Rhodospirillales	Rhodospirillaceae	*Azospirillum*	0	0	0	1	NO
Bacteria	Firmicutes	Bacilli	Bacillales	Paenibacillaceae	*Brevibacillus*	0	1	0	0	NO
Bacteria	Proteobacteria	Alphaproteobacteria	Rhizobiales	Rhizobiaceae	*Candidatus Liberibacter*	1	0	0	0	NO
Bacteria	Actinobacteria	Actinobacteria	Actinomycetales	Micromonosporaceae	*Catellatospora*	1	0	0	0	NO
Bacteria	Firmicutes	Clostridia	Clostridiales	Lachnospiraceae	*Coprococcus*	0	0	0	1	NO
Bacteria	Firmicutes	Clostridia	Clostridiales	Peptococcaceae	*Desulfotomaculum*	0	1	0	0	NO
Bacteria	Actinobacteria	Actinobacteria	Actinomycetales	Dietziaceae	*Dietzia*	0	0	0	1	NO
Bacteria	Actinobacteria	Actinobacteria	Actinomycetales	Geodermatophilaceae	*Geodermatophilus*	0	0	0	1	NO
Bacteria	Proteobacteria	Alphaproteobacteria	Rickettsiales	mitochondria	*Grevillea*	0	0	0	1	NO
Bacteria	Proteobacteria	Alphaproteobacteria	Rhodospirillales	Rhodospirillaceae	*Inquilinus*	1	0	0	0	NO
Bacteria	Proteobacteria	Alphaproteobacteria	Sphingomonadales	Sphingomonadaceae	*Kaistobacter*	0	0	0	1	NO
Bacteria	Actinobacteria	Actinobacteria	Actinomycetales	Kineosporiaceae	*Kineosporia*	0	0	0	1	NO
Bacteria	Actinobacteria	Actinobacteria	Actinomycetales	Streptomycetaceae	*Kitasatospora*	0	0	0	1	NO
Bacteria	Proteobacteria	Gammaproteobacteria	Legionellales	Legionellaceae	*Legionella*	0	0	0	1	NO
Bacteria	Proteobacteria	Betaproteobacteria	Burkholderiales	Comamonadaceae	*Limnohabitans*	1	0	0	0	NO
Bacteria	Proteobacteria	Betaproteobacteria	Burkholderiales	Oxalobacteraceae	*Massilia*	0	1	0	0	NO
Bacteria	Proteobacteria	Alphaproteobacteria	Rhodospirillales	Rhodospirillaceae	*Novispirillum*	1	0	0	0	NO
Bacteria	Proteobacteria	Alphaproteobacteria	Rhizobiales	Brucellaceae	*Ochrobactrum*	0	0	0	1	NO
Bacteria	Verrucomicrobia	Opitutae	Opitutales	Opitutaceae	*Opitutus*	0	0	0	1	NO
Bacteria	Proteobacteria	Alphaproteobacteria	Rhizobiales	Hyphomicrobiaceae	*Parvibaculum*	0	0	0	1	NO
Bacteria	Planctomycetes	Planctomycetia	Planctomycetales	Planctomycetaceae	*Planctomyces*	0	1	0	0	NO
Bacteria	Actinobacteria	Actinobacteria	Actinomycetales	Pseudonocardiaceae	*Pseudonocardia*	0	0	0	1	NO

Supplementary Table S6. Dental calculus weight, DNA yield and microbiome classification and distribution across the YNH4. Taxa sorted by presence/absence in the human oral microbiome and by average frequency across the four individuals.

Kingdom	Phylum	Class	Order	Family	Genus	YNH.A	YNH.B	YNH.B2	YNH.B3	Present in human oral microbiome?
					Calculus Weight (mg)	9.7	10.6	10.2	9.0	
					DNA yield (ng DNA / mg sample)	13.7	14.8	40.0	14.5	
						DNA yield (ng DNA/mg sample)				
Bacteria	Proteobacteria	Alphaproteobacteria	Rhodospirillales	Rhodospirillaceae	*Rhodovibrio*	1	0	0	0	NO
Bacteria	Firmicutes	Clostridia	Clostridiales	Lachnospiraceae	*Roseburia*	1	0	0	0	NO
Bacteria	Actinobacteria	Actinobacteria	Actinomycetales	Microbacteriaceae	*Salinibacterium*	0	0	0	1	NO
Bacteria	Actinobacteria	Actinobacteria	Actinomycetales	Intrasporangiaceae	*Serinicoccus*	0	1	0	0	NO
Bacteria	Firmicutes	Erysipelotrichi	Erysipelotrichales	Erysipelotrichaceae	*Sharpea*	0	0	0	1	NO
Bacteria	Proteobacteria	Deltaproteobacteria	Myxococcales	Polyangiaceae	*Sorangium*	1	0	0	0	NO
Bacteria	Firmicutes	Bacilli	Bacillales	Planococcaceae	*Sporosarcina*	0	0	0	1	NO
Bacteria	Actinobacteria	Actinobacteria	Actinomycetales	Streptosporangiaceae	*Streptosporangium*	0	1	0	0	NO
Bacteria	Firmicutes	Clostridia	Clostridiales	Veillonellaceae	*Succiniclasticum*	0	1	0	0	NO
Bacteria	Actinobacteria	Actinobacteria	Actinomycetales	Propionibacteriaceae	*Tessaracoccus*	0	0	0	1	NO
Bacteria	Acidobacteria	Acidobacteria-6	BPC015			1	0	0	0	NO
Bacteria	Acidobacteria	S035				0	0	0	1	NO
Bacteria	Acidobacteria	TM1				1	0	0	0	NO
Bacteria	Actinobacteria	Actinobacteria	Actinomycetales	Actinosynnemataceae		1	0	0	0	NO
Bacteria	Actinobacteria	Thermoleophilia	Solirubrobacterales	Patulibacteraceae		0	1	0	0	NO
Bacteria	Bacteroidetes	Sphingobacteriia	Sphingobacteriales			0	1	0	0	NO
Bacteria	Chlorobi	BSV26	C20			0	1	0	0	NO
Bacteria	Chlorobi	BSV26	PK329			0	0	0	1	NO
Bacteria	Chloroflexi					0	0	0	1	NO
Bacteria	Chloroflexi	Anaerolineae	CFB-26			1	0	0	0	NO
Bacteria	Chloroflexi	Chloroflexi	[Roseiflexales]			0	1	0	0	NO
Bacteria	Chloroflexi	TK10				1	0	0	0	NO
Bacteria	Cyanobacteria	4C0d-2	SM1D11			0	0	0	1	NO
Bacteria	Firmicutes	Clostridia	Natranaerobiales	Anaerobrancaceae		0	0	0	1	NO
Bacteria	GN02	GKS2-174				1	0	0	0	NO
Bacteria	Nitrospirae	Nitrospira	Nitrospirales	[Leptospirillaceae]		0	0	0	1	NO
Bacteria	NKB19					1	0	0	0	NO
Bacteria	Planctomycetes	Planctomycetia	Pirellulales	Pirellulaceae		1	0	0	0	NO
Bacteria	Proteobacteria	Alphaproteobacteria	Rhizobiales	Rhizobiaceae		0	0	0	1	NO
Bacteria	Proteobacteria	Alphaproteobacteria	Sphingomonadales	Erythrobacteraceae		0	0	0	1	NO
Bacteria	Proteobacteria	Betaproteobacteria	Rhodocyclales	Rhodocyclaceae		1	0	0	0	NO
Bacteria	Proteobacteria	Deltaproteobacteria	[Entotheonellales]	[Entotheonellaceae]		1	0	0	0	NO
Bacteria	Proteobacteria	Deltaproteobacteria	FAC87			0	0	0	1	NO
Bacteria	Proteobacteria	Gammaproteobacteria	Enterobacteriales	Enterobacteriaceae		1	0	0	0	NO
Bacteria	Spirochaetes	GN05	SBYZ_6080			0	1	0	0	NO
Bacteria	Verrucomicrobia	[Methylacidiphilae]	S-BQ2-57			1	0	0	0	NO
Bacteria	Verrucomicrobia	[Pedosphaerae]	[Pedosphaerales]	auto67_4W		1	0	0	0	NO
Bacteria	Verrucomicrobia	Opitutae	Opitutales	Opitutaceae		0	1	0	0	NO
Bacteria	WS3	PRR-12	Sediment-1	PRR-10		1	0	0	0	NO
